# Enhanced Reactivity for the Wagner-Jauregg Reaction Under Aqueous Conditions

**DOI:** 10.1021/acsomega.5c07465

**Published:** 2025-12-28

**Authors:** Kaitlyn Barton, Chase Smith, Stephanie A. Tartakoff, Jason Stasio, Emma Rothe, Leana Dickhens, Adam D. Hill, Samuel S. Tartakoff

**Affiliations:** † Department of Chemistry, 2250 St. Lawrence University, Canton, New York 13617, United States; ‡ Department of Chemistry, 3757 Trinity College, Hartford, Connecticut 06106, United States

## Abstract

The Wagner-Jauregg reaction produces up to four new carbon–carbon bonds and eight new stereocenters, but limitations, such as high requisite temperatures, a restricted substrate scope, and significant undesired polymer formation, have resulted in this reaction receiving minimal attention. While generally considered a subtype of the Diels–Alder reaction, in which a styrenyl diene is employed in a tandem double-Diels–Alder reaction, a second, structurally distinct, stereochemically complex product can also be produced by the Wagner-Jauregg reaction, resulting from a Diels–Alder-ene cascade. Very few attempts to improve reaction conditions, expand substrate scope, or optimize product selectivity have been published. Through reaction optimization, supplemented by density functional theory calculations, we have explored the role of diene, dienophile, and solvent in controlling both the yields and product selectivity. This has led to the discovery of lower-temperature, aqueous reaction conditions that can produce the Diels–Alder-ene product, even with electron-poor dienes, with high selectivity and good yield. This work expands the understanding of the steric and electronic factors influencing product selectivity in [4 + 2]-cycloadditions and upgrades the reaction from a historical curiosity to a practical synthetic tool.

## Graphical abstract

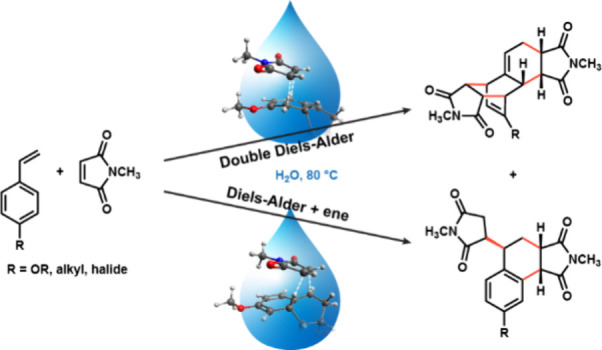

## Introduction

The formation of complicated carbon scaffolds from simple starting materials is critical to the production of pharmaceutical compounds;^1^ thus, the development of new methods for rapid synthesis of structurally complex small-molecules is vital. The Wagner-Jauregg (WJ) reaction^2,3^ (Scheme 1) seems to be a promising candidate for this purpose, as it can create up to eight new stereocenters from the reaction of substituted styrenes with an appropriate dienophile. However, high reaction temperatures, limited substrate scope, and concomitant formation of substantial amounts of polymeric side-products have prevented the reaction from being widely employed. While our recent work^4^ represented progress in overcoming some of those limitations, issues with reaction conditions and side-products remained a challenge. In this work, mechanistic studies and DFT calculations have yielded a vastly expanded scope for the WJ reaction, demonstrated control over which primary product is formed, substantially reduced unwanted polymer formation, and made green reaction conditions possible. The reaction can be run with good yields in aqueous solvent at 80 °C. These improvements will transform the WJ reaction from an idle curiosity to a valuable part of the synthetic organic toolkit.

**Scheme 1:**
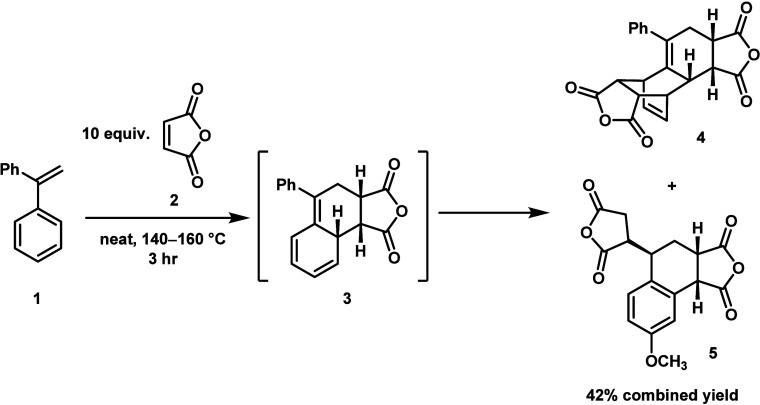
First Published Wagner-Jauregg Reaction

As demonstrated in our previous work, the WJ reaction proceeds via a mechanism analogous to the Diels–Alder (DA) reaction. The DA reaction, first described in 1928 by Otto Diels and Kurt Alder,^5^ features an electrocyclic [4 + 2]-cycloaddition with concomitant establishment of up to four stereocenters. Nearly a century has passed since that seminal paper was published and in that time, factors controlling stereo- and regioselectivity in the DA are now well understood,^6–8^ catalytic^9,10^ and enantioselective^11,12^ DA reactions have been developed, and numerous variations have been reported (ex. inverse-demand DA,^13^ hetero-DA,^14^ and homo-DA).^15^ The utility of the DA reaction has been showcased in the synthesis of complex molecular targets^16^ and is discussed in most introductory organic chemistry textbooks and classrooms.^17,100^

By contrast, the closely related WJ reaction, which was discovered just 2 years after the DA reaction, has received comparatively little attention or study. There are several reasons for this: the original WJ reaction required high temperatures in the range of 120–200 °C, multiday reaction times, and high concentrations, leading to large amounts of polymer formation and very low yields of the desired double-DA (DDA, **4**) product. Indeed, the initial publication resulted from observations about minor side-products that were found in industrial polymerization reactions. Early efforts to study the WJ reaction were further complicated by a second product with identical molecular weight to the DDA product.^18^ Several researchers reported variable amounts of these two products, even when using similar reaction conditions and identical substrates.^19,20^ The second product (**5**) was eventually identified as resulting from an initial DA reaction, followed by a subsequent ene reaction.

Conjectures were made about the reaction mechanism and the reasons why 1,1-diarylethylenes appeared to be uniquely reactive in the WJ reaction. However, later work showed that some electron-rich, monocyclic styrene derivatives could react as well as, or better than, the original 1,1-diarylethylenes. Work by Kovacs et. al further showed that the addition of radical inhibitors, most notably *N*,*N*-dimethylaniline (DMA) or picric acid, could increase the yield of the DDA and DA-ene products.^18^

Since the 1940s, several research groups have utilized the WJ reaction, with multiple examples featuring tethered styrene derivatives.^21–24^ Other researchers have developed mechanistically distinct methods for producing the same types of polycyclic products^25,26^ but the WJ reaction remains largely under-investigated and underutilized, despite its potential value. The DDA pathway represents the formation of four C–C bonds, two new rings, and up to eight stereocenters in a single reaction step. The DA-ene product results in both a formal electrophilic aromatic substitution reaction and benzylic C–H activation under relatively mild reaction conditions. The potential synthetic utility of such a complexity-building reaction is obvious, provided that the WJ reaction can produce those products reliably and predictably.

While our previous efforts to clarify the WJ reaction mechanism confirmed that it matched that of the standard DA and improved somewhat on the reaction conditions, in terms of reducing the requisite reaction temperatures and increasing yields for some substrates, the substrate scope was still limited to electron-rich dienes, isolation of DDA products was difficult, and no DA-ene products were observed under our reaction conditions. Also, prior work did not reveal a single set of reaction conditions that afforded satisfactory yields of all products studied.^4^ With an aim toward expanding the scope and applications of this reaction, we undertook further experimental and computational studies of the WJ reaction. The current findings reveal that the reaction can be accomplished in water at 80 °C, with both minimal formation of side-products and control over DDA/DA-ene product ratios, opening the way for wider application of the WJ reaction.

## Results and Discussion

The utility of the WJ reaction can be expanded with a fuller understanding of the factors influencing the yields of DDA and DA-ene products. Three material factors chiefly control this outcome: solvent, dienophile, and diene. We initially optimized the first two factors before moving on to explore the effect of diene substitutions.

### Solvent Effects and Impact of Dienophile on Product Ratio

All early reports of the WJ reaction were either run neat (the maleic anhydride dienophile became a molten solvent at the reaction temperature) or at high concentrations in high-boiling solvents. Our previous paper^4^ investigated a number of common organic solvents with maleic anhydride as the dienophile and found that DMSO was superior, as far as minimizing polymer formation, reducing reaction times, and increasing yield. Similar to previously published findings,^27^ the benefits of a polar solvent were largely attributable to increased solubility of both starting materials and products, which resulted in more homogeneous reaction conditions, more even heating, and dilution of the reactants, such that polymerization was less likely. It is noteworthy that in our previous studies, no DA-ene product was observed. This was explained by our DFT studies, which revealed that the ΔG^‡^ of the second DA transition state was significantly lower than that of the ene transition state. A further limitation of this earlier work was the production of dimethylsulfide, building up a potentially dangerous amount of internal pressure as DMSO decomposed. Nucleophilic, protic solvents were not an option due to the strongly electrophilic nature of maleic anhydride and the ease with which that dienophile can be solvolyzed. These limitations highlighted the need to search for new conditions in order to optimize for the DA-ene product.

Other researchers had previously utilized maleimide derivatives as dienophiles in the WJ reaction,^23,28,29^ although due to their decreased reactivity relative to maleic anhydride and the already-substantial activation energy required for dearomatization, they have received comparatively little attention. However, we felt that the increased solvolytic stability of the maleimide ring, relative to that of the anhydride, might be a useful feature and allow us to study new facets of the WJ reaction. Preliminary DFT calculations supported that the second DA and ene transition states are closer in energy (reacting 4-methoxystyrene with *N*-methylmaleimide in water exhibits a difference of 0.95 kcal/mol versus 3.75 kcal/mol for the reaction of maleic anhydride in DMSO), affording access to the DA-ene products (Figure S1). The identity of the maleimide dienophile shifted the DFT-calculated ΔΔG^‡^ values from favoring the ene pathway by 0.7 kcal/mol to favoring the DA pathway by 2.6 kcal/mol (Figure S2), implying that the solvent and maleimide identity could be used to help control which product is formed. As anticipated from the increased Δ*G* ^‡^ values of the rate-determining first addition, using N-substituted maleimides with the previously optimized reaction conditions significantly decreased the WJ reaction rates relative to the earlier maleic anhydride reactions. Given the reduced electrophilicity of the imide dienophiles and the dramatically different physical properties (e.g., solubility), it seemed reasonable to further optimize the reaction conditions for the WJ reaction with these dienophiles using a variety of polar protic and aprotic solvents (Table 1). For this solvent screen, electron-rich diene **6** was employed as the test substrate due to its low cost and fast reaction relative to other commercially available styrene derivatives.

**Table 1:**
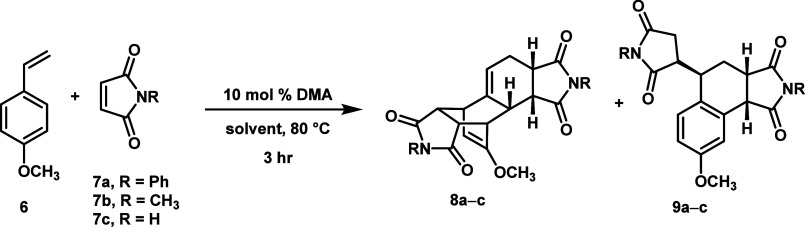
Results for Solvent Screen of Wagner-Jauregg Reaction^a^

solvent	dielectric constant (ε)	8a:9a	ratio of DDA-to-ene 8b:9b	8c:9c
H_2_O	80.1	1.31 (99%)	0.538 (100%)	4.00 (94.4%)
DMSO	46.7	1.11 (97%)	0.496 (59%)	0.900 (42%)
DMF	36.7	1.12 (96%)	0.409 (57%)	1.76 (79%)
EtOH	24.5	1.29 (100%)	0.455 (98%)	1.06 (92%)
iPrOH	17.9	1.57 (87%)	0.441 (96%)	1.35 (trace)
PhCH_3_	2.38	1.01 (80%)	0.350 (58%)	1.37 (94%)
Cyclohexane	2.02	1.04 (75%)	0.331 (48%)	1.49 (85%)

a All experiments were conducted at 1.8 M styrene concentration, in the specified solvent, with 5 equiv of dienophile (**7a**–**c**). Percent conversion (based on unreacted styrene, as determined by 1H NMR analysis) is given in parentheses. Dielectric constants are given for 20 °C.

As previously observed with maleic anhydride, sufficiently high-boiling, nonpolar solvents (toluene, cyclohexane) generally gave both slower reactions and increased amounts of polymeric side-products, including polymaleimide, polystyrene, and copolymers, with large variability between replicate reactions when using imides **7b** and **7c**. In the case of alcoholic solvents, the DA-ene products proved to be much more soluble than the DDA products, resulting in more variability in yield and product ratios when trying to replicate the results. Additionally, with imide **7a** in EtOH, solvolytic ring-opening of the imide led to complex reaction mixtures and reduced overall yields, despite high conversion rates. This was presumably due to the increased electrophilicity of the N-Ph moiety. In *i*PrOH, solvolysis was also observed, albeit to a much lesser extent. The rate of this solvolysis reaction appears to be greater for DA-ene adduct **9a** than for DDA adduct **8a**, which we intend to investigate more fully in the future.

The cleanest reactions (minimal polymeric side-products) were generally achieved in more polar solvents (DMF, DMSO, and H_2_O). While DMF worked reasonably well, significant amounts of polymaleimide were still observed, and there was more variability between replicates when compared with DMSO. As previously observed with maleic anhydride, DMSO still performed well for dienophile **7a**, but much lower reaction rates were observed with **7b** and **7c.**

H_2_O gave the most consistent results, high conversion, and lowest amounts of polymer (none observed with imides **7a** or **7b**), despite neither starting materials nor products being visibly soluble under our reaction conditions. Unlike DMSO or DMF reactions, which became largely homogeneous when heated at 80 °C in a sealed vial, the reactions in H_2_O formed a biphasic mixture with a molten organic layer below the aqueous phase, although magnetic stirring somewhat agitated and mixed the two layers. Because of the low solubility of both reactants and products in water, it seemed reasonable that a neat reaction (similar to the original WJ reactions with anhydride **2**) would give similar results, with or without the addition of stoichiometric H_2_O. However, while neat reactions of **6** with **7b** did consistently give **8b**:**9b** ratios of 0.33 (similar to reactions in cyclohexane), the percent conversion was variable, ranging from 25 to 55% and significant amounts of polymer were formed. When 1–2 equiv of H_2_O were added to solvent-free reactions, product ratios again remained consistent (at a 0.33 ratio of **8b**:**9b**) and nearly all starting styrene was consumed but polymeric products were again the dominant species formed. Clearly, having superstoichiometric H_2_O in this reaction plays a vital role. This, perhaps, should not be surprising, as it has been reported that water can accelerate certain intermolecular reactions, particularly the DA reaction, via a hydrophobic effect,^30,31^ whereby the entropic cost for the reaction is decreased.^32^

Indeed, the DDA product became more dominant as the solvent polarity increased (Figure 1). We excluded the reactions of imide **7a** in alcoholic solvents from this analysis, as the variable rate of solvolysis for the two different products and variability from experiment to experiment made measurement of the product ratios unreliable.

**Figure 1:**
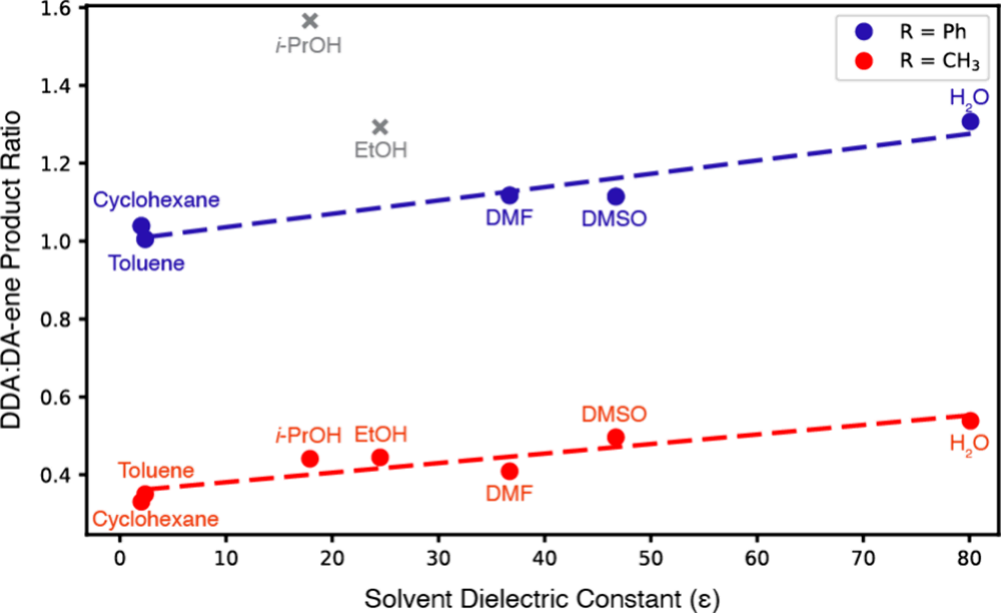
Influence of solvent polarity on the DDA/DA-ene ratio for dienophiles **7a** and **7b**. The alcoholic solvents with dienophile **7a** (marked as gray Xs) were excluded from the analysis due to solvolytic decomposition of products **8a** and **9a**, making the measured product ratio inconsistent and unreliable.

To better understand this trend, we turned to DFT calculations for additional insight into the pathways leading to both the DDA and DA-ene products. The combination of ωB97XD density functional, cc-pVDZ basis set, and a polarizable continuum model (PCM) performed with sufficient accuracy for our earlier work,^33–35^ but initial calculations for this study performed with the cc-pVDZ basis set showed poor correspondence with the new experimental results. Switching to the larger aug-cc-pVDZ basis improved correlation to experimental observations; aug-cc-pVDZ was subsequently used for all calculations. We compared the two different reaction pathways (Figure 2). These calculations determined the Δ*G* for addition of the second equivalent of **7b** to be 0.95 kcal/mol lower in energy than the corresponding transition state for the DA-ene pathway; this value agrees within typical DFT errors with experimentally observed DDA/DA-ene ratios,^36,37^ which suggest ΔΔ*G* ^‡^ values of 0.2–0.4 kcal/mol in water (using the Eyring-Polanyi equation and assuming equivalent κ values.)

**Figure 2:**
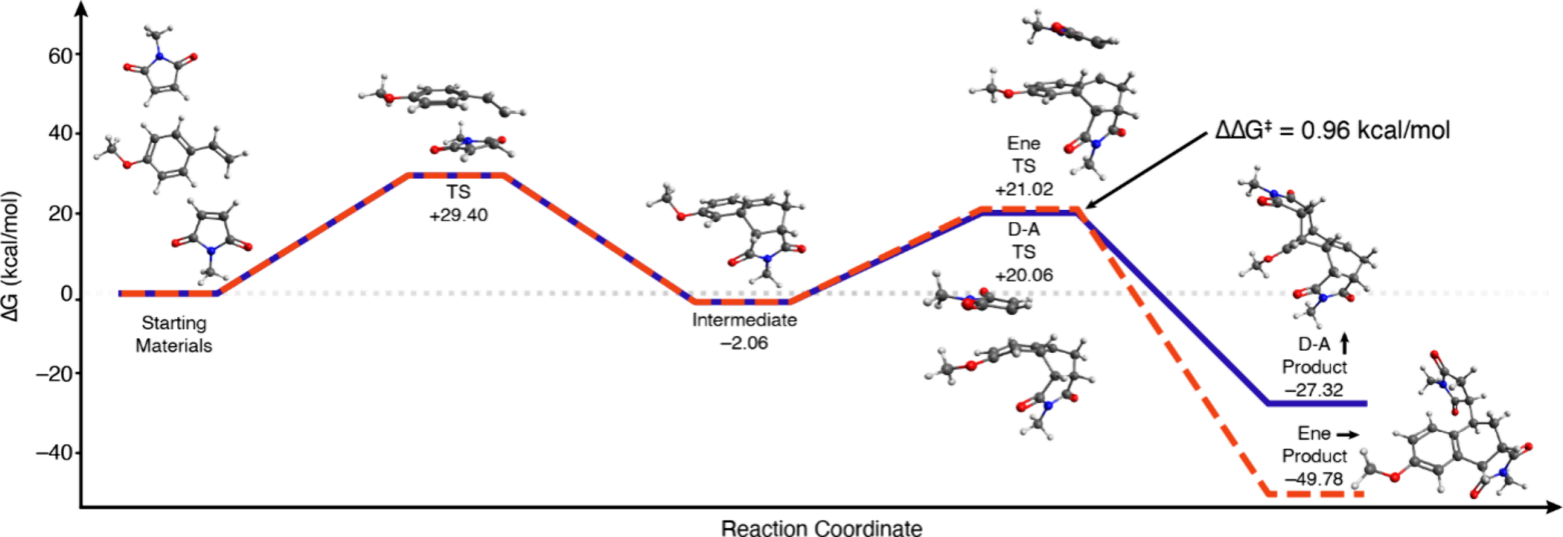
Reaction coordinate Δ*G* diagram of the conversion of **6** and **7b** into **8b** and **9b**, as calculated using DFT with the aug-cc-pVDZ basis set, demonstrating energetic similarity of the ene and DA transition states for the second maleimide addition despite the position of maleimide **7b** in the ene and DA transition states differing.

Some of the modest discrepancies between theory and experiment may stem from the polarizable continuum model, which has two potential weaknesses. It assumes that the local solvent environment matches the bulk, which may be inaccurate for solvents like water in which conditions are not fully homogeneous. The PCM also lacks explicit solvent molecules and, therefore, does not predict or model the Δ*S* for the surrounding solvent; solvation of hydrophobic moieties like the methyl and phenyl groups of dienophiles **7a** and **7b** is expected to have a substantial entropic penalty.^38^ The possible role of this solvent entropy effect can be qualitatively understood by considering the total solvent-exposed surface area of the transition states for the diene-dienophile pairs (Figure 3). With imides **7a** and **7b**, the transition state for the second DA shows a more solvent-exposed surface relative to the ene transition state, where the dienophile has slipped farther back on the rest of the ring system, extending the hydrophobic moiety farther out into the solvent.

**Figure 3:**
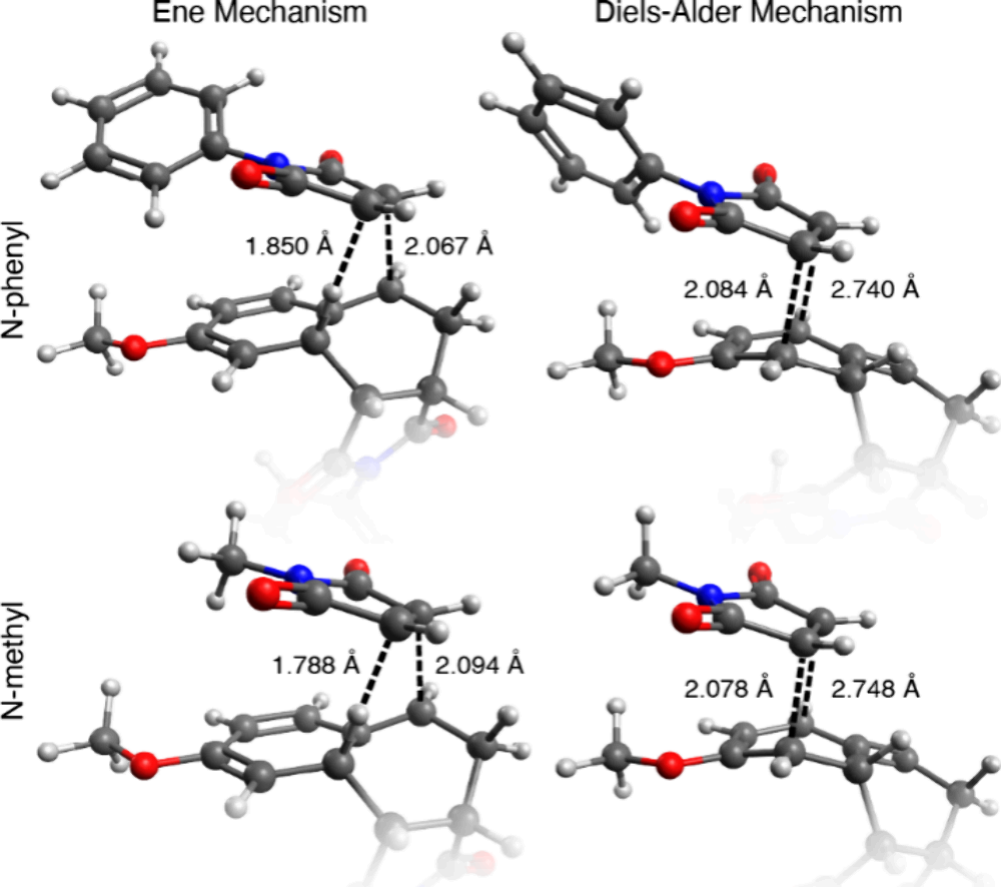
Comparison of DFT-calculated transition states for the second addition of *N*-phenylmaleimide (top) or *N*-methylmaleimide (bottom) via the ene (left) or DA (right) mechanism, demonstrating significantly greater hydrophobic surface area of DA transition states.

Despite solvent-dependent changes to reaction rates and product ratios, the DA-ene product **9a** was always the major product with imide **7a**, while **8b** was always the major adduct with **7b**. With dienophile **7c**, there was no clear pattern in the product ratios, possibly due to complications with hydrogen bonding, although **8c** was the major product in all solvents except DMSO. With such a large predicted ΔΔ*G* between the DDA product **8b** and DA-ene product **9b** (which would be expected based on the stability gained by rearomatization of the DA-ene product), if the transition state energies were actually similar, one might expect that **8b** could be converted into **9b** under appropriate conditions. However, conversion of isolated **8b** into **9b** was never observed, even after prolonged heating (up to 200 °C), with or without the addition of various Lewis acids or bases. DFT ΔG^‡^ energies for the reverse reaction fall in the 46.6–51.0 kcal/mol range; if no other energetically accessible rearrangement pathways are available, DFT results are consistent with the absence of observed rearrangement from the DDA to DA-ene products.

The success of H_2_O as a solvent for the WJ reaction affords several benefits, with the most obvious being low cost and improved safety. Additionally, the insolubility of the reaction products in H_2_O allows for isolation of the crude reaction mixtures via simple vacuum filtration, whereas reactions that were run in DMSO required multiple extraction steps before the reaction mixture was ready for further purification of the DDA and DA-ene products. The improvements in yields, beyond general synthetic utility, also allowed us to gain additional mechanistic insights.

### Diene Scope and Impact on Product Ratio

With the WJ reaction running at higher yields, substrates that previously seemed to produce no reaction could be explored, highlighting better the role of different *para* substitutions. Early work^39^ suggested that halogenated styrene derivatives afforded little or no desired products, which is reasonable given modern understanding concerning the electronic influences for the DA reaction. Furthermore, our own previous work^4^ with maleic anhydride showed sluggish reactions and low yields with halogenated dienes, and no observed product with very electron-poor styrenes. However, given the rate acceleration that we had observed with aqueous reaction conditions, it seemed reasonable to re-examine the substrate scope (Table 2) and compute the corresponding reaction pathways (Figure S3).

**Table 2:**
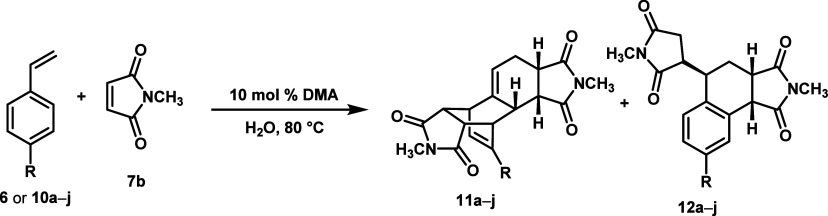
Product Ratios for Various Para-Substituted Styrenes^a^

	*R* =	time to completion (h)	Hammet value (σ)	DDA/DA-ene 11a–j/12a–j
**6**	OCH_3_	3	–0.27	35:65: **(8b:9b)**
**10a**	OBn	6	–0.25 (est.)	34:66
**10b**	*t*Bu	18	–0.20	0:100
**10c**	CH_3_	12	–0.17	38:62
**10d**	H	24	0.00	5:95
**10e**	Ph	24	0.01	14:86
**10f**	F	24	0.06	10:90
**10g**	Cl	24	0.23	12:88
**10h**	Br	24	0.23	14:86
**10i**	CO_2_CH_3_	48–72	0.45	16:84
**10j**	CF_3_	168*	0.54	0:100

a All experiments were conducted with 100–200 mg of diene **6** or **10a–j** and 5 equivalents of imide **7b** in 0.4 mL of H_2_O. Time to completion is approximate, based on complete consumption of diene. *55% conversion based on unreacted styrene.

Hammett substituent constants^40^ were used to examine possible trends regarding the impact of electronic structure of the dienes on the product ratios, although no close correlation was observed despite the correlation between σ and ΔΔ*G* ^‡^ (Figure 4). All 4-substituted styrene derivatives produced DA-ene adduct as the major product, with electron-rich dienes (**6**, **10a,** and **10c**) being the only substrates to show significant amounts of DDA product. Steric factors clearly had a strong influence on the second DA reaction, as **10b** afforded no DDA and is a clear outlier to the trend shown in Figure 4. At the same time, very electron-poor dienes (**10j**) presumably increased the activation energy for the second DA reaction to the point that no DDA product was observed. Indeed, based on computational results, **10b** and **10j** were the two substrates with the largest second transition state ΔΔ*G* ^‡^ values, favoring the ene pathway by 4.70 and 3.38 kcal/mol, respectively. For substrates where the DA-ene was moderately favored over the DDA (**10f–i**), calculated ΔΔ*G* ^‡^ values between 2.7 and 3.2 kcal/mol were found.

**Figure 4:**
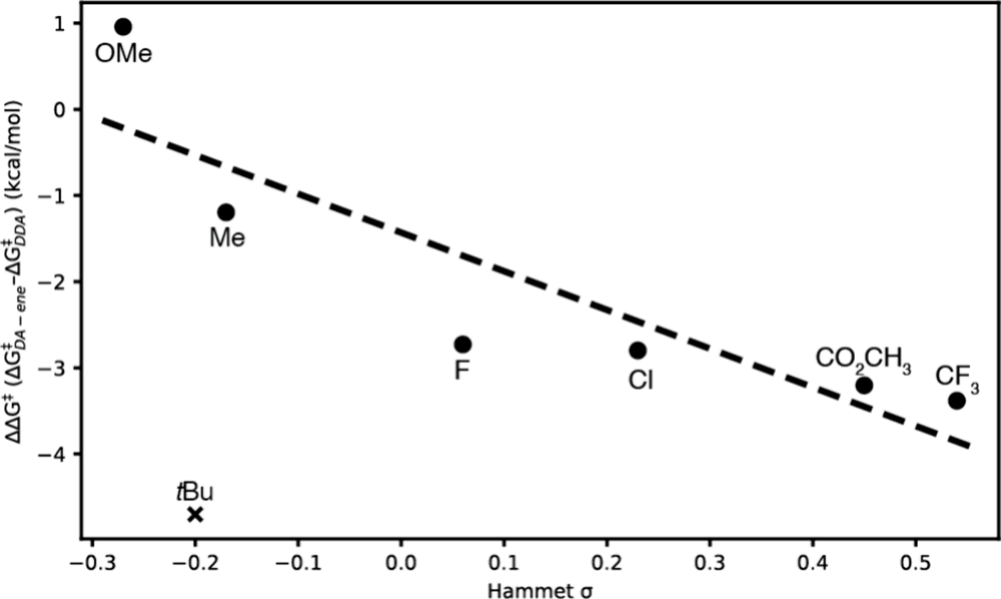
DFT-calculated ΔΔ*G* ^‡^ versus literature Hammet σ for each styrene substituent, showing expected linear correlation if sterically limited *t*-butyl outlier (X) is excluded.

Under aqueous conditions, the WJ reaction proved effective for a substrate scope far broader than typically expected. The fact that **10d** gave significant amounts of either product is surprising, as it had previously performed very poorly with maleic anhydride and had been reported elsewhere to predominantly give unwanted side-products in similar reactions.^26,43^ Likewise, all of the halogenated styrene derivatives (**10f–h**) performed better than previously observed and very electron-poor styrenes (**10i–j**), which had failed to react at all with maleic anhydride under previous conditions, gave serviceable yields of DA-ene products, although **10j** did not go to completion, even after extended heating.

Styrene **1**, which had been used in the seminal WJ studies and publications, produced exclusively the DDA product under our reaction conditions (Scheme 2). This is not terribly surprising, given that the second phenyl ring offers significant steric hindrance to the ene transition state while leaving the second DA reaction unobstructed. However, this result is in sharp contrast to the variable mixtures of DDA and DA-ene products that had been reported in early WJ studies, suggesting that our new reaction conditions give more consistent, predictable results.

**Scheme 2:**
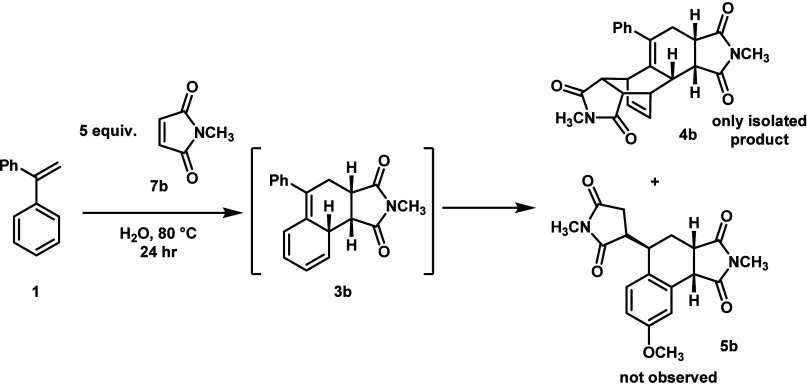
Wagner-Jauregg Reaction with Original Diene Under New Reaction Conditions

Given the agreement generally observed between DFT results and the observed ratios, we sought to more directly quantify the correlation between theory and experiment. As the first maleimide [4 + 2]-addition is rate-determining for all substrates (Figure S3), the DDA:DA-ene product ratio following the second addition should be equal to the ratio of rate constants for the second step; this, in turn, can be determined from a rearrangement of the Eyring-Polanyi equation:^41,42^

$$\frac{k_{DDA}}{k_{DA-ene}}=\frac{\kappa_{DDA}}{\kappa_{DA-ene}}e^{\left(\Delta G_{DA-ene}^{‡}-\Delta G_{DDA}^{‡}\right)/RT}$$ 1

A comparison of computed ΔΔ*G* ^‡^ values and experimental ratios shows the expected positive correlation (Figure 5), though fitting eq 1 to the data resulted in relatively high uncertainties (2σ, 95% confidence interval): a κ_DDA_/κ_DA‑ene_ ratio of 0.4 ± 0.2 and temperature of 1500 ± 1200 K. This weaker correlation suggests that the role of either solvent thermodynamic contributions (e.g., decrease in the entropy of water due to solvating hydrophobic moieties) or inner-sphere interactions (e.g., water hydrogen-bonding interactions with the substrate) in determining reaction outcomes under aqueous conditions may be considerable, which is consistent with the relatively unique capabilities of water as a solvent for the WJ reaction (Table 1). Nonetheless, the general magnitude of each parameter may provide some insight into why the reaction deviates from the assumptions in the DFT model. That κ_DDA_/κ_DA‑ene_ <1 suggests that the transmission coefficient of the DA mechanism may be smaller; however, given the literature values of κ for DA reactions are typically near 1,^44–46^ this difference may instead be due to a tunneling-related speed-up in the proton transfer during the ene reaction.^47^ The substantial uncertainty in temperature parameter means that the experimental temperature (353 K) falls within the 95% confidence window; nonetheless, the effect of a large temperature parameter matches the trend that product ratios appear to show a weaker-than-expected dependence on the ΔΔ*G* ^‡^ value. This could be indicative of the role of solvent entropy neglected by our DFT model but also matches the expected trend if tunneling were influencing the rate of the ene reaction. Uncertainty in our data does not allow for the relative contributions of solvent and tunneling effects to be fully quantified via a more sophisticated equation or theory but does raise interesting possibilities for further optimizations of the WJ reaction in the future.

**Figure 5:**
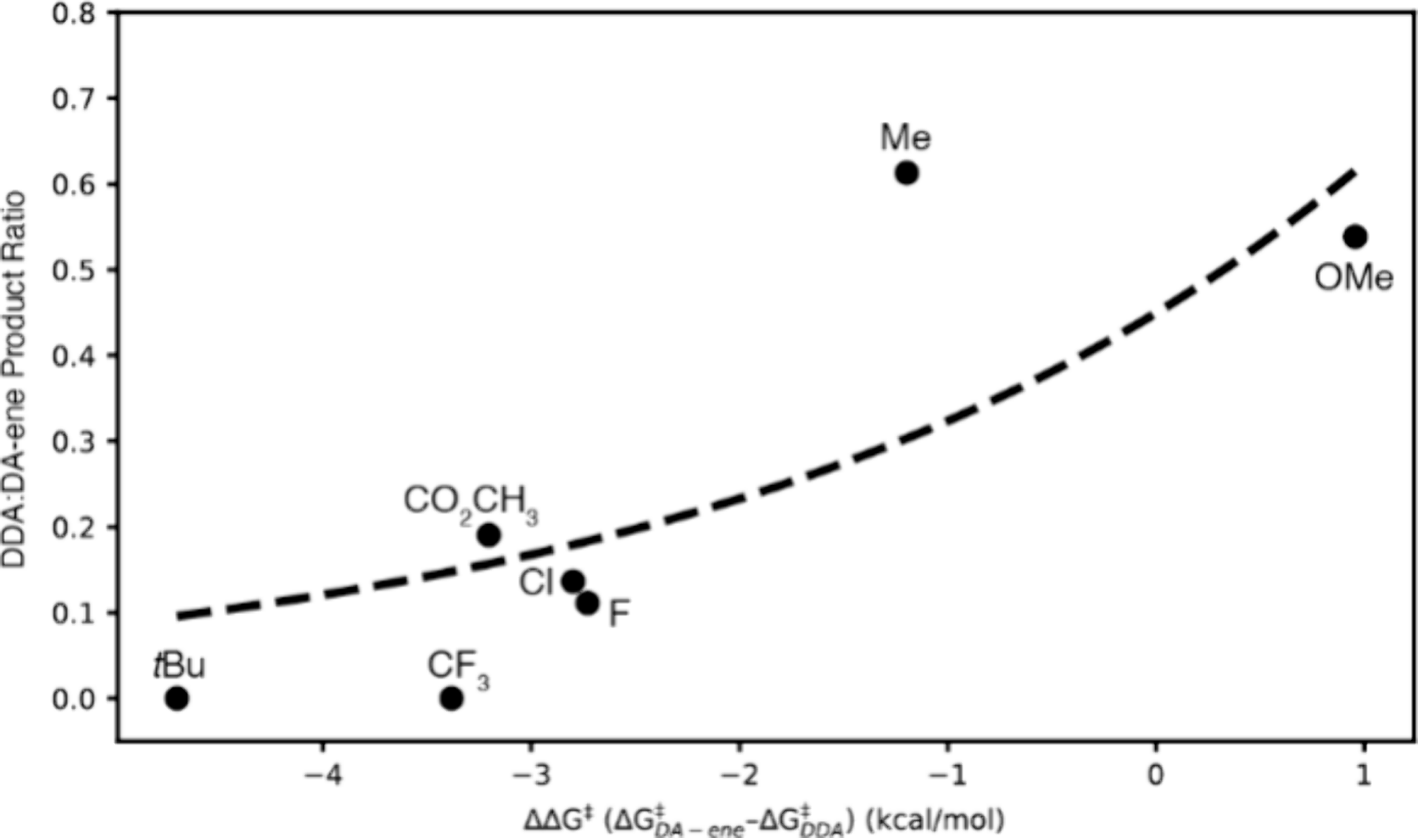
Experimentally determined DDA:DA-ene product ratio for the reaction of *N*-methylmaleimide with substituted styrenes versus DFT-computed ΔΔ*G* ^‡^ values (aug-cc-pVDZ basis) shows the correlation predicted by the Eyring-Polanyi equation.

## Conclusions

Building on previous work to optimize the Wagner-Jauregg reaction and expand its utility, we successfully employed several maleimide dienophiles with a variety of styrenyl dienes in an array of solvents. These studies, in combination with DFT modeling, have revealed mechanistic factors influencing the formation of either a double-Diels–Alder or Diels–Alder-ene product. Furthermore, we have developed aqueous reaction conditions which, in addition to being cheaper, milder, and more environmentally benign than previous conditions, also work well for electron-poor styrene derivatives and reliably produce the DA-ene product with minimal polymeric side-product.

While questions remain regarding whether this reaction is occurring in the aqueous phase or at the solvent interface, water clearly plays a critical role in the reaction and has resulted in improved reaction outcomes. Moderate correlation between calculated ΔΔ*G* ^‡^ values and observed product ratios suggest that entropic effects in the solvent, inner-sphere hydrogen bonding, or tunneling in the ene pathway may play a role in biasing reaction outcomes. Further exploration of the role of water in this reaction is warranted, using both experimental and computational strategies as well as testing the value of these reaction conditions on polysubstituted styrene derivatives and other substrates that have been previously reported to not work well in the Wagner-Jauregg reaction.

## Methods

### General Experimental

Styrene **10a** was synthesized according to literature procedures^48^ and characterized by ^1^H and ^13^C NMR spectroscopy. All spectra were consistent with a previously reported value. Several commercial styrenes (**10c**, **10d**, **10f**, **10g**, and **10h**) were supplied with varying amounts (>1%) *tert*-butylcatechol as a radical inhibitor, and no effort was made to further purify those styrenes. All other reagents were purchased from commercial sources and used without further purification. Silica gel used for chromatographic separation (60 Å, 230–400 mesh) was purchased from Sigma-Millipore. Thin-layer chromatographic (TLC) analysis was conducted using glass-backed EMD/Millipore silica-gel plates (60 Å, 230–400 mesh). All ^1^H NMR spectra were collected using a 400 MHz JEOL-ECZ400S NMR spectrometer (101 MHz on the ^13^C channel) and referenced using the residual solvent peaks: DMSO-*d*_6_ (2.50 ppm for ^1^H and 39.51 ppm for ^13^C), acetone-d_6_ (2.05 ppm for ^1^H and 206.26, 29.84 ppm for ^13^C), and CDCl_3_ (7.26 ppm for ^1^H and 77.16 ppm for ^13^C). Mass spectral data was collected using an Agilent 7820A GC/MSD (EI) or AB Sciex AB4000 (ESI).

### Solvent Screen

Wagner-Jauregg reactions were performed using conditions similar to those previously reported by the Tartakoff group.^4^ Styrene **6** (100 mg, 0.75 mmol, 1.00 equiv) was added to a one-dram vial with a screw-top lid and a small stir bar, then diluted with the appropriate solvent (0.4 mL). Crushed dienophile **7a–c** (3.72 mmol, 5.00 equiv) was added to the vial, along with DMA (1 drop, ∼0.08 mmol, ∼0.1 equiv). The vial was capped tightly and placed in a preheated aluminum block (at 80 °C) on a hot plate while stirring (∼350 rpm) for 3 h. Once the reaction was complete, a few drops of the crude mixture (the bottom layer in cases where the mixture was biphasic) were removed by pipette, added directly to an NMR tube, diluted with DMSO-*d*_6_, and analyzed by ^1^H NMR. Product ratios were determined by integration of well-resolved, diagnostic peaks (styrene **6** vinyl doublets: 5.55–5.67 and 5.05–5.15 ppm; vinyl peaks from DDA products **8a–c**: 5.60–5.85 and 4.55–5.00 ppm; benzylic doublet from DA-ene products **9a–c**: 4.11–4.48). Percent conversion was determined by dividing unreacted styrene **6** by the sum of **6**, **8a–c**, and **9a–c.**

### Product Ratio Determination

Analogous to the solvent screen, styrenes **10a–j** (100 or 200 mg, 1.00 equiv) were added to a one-dram vial with a screw-top lid and a small stir bar, then diluted with H_2_O (0.4 mL). Crushed dienophile **7b** (5.00 equiv) was added to the vial, along with DMA (1 drop, ∼0.08 mmol, ∼0.1 equiv). The vial was capped tightly and placed in a preheated aluminum block (at 80 °C) on a hot plate while stirring (∼350 rpm) until all styrene had been consumed (preliminary runs were used to determine the appropriate amount of time, since unsealing vials had a marked impact on reaction homogeneity and outcome.) Once the reaction was complete, a few drops of the crude organic layer were removed by a pipette, added directly to an NMR tube, diluted with DMSO-*d*_6_, and analyzed by ^1^H NMR. Product ratios were determined by integration of well-resolved, diagnostic peaks, as described above.

### Computational Modeling and Analysis

Density functional theory (DFT) calculations were used to model the plausible reaction pathways leading to both the DA and ene products. All calculations were performed using Q-Chem 5.2 on St. Lawrence University’s Ada high-performance computer, a Warewulf Community Support Cluster system.^49^ The ωB97XD density functional was used for all computations,^34^ along with a polarizable continuum model to simulate the solvent at reaction temperature (ε = 58.3 for water,^50^ ε = 41.8 for DMSO;^51^ most calculations were performed in water unless indicated otherwise.) Initial structure optimizations were performed using cc-pVDZ and aug-cc-pVDZ basis sets, followed by harmonic frequency calculations to verify that the structure represented a true local minimum or transition state (absent any excess imaginary vibrations.) Calculated frequencies were also used to determine Δ*H* and Δ*S* corrections at 80 °C and, after correcting Δ*S* for changing numbers of molecules over the course of the reaction, combined with the structure energies to determine the Gibbs-free energy of each point along the reaction coordinate. Thermodynamic comparisons and plotting were performed by using Python 3.9.13 with numpy 1.26.2 and matplotlib 3.8.0 via the Spyder 5.4.3 IDE.

## Supplementary Material



## Notes

DFT-calculated reaction coordinates (S2–S3); thermodynamic values and structure xyz coordinates (S4–S58); additional experimental procedures (S59–S62); ^1^H and ^13^C NMR spectral data for new compounds (S63–S103) (DOCX)
