# Supplementary material for: Enhanced Reactivity for the Wagner-Jauregg Reaction Under Aqueous Conditions
Source: ACS Omega. 2025 Dec 28;11(1):1360–8. doi: 10.1021/acsomega.5c07465 (PMC12809351; doi:10.1021/acsomega.5c07465)
Supplement: Supplementary file 1 [file ao5c07465_si_001.pdf]

Supporting Information

Enhanced Reactivity for the Wagner-Jauregg Reaction Under Aqueous Conditions

Kaitlyn Barton, Chase Smith, Stephanie A. Tartakoff, Jason Stasio, Emma Rothe, Leana Dickhens, Adam D. Hill, Samuel S. Tartakoff

St. Lawrence University, Canton, NY 13617, United States

Trinity College, Hartford, CT 06106, United State

Table of Contents

Appendix A. DFT Computational Results....S2
    Supplementary DFT Figures....S2
    DFT Structures and Associated Energies....S4
Appendix B. Characterization for Wagner-Jauregg Products....S59
Appendix C. ^{1}H and ^{13}C NMR Spectra of Purified Wagner-Jauregg Products....S63

Appendix A: DFT Computational Results

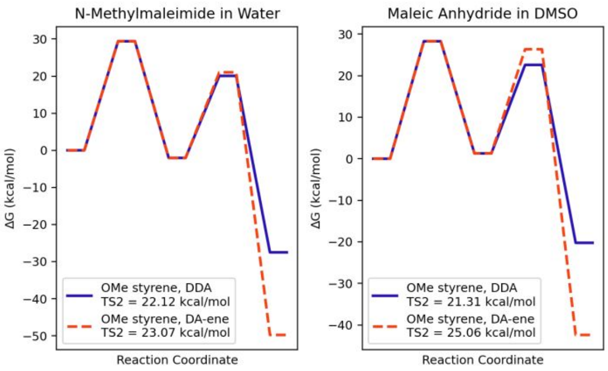

Figure S1. DFT-calculated reaction pathways (aug-cc-pVDZ basis) for reactions of 4-methoxystyrene with maleimide (blue and orange lines), N-methylmaleimide (green and red lines), and N-phenylmaleimide (violet and brown lines) following double Diels−Alder pathway (solid lines) or Diels−Alder/ene pathway (dashed lines). TS2 energies represent the increase in energy from the intermediate structure to the second transition state along each path.

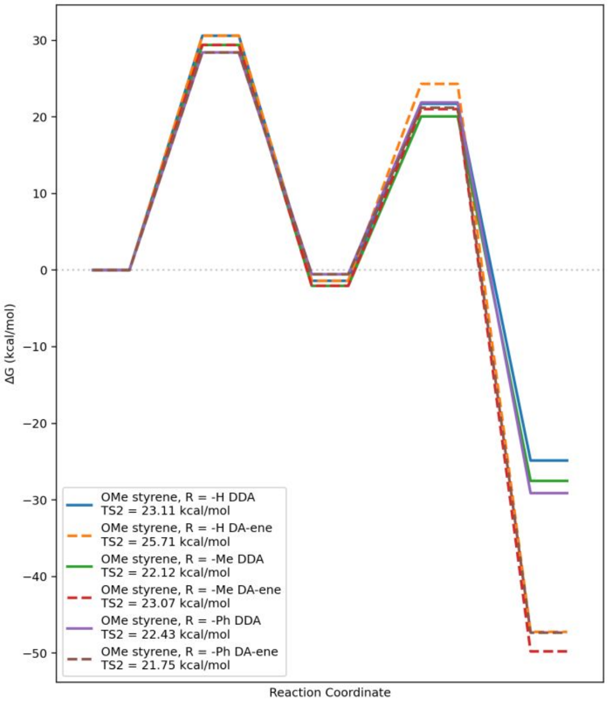

Figure S2. Comparison of DFT-calculated reaction pathways (aug-cc-pVDZ basis) for 4-methoxystyrene reacting with n-methylmaleimide in water (left; focus of this work) versus reacting with maleic anhydride in DMSO (right; focus of Tartakoff et. al. 2020), demonstrating that the change in dienophile and solvent changes the preference of the system for double-Diels−Alder (solid line) or Diels−Alder/ene (dashed line) products. TS2 energies represent the increase in energy from the intermediate structure to the second transition state along each path.

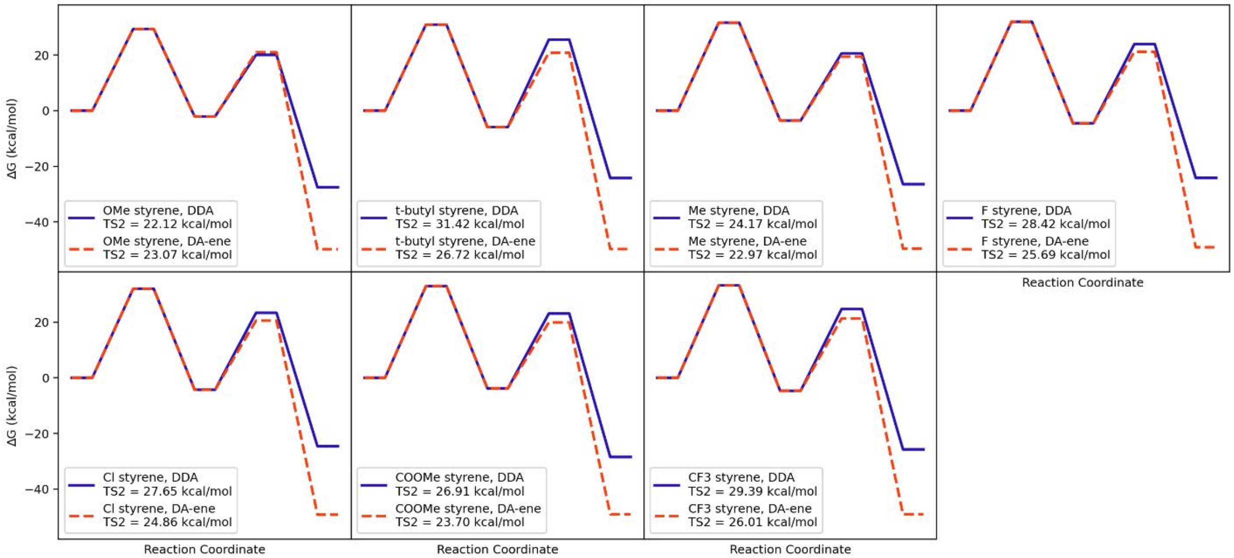

Figure S3. DFT-calculated reaction pathways (aug-cc-pVDZ basis) for reactions of 4-substituted styrenes with N-methylmaleimide; −double Diels−Alder pathway is shown as solid indigo line and Diels−Alder/ene pathway is shown as dashed vermillion line. TS2 energies represent the increase in energy from the intermediate structure to the second transition state along each path.

DFT Structures and Associated Energies

Energy, thermodynamic quantities, and corresponding xyz coordinates for experimental compounds. All calculations were performed with a polarizable continuum ε = 58.3 (water at experimental temperature) unless otherwise noted.

Table S1. Energy, thermodynamic quantities, and corresponding xyz coordinates (in Angstroms; calculated with ωB97XD density functional and aug-cc-pVDZ basis set) of 4-chlorostyrene N-methylmaleimide intermediate.

Energy = -1167.903726 hartrees
Total enthalpy = 153.16 kcal/mol
Total entropy = 119.55 cal/mol×K

ATOM	X	Y	Z
1 C	-0.3394300000	-1.4278200000	0.4244000000
2 C	-1.3673500000	-1.3022100000	1.4496100000
3 C	-2.4645200000	-0.5458400000	1.2546200000
4 C	-2.6564700000	0.1880700000	-0.0009200000
5 C	-1.7610200000	0.1262600000	-0.9975600000
6 C	-0.5175300000	-0.7091100000	-0.9022600000
7 C	0.7849600000	-2.1503800000	0.5969600000
8 C	1.8023200000	-2.1848900000	-0.5033100000
9 C	2.0577500000	-0.7639700000	-1.0455900000
10 C	2.8721700000	0.0413700000	-0.0471100000
11 C	0.7818000000	0.0700400000	-1.2664900000
12 C	0.9801300000	1.3130700000	-0.4134800000
13 H	0.6961300000	0.4082800000	-2.3091800000
14 H	-0.5852200000	-1.4832400000	-1.6922300000
15 H	0.9626700000	-2.6952100000	1.5276000000
16 H	-1.2111000000	-1.8257800000	2.3960900000
17 H	-3.2144600000	-0.4370200000	2.0405400000
19 H	-1.9332200000	0.6947200000	-1.9135000000
20 O	3.9449100000	-0.2564800000	0.4370800000
21 N	2.1926700000	1.2066300000	0.2488000000
22 O	0.2334300000	2.2639100000	-0.3128800000
23 H	2.6523100000	-0.8328200000	-1.9677400000
24 H	1.4343600000	-2.8056000000	-1.3400900000
25 H	2.7534400000	-2.6209700000	-0.1706500000
26 C	2.7035700000	2.2073000000	1.1646600000
27 H	2.0157800000	3.0598800000	1.1441300000
28 H	3.7062000000	2.5258700000	0.8504100000
29 H	2.7562900000	1.7980300000	2.1828300000

Table S2. Energy, thermodynamic quantities, and corresponding xyz coordinates (in Angstroms; calculated with ωB97XD density functional and aug-cc-pVDZ basis set) of 4-chlorostyrene N-methylmaleimide double-Diels−Alder product.

Energy = -1566.653047 hartrees

Total enthalpy = 222.79 kcal/mol
Total entropy = 148.32 cal/mol×K

ATOM		X	Y	Z
1	C	0.5466128000	-1.6051136000	0.0217337000
2	C	-0.7062248000	-1.4172965000	0.8432965000
3	C	-0.6648391000	-0.0023809000	1.3825733000
4	C	-0.5566701000	0.9179639000	0.4211115000
5	C	-0.5080478000	0.3988649000	-0.9996830000
6	C	0.6313130000	-0.6525956000	-1.1533339000
7	C	1.5036844000	-2.5031462000	0.2579686000
8	C	2.7281624000	-2.5185714000	-0.6141577000
9	C	3.1653124000	-1.0817411000	-0.9177783000
10	C	3.7082818000	-0.3949912000	0.3283377000
11	C	2.0683022000	-0.1196371000	-1.4180317000
12	C	2.3792487000	1.1791246000	-0.6963644000
13	H	2.1583109000	0.0843823000	-2.4921935000
14	H	0.3849592000	-1.2343347000	-2.0572913000
15	H	1.4160526000	-3.2066313000	1.0896629000
16	H	-0.7887712000	-2.1639012000	1.6408812000
17	H	-0.7517643000	0.2202611000	2.4455918000
19	H	-0.4246184000	1.2029529000	-1.7378043000
20	O	4.4593571000	-0.8589364000	1.1607509000
21	N	3.2368582000	0.9048282000	0.3554101000
22	O	1.9688634000	2.2856316000	-0.9772904000
23	H	3.9961824000	-1.0925793000	-1.6408073000
24	H	2.5298298000	-3.0416549000	-1.5646684000
25	H	3.5582284000	-3.0452435000	-0.1239506000
26	C	-1.9117803000	-1.5222983000	-0.1376007000
27	C	-1.8188244000	-0.4120533000	-1.1997668000
28	C	-3.2304621000	-1.2730191000	0.5663534000
29	C	-3.0678078000	0.4265270000	-0.9945404000
30	N	-3.8234426000	-0.1511224000	0.0131477000
31	O	-3.3756806000	1.4335462000	-1.5960699000
32	O	-3.7136576000	-1.9133994000	1.4762896000
33	H	-1.8444923000	-0.8107435000	-2.2236219000
34	H	-1.9339089000	-2.5288015000	-0.5763874000
35	C	-5.0930591000	0.3720039000	0.4775870000
36	C	3.6594299000	1.8858014000	1.3350564000
37	H	-5.3591261000	1.2240875000	-0.1576071000
38	H	-5.0081422000	0.6996874000	1.5226276000
39	H	-5.8661736000	-0.4041725000	0.4040838000
40	H	3.2007236000	2.8439985000	1.0664809000
41	H	4.7534601000	1.9813073000	1.3280254000
42	H	3.3284100000	1.5824027000	2.3374517000

Table S3. Energy, thermodynamic quantities, and corresponding xyz coordinates (in Angstroms; calculated with ωB97XD density functional and aug-cc-pVDZ basis set) of 4-chlorostyrene N-methylmaleimide ene product.

Energy = -1566.687236 hartrees
Total enthalpy = 222.38 kcal/mol
Total entropy = 156.05 cal/mol×K

ATOM		X	Y	Z
1	C	-0.0481451000	-0.4906799000	0.1997029000
2	C	-1.0529171000	-1.3965560000	-0.1551951000
3	C	-0.8164455000	-2.7672901000	-0.2303957000
4	C	0.4565239000	-3.2427358000	0.0569279000
5	C	1.4821049000	-2.3744548000	0.4100164000
6	C	1.2312201000	-1.0031917000	0.4838804000
7	C	-0.2532999000	1.0137638000	0.3068950000
8	C	0.4925843000	1.5221013000	1.5493139000
9	C	1.9973065000	1.2764795000	1.4408551000
10	C	2.6490914000	2.3068775000	0.5318177000
11	C	2.3797880000	-0.0785852000	0.8178640000
12	C	3.1684127000	0.2809411000	-0.4430484000
13	H	3.0894759000	-0.6165814000	1.4630606000
14	H	0.2045099000	1.4768639000	-0.5863621000
15	H	-2.0621862000	-1.0581594000	-0.3841361000
16	H	-1.6200151000	-3.4512850000	-0.5028510000
17	H	2.4776090000	-2.7607541000	0.6307816000
18	O	2.6158426000	3.5133560000	0.6531320000
19	N	3.2972034000	1.6564041000	-0.5016392000
20	O	3.6238534000	-0.4910127000	-1.2578239000
21	H	2.4556458000	1.3941861000	2.4325415000
22	H	0.1008843000	1.0105777000	2.4420081000
23	H	0.3206840000	2.6000296000	1.6844876000
25	C	-1.7028465000	1.5141729000	0.3262969000
26	C	-2.6382285000	0.9946821000	1.4232856000
27	C	-2.4660873000	1.2725890000	-0.9658524000
28	C	-3.9544382000	0.7346392000	0.7226432000
29	N	-3.7554934000	0.8865025000	-0.6450336000
30	O	-5.0131452000	0.4275245000	1.2264919000
31	O	-2.0597458000	1.4130197000	-2.1004226000
32	H	-2.7904480000	1.7041279000	2.2462571000
33	H	-1.6434854000	2.6128660000	0.3992715000
34	C	-4.7784987000	0.6474148000	-1.6440541000
35	C	4.0110880000	2.3604773000	-1.5495828000
36	H	-5.6940513000	0.3549823000	-1.1179750000
37	H	-4.4622712000	-0.1591104000	-2.3194864000
38	H	-4.9597067000	1.5596607000	-2.2279991000
39	H	4.4374159000	1.6120929000	-2.2265307000
40	H	4.8132122000	2.9686008000	-1.1109094000
41	H	3.3217759000	3.0127677000	-2.1023093000
42	H	-2.2958580000	0.0460027000	1.8648412000

Table S4. Energy, thermodynamic quantities, and corresponding xyz coordinates (in Angstroms; calculated with ωB97XD density functional and aug-cc-pVDZ basis set) of 4-chlorostyrene.

Energy = -769.181949 hartrees
Total enthalpy = 83.71 kcal/mol
Total entropy = 87.63 cal/mol×K

ATOM		X	Y	Z
1	C	1.4440700000	0.2419400000	0.0014700000
2	C	0.5024900000	1.2764600000	0.0004800000
3	C	-0.8724400000	1.0313900000	-0.0003100000
4	C	-1.3348900000	-0.2875800000	0.0001700000
5	C	-0.4042200000	-1.3403700000	0.0014800000
6	C	0.9557500000	-1.0788600000	0.0021200000
7	C	2.8821200000	0.5722000000	0.0019300000
8	C	3.9082400000	-0.2872900000	-0.0040900000
9	H	3.1027600000	1.6456500000	0.0076800000
10	H	3.7699900000	-1.3719600000	-0.0107500000
11	H	4.9371100000	0.0785500000	-0.0028700000
12	H	0.8500300000	2.3129300000	0.0001600000
13	H	-0.7776200000	-2.3660300000	0.0021900000
14	H	1.6499100000	-1.9215600000	0.0035500000
15	H	-1.5635300000	1.8734700000	-0.0012300000

Table S5. Energy, thermodynamic quantities, and corresponding xyz coordinates (in Angstroms; calculated with ωB97XD density functional and aug-cc-pVDZ basis set) of 4-chlorostyrene N-methylmaleimide first transition state.

Energy = -1167.839684 hartrees
Total enthalpy = 150.08 kcal/mol
Total entropy = 121.78 cal/mol×K

ATOM		X	Y	Z
2	C	2.5887700000	0.2382000000	0.1499000000
3	C	2.0472500000	0.0555800000	1.4423600000
4	C	1.9427500000	0.9639100000	-0.8259500000
5	C	0.8238400000	0.6081000000	1.7288700000
6	C	-2.0993200000	2.1112900000	-0.0638400000
7	C	-1.2404000000	1.7218900000	0.9808400000
8	C	0.0972700000	1.3395100000	0.7429400000
9	C	0.6776100000	1.5027300000	-0.5409600000
10	H	2.5937900000	-0.5192000000	2.1875300000
11	H	2.3975100000	1.1134600000	-1.8027900000
12	H	0.3796600000	0.4632900000	2.7137900000
13	H	-3.1155900000	2.3840700000	0.2226200000
14	H	-1.7010100000	2.7272000000	-0.8707200000
15	H	-1.6681300000	1.4883200000	1.9569400000
16	H	0.2337200000	2.1781300000	-1.2654400000
17	O	0.2272500000	-1.8219000000	-1.1969300000
18	N	-1.6374900000	-1.2839300000	0.0668200000
19	O	-3.7471400000	-0.5137700000	0.6118600000
20	C	-0.7263300000	-1.0760700000	-0.9931800000
21	C	-2.7186400000	-0.4451900000	-0.0405700000

22	C	-2.3589800000	0.5765800000	-1.0975900000
23	C	-1.1825400000	0.0932100000	-1.7110200000
24	H	-3.1775400000	0.9899700000	-1.6857500000
25	H	-0.7713000000	0.4004400000	-2.6652300000
26	C	-1.4963800000	-2.3410000000	1.0432600000
27	H	-0.5543300000	-2.2828800000	1.5105800000
28	H	-1.6479800000	-3.3412800000	0.5794100000
29	H	-2.3170100000	-2.2243100000	1.8089300000

Table S6. Energy, thermodynamic quantities, and corresponding xyz coordinates (in Angstroms; calculated with ωB97XD density functional and aug-cc-pVDZ basis set) of 4-chlorostyrene N-methylmaleimide second transition state for Diels−Alder mechanism.

Energy = -1566.567565 hartrees
Total enthalpy = 219.33 kcal/mol
Total entropy = 154.65 cal/mol×K

ATOM		X	Y	Z
1	C	-0.7095116000	1.3446348000	0.1841909000
2	C	0.2822585000	1.0397544000	1.2122002000
3	C	0.7644182000	-0.2567599000	1.3383271000
4	C	0.7873984000	-1.0572309000	0.1928331000
5	C	0.4271090000	-0.5188127000	-1.0386419000
6	C	-0.6930636000	0.4983601000	-1.0689308000
7	C	-1.6736291000	2.2711158000	0.3118277000
8	C	-2.7318059000	2.3347676000	-0.7511668000
9	C	-3.2212272000	0.9062796000	-1.0596354000
10	C	-3.9645920000	0.3406365000	0.1418023000
11	C	-2.1058771000	-0.1210965000	-1.3325215000
12	C	-2.4286926000	-1.2886853000	-0.4144176000
13	H	-2.1246268000	-0.4958697000	-2.3643402000
14	H	-0.5363442000	1.1598806000	-1.9359173000
15	H	-1.7387607000	2.9025004000	1.2007748000
16	H	0.3621243000	1.7229738000	2.0607332000
17	H	1.2600573000	-0.5838632000	2.2526403000
18	H	0.5632554000	-1.1251106000	-1.9361654000
19	O	-4.8625909000	0.8671227000	0.7652069000
20	N	-3.4604366000	-0.9130677000	0.4291261000
21	O	-1.8985742000	-2.3796760000	-0.4009904000
22	H	-3.9340979000	0.9324183000	-1.8968812000
23	H	-2.3338361000	2.7772036000	-1.6806362000
24	H	-3.5858069000	2.9476681000	-0.4359159000
26	C	-3.9856951000	-1.7544872000	1.4864695000
27	H	-3.4484727000	-2.7085660000	1.4502373000
28	H	-5.0600822000	-1.9236158000	1.3353853000
29	H	-3.8288230000	-1.2745532000	2.4619621000
30	C	2.0058805000	1.8218773000	-0.2153428000
31	C	2.0185019000	0.9384412000	-1.2887703000
32	C	3.1055924000	1.4445479000	0.7023947000
33	C	3.1819313000	0.0177284000	-1.0964356000

34	N	3.6879429000	0.2933381000	0.1666388000
35	O	3.6192855000	-0.8197654000	-1.8594292000
36	O	3.4864106000	1.9829724000	1.7241543000
37	H	1.6695781000	1.1621835000	-2.2951451000
38	H	1.5567148000	2.8107169000	-0.1894058000
39	C	4.7794850000	-0.4187854000	0.7936994000
40	H	5.1214317000	-1.1924555000	0.0964440000
41	H	4.4415759000	-0.8911322000	1.7268624000
42	H	5.6066336000	0.2690373000	1.0166438000

Table S7. Energy, thermodynamic quantities, and corresponding xyz coordinates (in Angstroms; calculated with ωB97XD density functional and aug-cc-pVDZ basis set) of 4-chlorostyrene N-methylmaleimide second transition state for ene mechanism.

Energy = -1566.570777 hartrees
Total enthalpy = 218.14 kcal/mol
Total entropy = 153.49 cal/mol×K

ATOM		X	Y	Z
1	C	0.1193909000	0.5172886000	-0.7163746000
2	C	-0.4895695000	-0.1820968000	-1.8065053000
3	C	-0.9206890000	-1.4655443000	-1.6614942000
4	C	-0.7886080000	-2.1228387000	-0.3911071000
5	C	-0.1825938000	-1.5451636000	0.6695015000
6	C	0.2906520000	-0.1549291000	0.5785099000
7	C	0.3107000000	1.9000199000	-0.7101950000
8	C	1.2987870000	2.5034605000	0.2723275000
9	C	2.2673131000	1.4574130000	0.8202132000
10	C	3.3166124000	1.0398384000	-0.1989852000
11	C	1.6478443000	0.1312026000	1.2812164000
12	C	2.6654992000	-0.9142022000	0.8311929000
13	H	1.5234223000	0.0617989000	2.3686549000
14	H	0.1436526000	2.4377693000	-1.6466826000
15	H	-0.5998783000	0.3298424000	-2.7636103000
16	H	-1.3705797000	-2.0036347000	-2.4962251000
17	H	-0.0482391000	-2.0832326000	1.6074113000
18	O	3.9121047000	1.7508148000	-0.9796325000
19	N	3.5321329000	-0.3204295000	-0.0688127000
20	O	2.7082918000	-2.0805004000	1.1590946000
21	H	2.8330653000	1.9016144000	1.6542121000
22	H	0.7994242000	2.9856075000	1.1236923000
23	H	1.8792675000	3.2877603000	-0.2338497000
25	C	4.5544558000	-1.0361740000	-0.8072932000
26	H	4.4206277000	-2.1066872000	-0.6166129000
27	H	5.5539927000	-0.7238130000	-0.4751412000
28	H	4.4469979000	-0.8283675000	-1.8797021000
29	H	-0.4764691000	0.5156422000	1.2384129000
30	C	-1.5086266000	2.4173475000	0.2737961000
31	C	-1.5892766000	1.7465352000	1.4941389000
32	C	-2.5298434000	1.7991394000	-0.6342159000

33	C	-2.6190999000	0.6935469000	1.3784984000
34	N	-3.0843695000	0.7374859000	0.0590911000
35	O	-3.0031484000	-0.1068064000	2.2102417000
36	O	-2.8232663000	2.1133085000	-1.7703577000
37	H	-1.2380908000	2.0976274000	2.4626789000
38	H	-1.2497642000	3.4666346000	0.1457337000
39	C	-4.0249966000	-0.2007234000	-0.5073822000
40	H	-4.4025955000	-0.8293937000	0.3074101000
41	H	-3.5302600000	-0.8352181000	-1.2595528000
42	H	-4.8589366000	0.3347259000	-0.9803302000

Table S8. Energy, thermodynamic quantities, and corresponding xyz coordinates (in Angstroms; calculated with ωB97XD density functional and aug-cc-pVDZ basis set) of methyl 4-vinylbenzoate N-methylmaleimide intermediate.

Energy = -936.142573 hartrees
Total enthalpy = 188.01 kcal/mol
Total entropy = 134.75 cal/mol×K

ATOM		X	Y	Z
1	C	0.4146300000	-1.6225200000	0.3591300000
2	C	-0.6629600000	-1.7254300000	1.3349900000
3	C	-1.8669000000	-1.1597900000	1.1235200000
4	C	-2.1349700000	-0.3969900000	-0.1031000000
5	C	-1.1861600000	-0.2504600000	-1.0463800000
6	C	0.1632900000	-0.8870000000	-0.9430600000
7	C	1.6350200000	-2.1591000000	0.5460500000
8	C	2.6769800000	-1.9894300000	-0.5196500000
9	C	2.7131300000	-0.5280000000	-1.0137200000
10	C	3.3655800000	0.3705000000	0.0207900000
11	C	1.3260400000	0.1006300000	-1.2448800000
12	C	1.2995000000	1.3187100000	-0.3362100000
13	H	1.2157600000	0.4662200000	-2.2731400000
14	H	0.2175600000	-1.6227800000	-1.7672400000
15	H	1.8678800000	-2.7117700000	1.4567000000
16	H	-0.4638600000	-2.2755400000	2.2557800000
17	H	-2.6552500000	-1.2523600000	1.8670000000
18	H	-1.4005500000	0.3262500000	-1.9458800000
19	O	4.4709600000	0.2416100000	0.5128800000
20	N	2.5016600000	1.3980300000	0.3403400000
21	O	0.3906200000	2.1168900000	-0.2124300000
22	H	3.3253900000	-0.4692000000	-1.9219500000
23	H	2.4377400000	-2.6275500000	-1.3862200000
24	H	3.6729400000	-2.2799200000	-0.1690900000
25	C	2.8310100000	2.4341300000	1.3020100000
26	H	1.9641900000	3.1309400000	1.3106600000
27	H	3.6662100000	2.9946500000	1.0200600000
28	H	3.0133100000	1.9813000000	2.3252200000
29	C	-3.4590000000	0.2465000000	-0.3336700000
30	O	-4.3107000000	0.0265000000	0.6722700000

31	O	-3.7504400000	0.9010600000	-1.3182700000
32	C	-5.6200700000	0.5984400000	0.5368600000
33	H	-6.1541000000	0.3220000000	1.4488500000
34	H	-6.1239800000	0.1826600000	-0.3432000000
35	H	-5.5508600000	1.6884800000	0.4479200000

Table S9. Energy, thermodynamic quantities, and corresponding xyz coordinates (in Angstroms; calculated with ωB97XD density functional and aug-cc-pVDZ basis set) of methyl 4-vinylbenzoate N-methylmaleimide double-Diels−Alder product.

Energy = -1334.896182 hartrees
Total enthalpy = 257.35 kcal/mol
Total entropy = 167.41 cal/mol×K

ATOM		X	Y	Z
1	C	0.6623100000	-1.8442600000	0.2749400000
2	C	-0.5642300000	-1.4982600000	1.0886200000
3	C	-0.5408600000	0.0036200000	1.2421600000
4	C	-0.5215200000	0.6762400000	0.0805500000
5	C	-0.5120900000	-0.1943100000	-1.1569700000
6	C	0.6878100000	-1.1796000000	-1.0869200000
7	C	1.6635000000	-2.6335800000	0.6677200000
8	C	2.8630700000	-2.7763700000	-0.2289600000
9	C	3.2391000000	-1.3977700000	-0.7872900000
10	C	3.7276700000	-0.4765900000	0.3244900000
11	C	2.0910800000	-0.6075000000	-1.4489500000
12	C	2.3053500000	0.8082500000	-0.9467000000
13	H	2.1770600000	-0.5844800000	-2.5424800000
14	H	0.5021900000	-1.9623100000	-1.8410700000
15	H	1.6415500000	-3.1314300000	1.6401700000
16	H	-0.5849200000	-2.0250400000	2.0486200000
17	H	-0.5689600000	0.4823600000	2.2209600000
18	H	-0.5008600000	0.4009000000	-2.0759600000
19	O	4.4999400000	-0.7516600000	1.2182500000
20	N	3.1575000000	0.7716900000	0.1379800000
21	O	1.8267400000	1.8325700000	-1.3920400000
22	H	4.0761600000	-1.4962800000	-1.4960500000
23	H	2.6534200000	-3.4560600000	-1.0718600000
24	H	3.7218000000	-3.1907100000	0.3155600000
25	C	-1.8069800000	-1.8662200000	0.2282500000
26	C	-1.7740300000	-1.0957700000	-1.1025900000
27	C	-3.1026500000	-1.4362400000	0.8894200000
28	C	-3.0636400000	-0.2963400000	-1.1229200000
29	N	-3.7579000000	-0.5534900000	0.0495500000
30	O	-3.4484900000	0.4497100000	-1.9978300000
31	O	-3.5227900000	-1.7766300000	1.9756500000
32	H	-1.7795100000	-1.7654300000	-1.9739100000
33	H	-1.8360000000	-2.9550300000	0.0857000000
34	C	-5.0284500000	0.0557900000	0.3900300000
35	C	3.4414800000	1.9397900000	0.9483600000

36	H	-5.1766400000	1.0697500000	0.0023700000
37	H	-5.1072200000	0.0584800000	1.4852900000
38	H	-5.7906700000	-0.6161300000	-0.0271400000
39	H	2.5007400000	2.3438900000	1.3459200000
40	H	3.9419000000	2.7099600000	0.3459000000
41	H	4.0957200000	1.6271300000	1.7696700000
42	C	-0.6336600000	2.1500700000	-0.0209700000
43	O	-1.0825900000	2.7318000000	-0.9857600000
44	O	-0.2165400000	2.7686200000	1.0898300000
45	C	-0.3317200000	4.1955000000	1.0896200000
46	H	-1.3751200000	4.4998000000	0.9287600000
47	H	0.3024200000	4.6273400000	0.3029300000
48	H	0.0125500000	4.5192200000	2.0771800000

Table S10. Energy, thermodynamic quantities, and corresponding xyz coordinates (in Angstroms; calculated with ωB97XD density functional and aug-cc-pVDZ basis set) of methyl 4-vinylbenzoate N-methylmaleimide ene product.

Energy = -1334.926872 hartrees
Total enthalpy = 257.41 kcal/mol
Total entropy = 171.33 cal/mol×K

ATOM		X	Y	Z
1	C	0.0792700000	-0.1782700000	-0.2441200000
2	C	0.1320100000	-1.5367300000	0.0895300000
3	C	-1.0229900000	-2.3051700000	0.1806300000
4	C	-2.2685400000	-1.7284700000	-0.0699700000
5	C	-2.3368600000	-0.3774300000	-0.4148100000
6	C	-1.1803700000	0.3945600000	-0.5054700000
7	C	1.2955500000	0.7293600000	-0.3405600000
8	C	1.1389800000	1.6258200000	-1.5779200000
9	C	-0.0753300000	2.5433100000	-1.4377000000
10	C	0.2322800000	3.7000200000	-0.4982300000
11	C	-1.3145000000	1.8671200000	-0.8222200000
12	C	-1.6004100000	2.6663900000	0.4508700000
13	H	-2.1970600000	2.0100100000	-1.4624200000
14	H	1.2834300000	1.3763900000	0.5561400000
15	H	1.0813700000	-2.0307700000	0.2904400000
16	H	-0.9622700000	-3.3608800000	0.4460700000
17	H	-3.3041800000	0.0837300000	-0.6136800000
18	O	1.1362300000	4.5027000000	-0.5969000000
19	N	-0.6878600000	3.7018200000	0.5336700000
20	O	-2.4768900000	2.4513600000	1.2588200000
21	H	-0.3146000000	2.9837900000	-2.4154700000
22	H	1.0262100000	0.9983500000	-2.4751100000
23	H	2.0360100000	2.2466200000	-1.7176500000
24	C	2.6755000000	0.0641000000	-0.3303300000
25	C	3.0153000000	-0.9450200000	-1.4317200000
26	C	2.9975500000	-0.6663500000	0.9630500000
27	C	3.7304000000	-2.0742200000	-0.7203300000

28	N	3.6462400000	-1.8471800000	0.6488500000
29	O	4.2761200000	-3.0348300000	-1.2189600000
30	O	2.7675000000	-0.2963300000	2.0954600000
31	H	3.6599300000	-0.5400900000	-2.2217300000
32	H	3.4088000000	0.8870900000	-0.3631700000
33	C	4.1494500000	-2.7609500000	1.6553200000
34	C	-0.6663700000	4.6761900000	1.6082400000
35	H	4.2279000000	-3.7244900000	1.1398100000
36	H	3.5344700000	-2.8702000000	2.5590000000
37	H	5.1496300000	-2.4073200000	1.9391300000
38	H	-1.4017100000	4.3628000000	2.3574700000
39	H	-0.9278400000	5.6721100000	1.2255000000
40	H	0.3357100000	4.7104300000	2.0549300000
41	H	2.1216600000	-1.3663400000	-1.9184100000
42	C	-3.4856800000	-2.5807800000	0.0492700000
43	O	-3.4645800000	-3.7595300000	0.3390200000
44	O	-4.6105500000	-1.9046400000	-0.1952200000
45	C	-5.8283000000	-2.6520900000	-0.0965800000
46	H	-6.6300600000	-1.9377900000	-0.3093900000
47	H	-5.9424500000	-3.0687600000	0.9134200000
48	H	-5.8375400000	-3.4678700000	-0.8327000000

Table S11. Energy, thermodynamic quantities, and corresponding xyz coordinates (in Angstroms; calculated with ωB97XD density functional and aug-cc-pVDZ basis set) of methyl 4-vinylbenzoate.

Energy = -537.421449 hartrees
Total enthalpy = 118.92 kcal/mol
Total entropy = 104.05 cal/mol×K

ATOM		X	Y	Z
1	C	1.4292700000	0.2294100000	0.0014700000
2	C	0.6031700000	1.3626200000	0.0000800000
3	C	-0.7860700000	1.2538500000	-0.0008100000
4	C	-1.3581100000	-0.0139700000	-0.0000400000
5	C	-0.5681900000	-1.1629700000	0.0016600000
6	C	0.8163300000	-1.0343100000	0.0024400000
7	C	2.8952400000	0.4128000000	0.0020200000
8	C	3.8216400000	-0.5519100000	-0.0038800000
9	H	3.2254200000	1.4570500000	0.0075800000
10	H	3.5688100000	-1.6154900000	-0.0104100000
11	H	4.8835800000	-0.2979200000	-0.0027600000
12	H	1.0428800000	2.3526900000	-0.0005700000
13	H	-1.0322900000	-2.1496400000	0.0025600000
14	H	1.4222300000	-1.9418000000	0.0042500000
15	H	-1.4147100000	2.1447000000	-0.0020200000
16	C	-2.8414400000	-0.1422700000	-0.0007800000
17	O	-3.3613100000	-1.2511600000	-0.0000700000
18	O	-3.6176200000	0.9740700000	-0.0021000000
19	C	-4.9963600000	0.9393200000	-0.0025300000
20	H	-5.3861300000	1.9778800000	-0.0036300000

21	H	-5.3671900000	0.4196100000	0.9068700000
22	H	-5.3666100000	0.4178700000	-0.9111700000

Table S12. Energy, thermodynamic quantities, and corresponding xyz coordinates (in Angstroms; calculated with ωB97XD density functional and aug-cc-pVDZ basis set) of methyl 4-vinylbenzoate N-methylmaleimide first transition state.

Energy = -936.078789 hartrees
Total enthalpy = 185.54 kcal/mol
Total entropy = 136.96 cal/mol×K

ATOM		X	Y	Z
1	C	-1.9953100000	0.5802200000	-0.1521200000
2	C	-1.4947600000	0.1975900000	-1.4268200000
3	C	-1.2189200000	1.3392200000	0.7018200000
4	C	-0.2301100000	0.5643100000	-1.8052800000
5	C	2.8927300000	1.9044600000	-0.2326800000
6	C	1.9752200000	1.4962200000	-1.2193500000
7	C	0.6081200000	1.3172700000	-0.9276600000
8	C	0.0914000000	1.6963400000	0.3393900000
9	H	-2.1140300000	-0.3957400000	-2.0950600000
10	H	-1.6190900000	1.6516000000	1.6643300000
11	H	0.1658600000	0.2546300000	-2.7725900000
12	H	3.9341700000	1.9901200000	-0.5445700000
13	H	2.5928100000	2.6733300000	0.4802900000
14	H	2.3536400000	1.0854100000	-2.1564200000
15	H	0.6222900000	2.4110900000	0.9599400000
16	O	0.1039300000	-1.5388300000	1.4086400000
17	N	2.0056400000	-1.3961900000	0.0983100000
18	O	4.1884400000	-0.9613600000	-0.5228000000
19	C	1.1329100000	-0.9442400000	1.1085600000
20	C	3.1813600000	-0.6840600000	0.1053800000
21	C	2.9537700000	0.5054700000	1.0114800000
22	C	1.7263700000	0.2585400000	1.6648100000
23	H	3.8160700000	0.8892800000	1.5551700000
24	H	1.3746200000	0.7180400000	2.5809600000
25	C	1.7179800000	-2.5295300000	-0.7522600000
26	H	0.6871900000	-2.4211100000	-1.1877800000
27	H	1.7616000000	-3.4696500000	-0.1463900000
28	H	2.4250400000	-2.5824500000	-1.5237200000
29	C	-3.3682200000	0.1937300000	0.2895300000
30	O	-3.8325800000	0.4576300000	1.3825900000
31	O	-4.0374400000	-0.4759600000	-0.6496500000
32	C	-5.3718100000	-0.8864200000	-0.3132200000
33	H	-5.7534100000	-1.3893700000	-1.2045900000
34	H	-5.9865800000	-0.0116100000	-0.0729600000
35	H	-5.3519800000	-1.5766300000	0.5378500000

Table S13. Energy, thermodynamic quantities, and corresponding xyz coordinates (in Angstroms; calculated with ωB97XD density functional and aug-cc-pVDZ basis set) of methyl 4-vinylbenzoate N-methylmaleimide second transition state for Diels−Alder mechanism.

Energy = -1334.809663 hartrees
Total enthalpy = 254.60 kcal/mol
Total entropy = 167.38 cal/mol×K

ATOM		X	Y	Z
1	C	-1.0567900000	1.4256600000	0.3965500000
2	C	-0.0029500000	1.1039600000	1.3535800000
3	C	0.6770200000	-0.0997600000	1.2514100000
4	C	0.7880500000	-0.7175400000	-0.0048600000
5	C	0.2714900000	-0.0533200000	-1.1182800000
6	C	-0.9683300000	0.7906300000	-0.9694200000
7	C	-2.1286800000	2.1896000000	0.6609900000
8	C	-3.1887400000	2.2914100000	-0.3978400000
9	C	-3.5230100000	0.8834800000	-0.9405200000
10	C	-4.2504200000	0.0725300000	0.1164400000
11	C	-2.2903900000	0.0257400000	-1.2959600000
12	C	-2.4777000000	-1.2582700000	-0.5063300000
13	H	-2.2736700000	-0.2460600000	-2.3575700000
14	H	-0.9451900000	1.5863700000	-1.7278300000
15	H	-2.2622600000	2.6704700000	1.6298200000
16	H	0.0094700000	1.6511000000	2.2958800000
17	H	1.2451000000	-0.4775300000	2.0978500000
18	H	0.4528400000	-0.4845100000	-2.1019500000
19	O	-5.2614400000	0.3858600000	0.7168500000
20	N	-3.5870200000	-1.1215000000	0.3085100000
21	O	-1.8012600000	-2.2659400000	-0.5736900000
22	H	-4.1955300000	0.9748000000	-1.8022100000
23	H	-2.8363000000	2.9049800000	-1.2420000000
24	H	-4.1014300000	2.7593600000	-0.0150500000
25	C	1.6119500000	-1.9372600000	-0.2137800000
26	C	-4.0547300000	-2.1412500000	1.2290600000
27	H	-3.3312100000	-2.9600400000	1.2210200000
28	H	-5.0378600000	-2.5080500000	0.9136100000
29	H	-4.1284800000	-1.7183100000	2.2365200000
30	O	2.1095500000	-2.4186000000	0.9272900000
31	O	1.8024700000	-2.4558500000	-1.2976100000
32	C	2.9184700000	-3.5991500000	0.8188600000
33	H	3.1982400000	-3.8546100000	1.8432700000
34	H	3.8114400000	-3.3904200000	0.2186600000
35	H	2.3443900000	-4.4135000000	0.3630200000
36	C	1.5599200000	2.3041800000	0.0261200000
37	C	1.6365400000	1.6268100000	-1.1861200000
38	C	2.7394500000	1.9262200000	0.8361800000
39	C	2.9224300000	0.8713400000	-1.1862900000
40	N	3.4359400000	0.9707000000	0.0998700000
41	O	3.4517300000	0.2743200000	-2.1050400000
42	O	3.0951800000	2.3378300000	1.9281400000

43	H	1.2086400000	1.9640700000	-2.1249900000
44	H	0.9854100000	3.1978400000	0.2399800000
45	C	4.6383100000	0.3138600000	0.5692900000
46	H	4.9943000000	-0.3241200000	-0.2427300000
47	H	4.4261400000	-0.2497800000	1.4723800000
48	H	5.4125100000	1.0551900000	0.8316900000

Table S14. Energy, thermodynamic quantities, and corresponding xyz coordinates (in Angstroms; calculated with ωB97XD density functional and aug-cc-pVDZ basis set) of methyl 4-vinylbenzoate N-methylmaleimide second transition state for ene mechanism.

Energy = -1334.810367 hartrees
Total enthalpy = 253.07 kcal/mol
Total entropy = 170.87 cal/mol×K

ATOM		X	Y	Z
1	C	-0.5757510000	0.6603344000	0.7487523000
2	C	0.3483305000	0.2398633000	1.7621431000
3	C	1.2546892000	-0.7452439000	1.5210187000
4	C	1.3131150000	-1.3885520000	0.2308343000
5	C	0.4289319000	-1.0607373000	-0.7469966000
6	C	-0.5445157000	0.0181054000	-0.5710851000
7	C	-1.3083605000	1.8413668000	0.8338750000
8	C	-2.5029620000	2.0463586000	-0.0771413000
9	C	-3.0013537000	0.7332012000	-0.6743079000
10	C	-3.7513771000	-0.1282077000	0.3316434000
11	C	-1.9283737000	-0.2097738000	-1.2357937000
12	C	-2.4318954000	-1.5984182000	-0.8514564000
13	H	-1.8293091000	-0.1591834000	-2.3269769000
14	H	-1.2998440000	2.3823230000	1.7832328000
15	H	0.3103128000	0.7313471000	2.7356015000
16	H	1.9463557000	-1.0619317000	2.3009226000
17	H	0.4584120000	-1.5902618000	-1.6995805000
18	O	-4.5405648000	0.2383201000	1.1754900000
19	N	-3.4260062000	-1.4526199000	0.0982066000
20	O	-2.0230055000	-2.6628003000	-1.2637647000
21	H	-3.7340817000	0.9591827000	-1.4648587000
22	H	-2.2837243000	2.7370787000	-0.9032517000
23	H	-3.3207213000	2.5037424000	0.4980340000
24	C	-4.0674024000	-2.5582909000	0.7832793000
25	H	-3.7218206000	-3.4874930000	0.3171523000
26	H	-5.1573654000	-2.4714600000	0.6866000000
27	H	-3.7957410000	-2.5539667000	1.8477407000
28	H	-0.1100847000	0.9674944000	-1.2166258000
29	C	2.3282458000	-2.4389818000	-0.0732189000
30	O	2.3621927000	-3.0824236000	-1.1015905000
31	O	3.2141738000	-2.5842598000	0.9145641000
32	C	4.2280020000	-3.5756447000	0.7090571000
33	H	4.8082907000	-3.3494442000	-0.1958196000
34	H	4.8679872000	-3.5312488000	1.5958543000

35	H	3.7749286000	-4.5720961000	0.6132131000
36	C	0.1443543000	3.0754231000	-0.2294364000
37	C	0.4149684000	2.4958889000	-1.4668487000
38	C	1.3656022000	2.9000614000	0.6217000000
39	C	1.7914506000	1.9527301000	-1.4276079000
40	N	2.2675905000	2.1634805000	-0.1311466000
41	O	2.4254175000	1.3898977000	-2.2994131000
42	O	1.5585848000	3.2886446000	1.7561724000
43	H	-0.0895239000	2.6993357000	-2.4098141000
44	H	-0.5148865000	3.9218947000	-0.0500810000
45	C	3.5358469000	1.6679445000	0.3514412000
46	H	4.2835726000	1.7444444000	-0.4475660000
47	H	3.4509290000	0.6150487000	0.6647005000
48	H	3.8415608000	2.2755762000	1.2113285000

Table S15. Energy, thermodynamic quantities, and corresponding xyz coordinates (in Angstroms; calculated with ωB97XD density functional and aug-cc-pVDZ basis set) of 4-fluorostyrene N-methylmaleimide intermediate.

Energy = -807.522040 hartrees
Total enthalpy = 153.54 kcal/mol
Total entropy = 118.03 cal/mol×K

ATOM		X	Y	Z
1	C	-0.7330500000	-1.3733600000	0.4174900000
2	C	-1.7570300000	-1.2024300000	1.4403200000
3	C	-2.8268500000	-0.4104300000	1.2346200000
4	C	-2.9641600000	0.2958700000	-0.0305300000
5	C	-2.0855100000	0.2195600000	-1.0318000000
6	C	-0.8757700000	-0.6638600000	-0.9201800000
7	C	0.3596100000	-2.1414000000	0.5969900000
8	C	1.3770700000	-2.2198800000	-0.5002900000
9	C	1.6956500000	-0.8091600000	-1.0356400000
10	C	2.5167600000	-0.0331100000	-0.0194000000
11	C	0.4528900000	0.0673100000	-1.2789500000
12	C	0.6837300000	1.3092500000	-0.4322900000
13	H	0.3892200000	0.3986300000	-2.3252300000
14	H	-0.9669600000	-1.4420300000	-1.7029800000
15	H	0.5147100000	-2.6848400000	1.5323400000
16	H	-1.6271900000	-1.7293400000	2.3884100000
17	H	-3.5990500000	-0.2691600000	1.9922000000
18	H	-2.2449900000	0.7827700000	-1.9523800000
19	O	3.5659600000	-0.3710600000	0.4889900000
20	N	1.8754700000	1.1580100000	0.2583400000
21	O	-0.0256200000	2.2901900000	-0.3550100000
22	H	2.3041200000	-0.8984200000	-1.9467600000
23	H	0.9834000000	-2.8201500000	-1.3401100000
24	H	2.3054900000	-2.7003900000	-0.1654200000
25	C	2.4084400000	2.1452300000	1.1762700000
26	H	1.9554200000	3.0709300000	0.8045800000

27	H	3.5028800000	2.2177600000	1.1297700000
28	H	2.1020200000	1.9736000000	2.2173800000
29	F	-4.0706200000	1.0682000000	-0.1411600000

Table S16. Energy, thermodynamic quantities, and corresponding xyz coordinates (in Angstroms; calculated with ωB97XD density functional and aug-cc-pVDZ basis set) of 4-fluorostyrene N-methylmaleimide double-Diels−Alder product.

Energy = -1206.271264 hartrees
Total enthalpy = 223.32 kcal/mol
Total entropy = 145.63 cal/mol×K

ATOM		X	Y	Z
1	C	0.4996841000	-1.4467513000	-0.0709930000
2	C	-0.7295915000	-1.3009033000	0.7951923000
3	C	-0.6782507000	0.0822178000	1.4175941000
4	C	-0.5998585000	1.0424767000	0.4979718000
5	C	-0.5748111000	0.6142057000	-0.9442934000
6	C	0.5769008000	-0.4056616000	-1.1718113000
7	C	1.4543479000	-2.3692562000	0.0574817000
8	C	2.6418365000	-2.3177954000	-0.8640105000
9	C	3.1062639000	-0.8657364000	-1.0372364000
10	C	3.7405635000	-0.3461178000	0.2453122000
11	C	2.0079875000	0.1697936000	-1.3582027000
12	C	2.3234521000	1.3368773000	-0.4385933000
13	H	2.0867446000	0.5470261000	-2.3861210000
14	H	0.3579534000	-0.9159963000	-2.1248563000
15	H	1.3931836000	-3.1392986000	0.8306690000
16	H	-0.7913729000	-2.0937597000	1.5489649000
17	H	-0.7350475000	0.2557605000	2.4914207000
18	H	-0.5165977000	1.4714277000	-1.6238600000
19	O	4.5522115000	-0.9099951000	0.9493758000
20	N	3.2634066000	0.9274524000	0.4908531000
21	O	1.8583601000	2.4562950000	-0.4938270000
22	H	3.8936996000	-0.8221747000	-1.8057871000
23	H	2.3891921000	-2.7337958000	-1.8538317000
24	H	3.4789638000	-2.9096687000	-0.4697214000
25	C	-1.9563577000	-1.3576016000	-0.1637713000
26	C	-1.8802180000	-0.1974423000	-1.1732868000
27	C	-3.2593785000	-1.1358871000	0.5787321000
28	C	-3.1262691000	0.6288649000	-0.9111013000
29	N	-3.8656245000	0.0051170000	0.0800787000
30	O	-3.4391777000	1.6662651000	-1.4562768000
31	O	-3.7231056000	-1.8098165000	1.4742670000
32	H	-1.9142196000	-0.5439259000	-2.2156596000
33	H	-1.9943091000	-2.3416657000	-0.6501259000
34	C	-5.1223633000	0.5143558000	0.5928611000
35	C	3.7423373000	1.7592260000	1.5770700000
36	H	-5.4068371000	1.3795111000	-0.0162820000
37	H	-5.0089554000	0.8204574000	1.6418662000

38	H	-5.8957664000	-0.2617797000	0.5247420000
39	H	3.2261349000	2.7232792000	1.5102798000
40	H	4.8268300000	1.9090688000	1.4897263000
41	H	3.5187031000	1.2827952000	2.5410331000
42	F	-0.6307480000	2.3548420000	0.7788020000

Table S17. Energy, thermodynamic quantities, and corresponding xyz coordinates (in Angstroms; calculated with ωB97XD density functional and aug-cc-pVDZ basis set) of 4-fluorostyrene N-methylmaleimide ene product.

Energy = -1206.306013 hartrees
Total enthalpy = 222.88 kcal/mol
Total entropy = 153.19 cal/mol×K

ATOM		X	Y	Z
1	C	-0.0152300000	-0.7160100000	0.1675600000
2	C	-1.0217000000	-1.5843100000	-0.2681200000
3	C	-0.8181700000	-2.9590800000	-0.3697100000
4	C	0.4236400000	-3.4574200000	-0.0167800000
5	C	1.4533000000	-2.6414700000	0.4163000000
6	C	1.2371500000	-1.2641000000	0.4992600000
7	C	-0.2023600000	0.7878500000	0.3071500000
8	C	0.5456900000	1.2704600000	1.5590200000
9	C	2.0453000000	1.0098100000	1.4412800000
10	C	2.6933300000	1.9805600000	0.4698000000
11	C	2.4046600000	-0.3821600000	0.8927600000
12	C	3.2727800000	-0.0975400000	-0.3349700000
13	H	3.0425800000	-0.9299400000	1.5966300000
14	H	0.2566600000	1.2682800000	-0.5724600000
15	H	-2.0044300000	-1.2110300000	-0.5445600000
16	H	-1.6089800000	-3.6255700000	-0.7089000000
17	H	2.4168200000	-3.0737400000	0.6819000000
18	O	2.6417300000	3.1952700000	0.5043000000
19	N	3.3752000000	1.2705300000	-0.4971400000
20	O	3.7967300000	-0.9172000000	-1.0609700000
21	H	2.5229200000	1.1723300000	2.4150500000
22	H	0.1568700000	0.7475200000	2.4429800000
23	H	0.3808400000	2.3442400000	1.7092100000
24	C	-1.6443700000	1.3064600000	0.3541800000
25	C	-2.5784400000	0.7557300000	1.4393000000
26	C	-2.4333400000	1.1487100000	-0.9327100000
27	C	-3.9061000000	0.5626100000	0.7435700000
28	N	-3.7285800000	0.7877000000	-0.6131200000
29	O	-4.9652100000	0.2451000000	1.2492700000
30	O	-2.0422000000	1.3375800000	-2.0689400000
31	H	-2.7030900000	1.4294200000	2.2926200000
32	H	-1.5675800000	2.3971900000	0.4779400000
33	C	-4.7687300000	0.6321000000	-1.6126300000
34	C	4.1123900000	1.9123300000	-1.5704400000
35	H	-5.6944300000	0.3624700000	-1.0983500000

36	H	-4.4907400000	-0.1584700000	-2.3188900000
37	H	-4.9032700000	1.5741700000	-2.1552600000
38	H	4.5080000000	1.1254100000	-2.2322700000
39	H	4.9571200000	2.4899900000	-1.1378800000
40	H	3.4363700000	2.5681000000	-2.1421100000
41	H	-2.2530400000	-0.2184200000	1.8281100000
42	F	0.6432000000	-4.7957200000	-0.0953300000

Table S18. Energy, thermodynamic quantities, and corresponding xyz coordinates (in Angstroms; calculated with ωB97XD density functional and aug-cc-pVDZ basis set) of 4-fluorostyrene.

Energy = -408.800810 hartrees
Total enthalpy = 84.28 kcal/mol
Total entropy = 85.10 cal/mol×K

ATOM		X	Y	Z
1	C	1.4292700000	0.2294100000	0.0014700000
2	C	0.6031700000	1.3626200000	0.0000800000
3	C	-0.7860700000	1.2538500000	-0.0008100000
4	C	-1.3581100000	-0.0139700000	-0.0000400000
5	C	-0.5681900000	-1.1629700000	0.0016600000
6	C	0.8163300000	-1.0343100000	0.0024400000
7	C	2.8952400000	0.4128000000	0.0020200000
8	C	3.8216400000	-0.5519100000	-0.0038800000
9	H	3.2254200000	1.4570500000	0.0075800000
10	H	3.5688100000	-1.6154900000	-0.0104100000
11	H	4.8835800000	-0.2979200000	-0.0027600000
12	H	1.0429400000	2.3526900000	-0.0005700000
13	H	-1.0322900000	-2.1496400000	0.0025600000
14	H	1.4222300000	-1.9418000000	0.0042500000
15	H	-1.4147100000	2.1447000000	-0.0020200000
16	F	-2.7085800000	-0.1347700000	-0.0007800000

Table S19. Energy, thermodynamic quantities, and corresponding xyz coordinates (in Angstroms; calculated with ωB97XD density functional and aug-cc-pVDZ basis set) of 4-fluorostyrene N-methylmaleimide first transition state.

Energy = -807.459482 hartrees
Total enthalpy = 150.94 kcal/mol
Total entropy = 118.54 cal/mol×K

ATOM		X	Y	Z
1	C	-2.8500897000	-0.0078554000	-0.0619382000
2	C	-2.3405103000	-0.1659304000	-1.3660856000
3	C	-2.2571970000	0.7978659000	0.8801523000
4	C	-1.1794184000	0.4821656000	-1.6995829000
5	C	1.6952625000	2.1815483000	-0.0222824000
6	C	0.8227107000	1.7314197000	-1.0286057000
7	C	-0.4841650000	1.2851373000	-0.7448795000

8	C	-1.0476427000	1.4307007000	0.5526475000
9	H	-2.8705964000	-0.8038206000	-2.0739191000
10	H	-2.7120715000	0.9179385000	1.8630450000
11	H	-0.7515348000	0.3590209000	-2.6965511000
12	H	2.6933966000	2.4867116000	-0.3480397000
13	H	1.2948751000	2.8040046000	0.7823317000
14	H	1.2344899000	1.4879104000	-2.0119951000
15	H	-0.6416268000	2.1641902000	1.2461784000
16	O	-0.4476619000	-1.8068641000	1.2759034000
17	N	1.3822915000	-1.2488795000	-0.0397730000
18	O	3.4298000000	-0.4008033000	-0.6755320000
19	C	0.4753240000	-1.0436280000	1.0297646000
20	C	2.4260011000	-0.3555993000	0.0106536000
21	C	2.0442914000	0.6820298000	1.0475855000
22	C	0.9041525000	0.1687562000	1.7003243000
23	H	2.8596199000	1.1471709000	1.6059751000
24	H	0.5066024000	0.4810196000	2.6615736000
25	C	1.2862132000	-2.3387048000	-0.9824748000
26	H	0.5993779000	-3.0870006000	-0.5689291000
27	H	2.2764906000	-2.7864048000	-1.1379027000
28	H	0.8935072000	-1.9874827000	-1.9492904000
29	F	-3.9872521000	-0.6519106000	0.2456990000

Table S20. Energy, thermodynamic quantities, and corresponding xyz coordinates (in Angstroms; calculated with ωB97XD density functional and aug-cc-pVDZ basis set) of 4-fluorostyrene N-methylmaleimide second transition state for Diels−Alder mechanism.

Energy = -1206.186049 hartrees
Total enthalpy = 220.00 kcal/mol
Total entropy = 151.47 cal/mol×K

ATOM		X	Y	Z
1	C	-0.5725172000	1.2091549000	0.2018592000
2	C	0.4021200000	0.8917538000	1.2417880000
3	C	0.8648079000	-0.4084066000	1.3816623000
4	C	0.8656730000	-1.2149572000	0.2465464000
5	C	0.5148866000	-0.7006666000	-0.9945107000
6	C	-0.5808340000	0.3385732000	-1.0357894000
7	C	-1.5138456000	2.1632365000	0.3014088000
8	C	-2.5412183000	2.2530966000	-0.7893184000
9	C	-3.0862516000	0.8449863000	-1.1136488000
10	C	-3.9537752000	0.3533588000	0.0333932000
11	C	-2.0077229000	-0.2440451000	-1.2712855000
12	C	-2.3989677000	-1.3227720000	-0.2732020000
13	H	-2.0282150000	-0.7013804000	-2.2699877000
14	H	-0.4131681000	0.9795667000	-1.9166207000
15	H	-1.5748783000	2.8115531000	1.1784717000
16	H	0.5045190000	1.5912078000	2.0743058000
17	H	1.3759309000	-0.7406206000	2.2858357000
18	H	0.6213158000	-1.3521157000	-1.8644372000

19	O	-4.9005624000	0.9242434000	0.5339315000
20	N	-3.4951722000	-0.8824008000	0.4482453000
21	O	-1.8704404000	-2.4039005000	-0.1146332000
22	H	-3.7249775000	0.9004434000	-2.0065603000
23	H	-2.0948840000	2.6709035000	-1.7085437000
24	H	-3.3782628000	2.9050205000	-0.5082448000
25	F	1.4674594000	-2.4162406000	0.3114480000
26	C	-4.1258508000	-1.6390297000	1.5116982000
27	H	-3.5148610000	-2.5278539000	1.7035072000
28	H	-5.1384588000	-1.9435272000	1.2132860000
29	H	-4.1879173000	-1.0225900000	2.4179646000
30	C	2.1629550000	1.6037660000	-0.2875005000
31	C	2.1219938000	0.6460872000	-1.2997879000
32	C	3.2482749000	1.2420548000	0.6427367000
33	C	3.2568039000	-0.3035836000	-1.0589457000
34	N	3.7821215000	0.0320886000	0.1778476000
35	O	3.6489969000	-1.2082037000	-1.7689504000
36	O	3.6669286000	1.8244938000	1.6261342000
37	H	1.7926504000	0.8342684000	-2.3206233000
38	H	1.7374110000	2.6026987000	-0.3131749000
39	C	4.8547977000	-0.6800489000	0.8370531000
40	H	5.2045624000	-1.4637534000	0.1548294000
41	H	4.4958103000	-1.1401111000	1.7686330000
42	H	5.6802854000	0.0062257000	1.0688779000

Table S21. Energy, thermodynamic quantities, and corresponding xyz coordinates (in Angstroms; calculated with ωB97XD density functional and aug-cc-pVDZ basis set) of 4-fluorostyrene N-methylmaleimide second transition state for ene mechanism.

Energy = -1206.188999 hartrees
Total enthalpy = 218.78 kcal/mol
Total entropy = 150.50 cal/mol×K

ATOM		X	Y	Z
1	C	0.0044000000	0.3493600000	-0.7175600000
2	C	-0.5248800000	-0.3722200000	-1.8335500000
3	C	-0.8081500000	-1.7007100000	-1.7347700000
4	C	-0.5888100000	-2.3683700000	-0.4886600000
5	C	-0.0688800000	-1.7743600000	0.6012500000
6	C	0.2427700000	-0.3360800000	0.5619100000
7	C	0.0540800000	1.7441400000	-0.6755500000
8	C	0.9804800000	2.4156300000	0.3224500000
9	C	2.0544200000	1.4585800000	0.8326000000
10	C	3.1257800000	1.1436800000	-0.1990900000
11	C	1.5640300000	0.0734500000	1.2807600000
12	C	2.6876100000	-0.8579400000	0.8407700000
13	H	1.4337700000	-0.0111600000	2.3637400000
14	H	-0.1496100000	2.2823700000	-1.6010100000
15	H	-0.6947600000	0.1591200000	-2.7683100000
16	H	-1.2010500000	-2.2673500000	-2.5772600000

17	H	0.1217400000	-2.3427400000	1.5088900000
18	O	3.6463000000	1.9121200000	-0.9839000000
19	N	3.4785800000	-0.1857300000	-0.0720700000
20	O	2.8699600000	-2.0050400000	1.1955200000
21	H	2.5867500000	1.9360500000	1.6669200000
22	H	0.4422700000	2.8095500000	1.1920600000
23	H	1.4618300000	3.2756600000	-0.1577900000
24	C	4.5824600000	-0.7830400000	-0.8009000000
25	H	4.7142400000	-1.8048100000	-0.4368600000
26	H	5.4942700000	-0.2022700000	-0.6257800000
27	H	4.3565700000	-0.7959100000	-1.8730300000
28	H	-0.5763700000	0.2311100000	1.2272400000
29	F	-0.9094800000	-3.6883800000	-0.4528100000
30	C	-1.7927300000	2.0729600000	0.2880400000
31	C	-1.8218600000	1.3968400000	1.5083700000
32	C	-2.7464600000	1.3594000000	-0.6217600000
33	C	-2.7394700000	0.2564500000	1.3860100000
34	N	-3.1940300000	0.2455500000	0.0607700000
35	O	-3.0636700000	-0.5713500000	2.2229800000
36	O	-3.0737700000	1.6543300000	-1.7573200000
37	H	-1.4964700000	1.7686400000	2.4746400000
38	H	-1.6452600000	3.1420000000	0.1664300000
39	C	-4.0318800000	-0.7782100000	-0.5219900000
40	H	-4.2988700000	-1.4768200000	0.2634200000
41	H	-3.4921300000	-1.2942700000	-1.3180200000
42	H	-4.9737700000	-0.3499800000	-0.8616400000

Table S22. Energy, thermodynamic quantities, and corresponding xyz coordinates (in Angstroms; calculated with ωB97XD density functional and aug-cc-pVDZ basis set) of maleic anhydride in DMSO (ε = 41.8).

Energy = -379.233215 hartrees
Total enthalpy = 39.19 kcal/mol
Total entropy = 73.71 cal/mol×K

ATOM		X	Y	Z
1	C	-0.6661107000	-1.2631209000	-0.0006041000
2	C	-1.1209964000	0.1536704000	0.0002674000
3	C	0.6670916000	-1.2632648000	0.0011894000
4	C	1.1207228000	0.1536963000	0.0006510000
5	O	-0.0001122000	0.9630455000	0.0018531000
6	O	2.2235860000	0.6134381000	-0.0018984000
7	O	-2.2240814000	0.6126917000	-0.0009010000
8	H	-1.3616926000	-2.0994239000	-0.0036580000
9	H	1.3623098000	-2.0998650000	0.0022064000

Table S23. Energy, thermodynamic quantities, and corresponding xyz coordinates (in Angstroms; calculated with ωB97XD density functional and aug-cc-pVDZ basis set) of maleic anhydride.

Energy = -379.233336 hartrees
Total enthalpy = 39.19 kcal/mol
Total entropy = 73.71 cal/mol×K

ATOM		X	Y	Z
1	C	-0.6661107000	-1.2631209000	-0.0006041000
2	C	-1.1209964000	0.1536704000	0.0002674000
3	C	0.6670916000	-1.2632648000	0.0011894000
4	C	1.1207228000	0.1536963000	0.0006510000
5	O	-0.0001122000	0.9630455000	0.0018531000
6	O	2.2235860000	0.6134381000	-0.0018984000
7	O	-2.2240814000	0.6126917000	-0.0009010000
8	H	-1.3616926000	-2.0994239000	-0.0036580000
9	H	1.3623098000	-2.0998650000	0.0022064000

Table S24. Energy, thermodynamic quantities, and corresponding xyz coordinates (in Angstroms; calculated with ωB97XD density functional and aug-cc-pVDZ basis set) of N-methylmaleimide in DMSO (ε = 41.8).

Energy = -398.680549 hartrees
Total enthalpy = 65.92 kcal/mol
Total entropy = 82.82 cal/mol×K

ATOM		X	Y	Z
1	C	0.6387901000	-1.6402960000	-0.0019729000
2	C	-0.6947164000	-1.6169817000	-0.0002371000
3	C	1.1346924000	-0.2220450000	0.0019022000
4	H	1.3137978000	-2.4937460000	-0.0040756000
5	C	-1.1443879000	-0.1829924000	0.0011416000
6	H	-1.4000164000	-2.4456549000	-0.0004333000
7	N	0.0099937000	0.5901404000	0.0105444000
8	C	0.0448238000	2.0375723000	-0.0027738000
9	H	-0.9855649000	2.3959831000	0.1051950000
10	H	0.4617862000	2.4079669000	-0.9498231000
11	H	0.6528868000	2.4088912000	0.8327205000
12	O	2.2793207000	0.1744204000	-0.0027260000
13	O	-2.2778279000	0.2435839000	-0.0029933000

Table S25. Energy, thermodynamic quantities, and corresponding xyz coordinates (in Angstroms; calculated with ωB97XD density functional and aug-cc-pVDZ basis set) of maleimide.

Energy = -359.380445 hartrees
Total enthalpy = 47.27 kcal/mol
Total entropy = 74.56 cal/mol×K

ATOM		X	Y	Z
1	C	0.6367400000	-1.6409400000	-0.0005500000
2	C	-0.6964400000	-1.6157900000	0.0006200000
3	C	1.1347800000	-0.2233000000	-0.0002600000

4	H	1.3114600000	-2.4946600000	-0.0009300000
5	C	-1.1453000000	-0.1816300000	0.0003000000
6	H	-1.4033000000	-2.4432000000	0.0006300000
7	N	0.0104500000	0.5900300000	-0.0006400000
8	O	2.2799000000	0.1716800000	0.0005200000
9	O	-2.2784600000	0.2457000000	-0.0000700000
10	H	0.0296100000	1.6204000000	-0.0009000000

Table S26. Energy, thermodynamic quantities, and corresponding xyz coordinates (in Angstroms; calculated with ωB97XD density functional and aug-cc-pVDZ basis set) of N-phenylmaleimide.

Energy = -590.371005 hartrees
Total enthalpy = 100.83 kcal/mol
Total entropy = 99.67 cal/mol×K

ATOM		X	Y	Z
1	C	0.6367400000	-1.6409400000	-0.0005500000
2	C	-0.6964400000	-1.6157900000	0.0006200000
3	C	1.1347800000	-0.2233000000	-0.0002600000
4	H	1.3114600000	-2.4946600000	-0.0009300000
5	C	-1.1453000000	-0.1816300000	0.0003000000
6	H	-1.4033000000	-2.4432000000	0.0006300000
7	N	0.0104500000	0.5900300000	-0.0006400000
8	O	2.2799000000	0.1716800000	0.0005200000
9	O	-2.2784600000	0.2457000000	-0.0000700000
10	C	0.0359600000	2.0426100000	-0.0030700000
11	C	-1.0648600000	2.7976000000	0.4756800000
12	C	-1.0336200000	4.1951500000	0.4712400000
13	C	0.0834600000	4.8724900000	-0.0094300000
14	C	1.1771300000	4.1559500000	-0.4870500000
15	C	1.1615200000	2.7581300000	-0.4853600000
16	H	2.0257300000	2.2579700000	-0.8864200000
17	H	0.1015800000	5.9547300000	-0.0119600000
18	H	2.0426500000	4.6849000000	-0.8652800000
19	H	-1.9454900000	2.3279500000	0.8780200000
20	H	-1.8810200000	4.7544900000	0.8467100000

Table S27. Energy, thermodynamic quantities, and corresponding xyz coordinates (in Angstroms; calculated with ωB97XD density functional and aug-cc-pVDZ basis set) of N-methylmaleimide.

Energy = -398.680656 hartrees
Total enthalpy = 65.92 kcal/mol
Total entropy = 82.82 cal/mol×K

ATOM		X	Y	Z
1	C	0.6387901000	-1.6402960000	-0.0019729000
2	C	-0.6947164000	-1.6169817000	-0.0002371000
3	C	1.1346924000	-0.2220450000	0.0019022000
4	H	1.3137978000	-2.4937460000	-0.0040756000

5	C	-1.1443879000	-0.1829924000	0.0011416000
6	H	-1.4000164000	-2.4456549000	-0.0004333000
7	N	0.0099937000	0.5901404000	0.0105444000
8	C	0.0448238000	2.0375723000	-0.0027738000
9	H	-0.9855649000	2.3959831000	0.1051950000
10	H	0.4617862000	2.4079669000	-0.9498231000
11	H	0.6528868000	2.4088912000	0.8327205000
12	O	2.2793207000	0.1744204000	-0.0027260000
13	O	-2.2778279000	0.2435839000	-0.0029933000

Table S28. Energy, thermodynamic quantities, and corresponding xyz coordinates (in Angstroms; calculated with ωB97XD density functional and aug-cc-pVDZ basis set) of 4-methylstyrene N-methylmaleimide intermediate.

Energy = -747.608113 hartrees
Total enthalpy = 176.64 kcal/mol
Total entropy = 119.31 cal/mol×K

ATOM		X	Y	Z
1	C	-0.2824400000	-1.5189800000	0.4144500000
2	C	-1.3185300000	-1.4907500000	1.4379600000
3	C	-2.4696200000	-0.8139200000	1.2601800000
4	C	-2.6919800000	-0.0773200000	0.0171400000
5	C	-1.8032800000	-0.0337900000	-0.9817300000
6	C	-0.5116400000	-0.7970400000	-0.9019300000
7	C	0.8879100000	-2.1672200000	0.5730500000
8	C	1.9008500000	-2.1188800000	-0.5304900000
9	C	2.0588500000	-0.6781400000	-1.0566000000
10	C	2.8219100000	0.1705700000	-0.0530400000
11	C	0.7290000000	0.0705400000	-1.2624800000
12	C	0.8432400000	1.3107400000	-0.3895900000
13	H	0.6173600000	0.4176800000	-2.2996900000
14	H	-0.5460000000	-1.5598900000	-1.7046000000
15	H	1.1030200000	-2.7151900000	1.4937000000
16	H	-1.1334000000	-2.0308600000	2.3694800000
17	H	-3.2420900000	-0.7942000000	2.0294200000
18	H	-2.0036600000	0.5345000000	-1.8909300000
19	O	3.9152800000	-0.0583500000	0.4211900000
20	N	2.0649600000	1.2828600000	0.2610200000
21	O	0.0254500000	2.1984000000	-0.2672500000
22	H	2.6510200000	-0.6971600000	-1.9824300000
23	H	1.5696700000	-2.7523100000	-1.3729900000
24	H	2.8796800000	-2.4961300000	-0.2070700000
25	C	2.5098700000	2.3028600000	1.1903200000
26	H	1.7591300000	3.1007500000	1.1936800000
27	H	3.4815300000	2.7021200000	0.8710600000
28	H	2.6061700000	1.8795600000	2.1995000000
29	C	-3.9865000000	0.6816900000	-0.1163200000
30	H	-4.0673300000	1.2167900000	-1.0871200000
31	H	-4.0624800000	1.4315200000	0.6991200000

32	H	-4.8398800000	-0.0243900000	-0.0384200000

Table S29. Energy, thermodynamic quantities, and corresponding xyz coordinates (in Angstroms; calculated with ωB97XD density functional and aug-cc-pVDZ basis set) of 4-methylstyrene N-methylmaleimide double-Diels−Alder product.

Energy = -1146.360509 hartrees
Total enthalpy = 246.26 kcal/mol
Total entropy = 149.92 cal/mol×K

ATOM		X	Y	Z
1	C	0.5123900000	-1.4857500000	-0.0827800000
2	C	-0.7184200000	-1.3470400000	0.7768700000
3	C	-0.6646300000	0.0311800000	1.3979100000
4	C	-0.5826300000	1.0476400000	0.5275600000
5	C	-0.5428000000	0.5966900000	-0.9255100000
6	C	0.5946800000	-0.4351400000	-1.1711900000
7	C	1.4581000000	-2.4162700000	0.0489500000
8	C	2.6677500000	-2.3545200000	-0.8425700000
9	C	3.1247800000	-0.8984700000	-0.9829600000
10	C	3.6594700000	-0.3518200000	0.3327100000
11	C	2.0359100000	0.1187100000	-1.3864200000
12	C	2.3633300000	1.3291000000	-0.5390300000
13	H	2.1273700000	0.4224600000	-2.4341500000
14	H	0.3625600000	-0.9401800000	-2.1215000000
15	H	1.3816000000	-3.1926800000	0.8109700000
16	H	-0.7837800000	-2.1496100000	1.5173400000
17	H	-0.7441900000	0.1674000000	2.4755800000
18	H	-0.4742900000	1.4408100000	-1.6188600000
19	O	4.4151500000	-0.9033400000	1.1108600000
20	N	3.1843800000	0.9344600000	0.5012800000
21	O	2.0112500000	2.4777100000	-0.7293200000
22	H	3.9592900000	-0.8374200000	-1.6958100000
23	H	2.4446500000	-2.7529700000	-1.8441000000
24	H	3.4918000000	-2.9516500000	-0.4361400000
25	C	-1.9438900000	-1.3779100000	-0.1868800000
26	C	-1.8511700000	-0.2040300000	-1.1800300000
27	C	-3.2427800000	-1.1471100000	0.5548100000
28	C	-3.0956800000	0.6195700000	-0.9198500000
29	N	-3.8395100000	0.0012900000	0.0696800000
30	O	-3.4139500000	1.6553800000	-1.4727200000
31	O	-3.7177200000	-1.8286900000	1.4443400000
32	H	-1.8828900000	-0.5382400000	-2.2244200000
33	H	-1.9974700000	-2.3516400000	-0.6874500000
34	C	-5.0954600000	0.5117400000	0.5859600000
35	C	3.6052800000	1.8132400000	1.5759000000
36	H	-5.3425200000	1.4354600000	0.0472600000
37	H	-5.0077400000	0.7226700000	1.6375400000
38	H	-5.8806100000	-0.2434900000	0.4359200000
39	H	2.8572300000	2.6030000000	1.6853000000

40	H	4.5819800000	2.2585700000	1.3513200000
41	H	3.6731200000	1.2313000000	2.4997900000
42	C	-0.6825000000	2.4977800000	0.8901700000
43	H	-1.5792200000	2.9373400000	0.4285200000
44	H	0.1813400000	3.0612100000	0.5204600000
45	H	-0.7575900000	2.6222500000	1.9774200000

Table S30. Energy, thermodynamic quantities, and corresponding xyz coordinates (in Angstroms; calculated with ωB97XD density functional and aug-cc-pVDZ basis set) of 4-methylstyrene N-methylmaleimide ene product.

Energy = -1146.392027 hartrees
Total enthalpy = 245.85 kcal/mol
Total entropy = 158.22 cal/mol×K

ATOM		X	Y	Z
1	C	-0.0160900000	-0.6932500000	0.1661700000
2	C	-1.0111100000	-1.5728300000	-0.2675500000
3	C	-0.7827000000	-2.9455800000	-0.3631700000
4	C	0.4534900000	-3.4912800000	-0.0145600000
5	C	1.4513400000	-2.6133700000	0.4184700000
6	C	1.2379400000	-1.2373600000	0.4999000000
7	C	-0.2121500000	0.8091200000	0.3052700000
8	C	0.5318600000	1.2959700000	1.5578800000
9	C	2.0331800000	1.0437500000	1.4404700000
10	C	2.6735900000	2.0178600000	0.4676700000
11	C	2.4007500000	-0.3473100000	0.8932800000
12	C	3.2699000000	-0.0575400000	-0.3313500000
13	H	3.0410000000	-0.8906900000	1.5987200000
14	H	0.2447200000	1.2939900000	-0.5732600000
15	H	-1.9980600000	-1.2093700000	-0.5453100000
16	H	-1.5859700000	-3.5980000000	-0.7076900000
17	H	2.4286200000	-3.0125800000	0.6959800000
18	O	2.6133800000	3.2327300000	0.5002600000
19	N	3.3598000000	1.3117900000	-0.4983200000
20	O	3.8062500000	-0.8719000000	-1.0549200000
21	H	2.5101200000	1.2115800000	2.4138200000
22	H	0.1458700000	0.7696400000	2.4411400000
23	H	0.3608900000	2.3687300000	1.7097100000
24	C	-1.6576700000	1.3182200000	0.3513300000
25	C	-2.5861700000	0.7617700000	1.4382600000
26	C	-2.4458200000	1.1496400000	-0.9343500000
27	C	-3.9113900000	0.5509300000	0.7434600000
28	N	-3.7375500000	0.7766300000	-0.6132900000
29	O	-4.9663900000	0.2206100000	1.2501800000
30	O	-2.0583400000	1.3409000000	-2.0715700000
31	H	-2.7188600000	1.4374500000	2.2888900000
32	H	-1.5900400000	2.4099300000	0.4722800000
33	C	-4.7759600000	0.6061900000	-1.6119700000
34	C	4.0925800000	1.9569700000	-1.5723100000

35	H	-5.7008400000	0.3371500000	-1.0958800000
36	H	-4.4939500000	-0.1897100000	-2.3107200000
37	H	-4.9145500000	1.5424100000	-2.1636000000
38	H	4.5703600000	1.1744900000	-2.1659700000
39	H	4.8688600000	2.6336000000	-1.1370800000
40	H	3.3945600000	2.5299200000	-2.2108300000
41	H	-2.2497800000	-0.2071800000	1.8303800000
42	C	0.7201000000	-4.9730000000	-0.0906800000
43	H	-0.1734000000	-5.5194600000	-0.4133100000
44	H	1.0187400000	-5.3679900000	0.9038000000
45	H	1.5305000000	-5.1880500000	-0.8093300000

Table S31. Energy, thermodynamic quantities, and corresponding xyz coordinates (in Angstroms; calculated with ωB97XD density functional and aug-cc-pVDZ basis set) of 4-methylstyrene.

Energy = -348.886141 hartrees
Total enthalpy = 107.21 kcal/mol
Total entropy = 89.92 cal/mol×K

ATOM		X	Y	Z
1	C	1.4292700000	0.2294100000	0.0014700000
2	C	0.6031700000	1.3626200000	0.0000800000
3	C	-0.7860700000	1.2538500000	-0.0008100000
4	C	-1.3581100000	-0.0139700000	-0.0000400000
5	C	-0.5681900000	-1.1629700000	0.0016600000
6	C	0.8163300000	-1.0343100000	0.0024400000
7	C	2.8952400000	0.4128000000	0.0020200000
8	C	3.8216400000	-0.5519100000	-0.0038800000
9	H	3.2254200000	1.4570500000	0.0075800000
10	H	3.5688100000	-1.6154900000	-0.0104100000
11	H	4.8835800000	-0.2979200000	-0.0027600000
12	H	1.0429000000	2.3526900000	-0.0005700000
13	H	-1.0322900000	-2.1496400000	0.0025600000
14	H	1.4222300000	-1.9418000000	0.0042500000
15	H	-1.4147100000	2.1447000000	-0.0020200000
16	C	-2.8529900000	-0.1610500000	-0.0008700000
17	H	-3.3664600000	0.8241800000	-0.0021500000
18	H	-3.1736700000	-0.7195300000	0.9038200000
19	H	-3.1725000000	-0.7212800000	-0.9048900000

Table S32. Energy, thermodynamic quantities, and corresponding xyz coordinates (in Angstroms; calculated with ωB97XD density functional and aug-cc-pVDZ basis set) of 4-methylstyrene N-methylmaleimide first transition state.

Energy = -747.545973 hartrees
Total enthalpy = 173.89 kcal/mol
Total entropy = 122.24 cal/mol×K

ATOM	X	Y	Z

1	C	-2.8829947000	0.0548424000	-0.0772414000
2	C	-2.3224907000	-0.0632830000	-1.3800290000
3	C	-2.2326595000	0.8417220000	0.8581196000
4	C	-1.1430949000	0.5553109000	-1.7023054000
5	C	1.7627021000	2.1556263000	0.0284252000
6	C	0.8886545000	1.7437249000	-0.9935972000
7	C	-0.4306436000	1.3225701000	-0.7307709000
8	C	-1.0061575000	1.4574353000	0.5599380000
9	H	-2.8428800000	-0.6666646000	-2.1277189000
10	H	-2.6653779000	0.9710689000	1.8521310000
11	H	-0.7111470000	0.4385592000	-2.6990746000
12	H	2.7685975000	2.4494327000	-0.2839215000
13	H	1.3672942000	2.7692535000	0.8422487000
14	H	1.3047204000	1.5097910000	-1.9778055000
15	H	-0.5844086000	2.1619354000	1.2746858000
16	O	-0.4526358000	-1.8237704000	1.2437953000
17	N	1.3853618000	-1.2641870000	-0.0604931000
18	O	3.4503636000	-0.4414750000	-0.6720927000
19	C	0.4793668000	-1.0663309000	1.0130249000
20	C	2.4447774000	-0.3922165000	0.0122158000
21	C	2.0790471000	0.6298333000	1.0697052000
22	C	0.9281832000	0.1229680000	1.7088164000
23	H	2.9015007000	1.0675304000	1.6398070000
24	H	0.5283157000	0.4269862000	2.6716914000
25	C	1.2721639000	-2.3308752000	-1.0268959000
26	H	0.5726483000	-3.0768208000	-0.6306351000
27	H	2.2549342000	-2.7916093000	-1.1921100000
28	H	0.8859694000	-1.9519166000	-1.9859297000
29	C	-4.1649884000	-0.6601046000	0.2447969000
30	H	-4.5226639000	-0.4112663000	1.2531042000
31	H	-4.0163128000	-1.7502223000	0.1891663000
32	H	-4.9517388000	-0.4021232000	-0.4810224000

Table S33. Energy, thermodynamic quantities, and corresponding xyz coordinates (in Angstroms; calculated with ωB97XD density functional and aug-cc-pVDZ basis set) of 4-methylstyrene N-methylmaleimide second transition state for Diels−Alder mechanism.

Energy = -1146.275799 hartrees
Total enthalpy = 242.94 kcal/mol
Total entropy = 157.80 cal/mol×K

ATOM		X	Y	Z
1	C	-0.6974600000	1.3090000000	0.2466400000
2	C	0.3012000000	0.9922300000	1.2643500000
3	C	0.8020900000	-0.2999000000	1.3591700000
4	C	0.8306100000	-1.0774400000	0.1988400000
5	C	0.4494700000	-0.5223300000	-1.0188100000
6	C	-0.6783100000	0.4856500000	-1.0205200000
7	C	-1.6687300000	2.2266900000	0.3853800000
8	C	-2.7011700000	2.3262600000	-0.6992700000

9	C	-3.1980300000	0.9157700000	-1.0829700000
10	C	-4.0489700000	0.3485500000	0.0415100000
11	C	-2.0858400000	-0.1317800000	-1.2864100000
12	C	-2.4499800000	-1.2686500000	-0.3443800000
13	H	-2.0896000000	-0.5410200000	-2.3059100000
14	H	-0.5311800000	1.1622500000	-1.8779100000
15	H	-1.7454200000	2.8404500000	1.2857900000
16	H	0.3779700000	1.6588600000	2.1261800000
17	H	1.3096200000	-0.6363700000	2.2636200000
18	H	0.5837400000	-1.1082700000	-1.9301600000
19	O	-5.0109400000	0.8690200000	0.5676000000
20	N	-3.5552600000	-0.8911500000	0.3994300000
21	O	-1.8984900000	-2.3446700000	-0.2455400000
22	H	-3.8392600000	0.9871500000	-1.9730000000
23	H	-2.2712600000	2.7985000000	-1.5998100000
24	H	-3.5599900000	2.9358700000	-0.3902700000
25	C	1.6333100000	-2.6333100000	0.2390600000
26	C	-4.1661800000	-1.7157600000	1.4230600000
27	H	-3.5348200000	-2.5993200000	1.5675300000
28	H	-5.1722900000	-2.0274600000	1.1104200000
29	H	-4.2399300000	-1.1492000000	2.3604600000
30	H	1.6607100000	-3.0748500000	-0.7784800000
31	H	1.0797200000	-3.3146300000	0.9172200000
32	H	2.6705500000	-2.5102800000	0.6107300000
33	C	2.0092400000	1.8160000000	-0.1658200000
34	C	2.0093600000	0.9573400000	-1.2609300000
35	C	3.1340700000	1.4298700000	0.7181000000
36	C	3.1918300000	0.0527400000	-1.1199100000
37	N	3.7233800000	0.3042500000	0.1371700000
38	O	3.6240200000	-0.7567200000	-1.9155100000
39	O	3.5285400000	1.9453900000	1.7463600000
40	H	1.6390700000	1.2052700000	-2.2538600000
41	H	1.5529800000	2.8002100000	-0.1085700000
42	C	4.8423200000	-0.4073900000	0.7150700000
43	H	5.2151100000	-1.1122900000	-0.0372700000
44	H	4.5254800000	-0.9622600000	1.6096600000
45	H	5.6643400000	0.3001400000	0.9534100000

Table S34. Energy, thermodynamic quantities, and corresponding xyz coordinates (in Angstroms; calculated with ωB97XD density functional and aug-cc-pVDZ basis set) of 4-methylstyrene N-methylmaleimide second transition state for ene mechanism.

Energy = -1146.277640 hartrees
Total enthalpy = 241.87 kcal/mol
Total entropy = 154.90 cal/mol×K

ATOM		X	Y	Z
1	C	0.1059700000	0.5286800000	-0.7311000000
2	C	-0.5229600000	-0.1687700000	-1.8127900000
3	C	-0.9377600000	-1.4574200000	-1.6719500000

4	C	-0.7608100000	-2.1270300000	-0.4128900000
5	C	-0.1473900000	-1.5461900000	0.6416400000
6	C	0.2981400000	-0.1467000000	0.5588400000
7	C	0.2895800000	1.9106500000	-0.7291700000
8	C	1.2817600000	2.5243500000	0.2399900000
9	C	2.2566200000	1.4878000000	0.7912700000
10	C	3.3005400000	1.0546000000	-0.2281600000
11	C	1.6443400000	0.1645100000	1.2719500000
12	C	2.6785500000	-0.8783100000	0.8572700000
13	H	1.5075300000	0.1152700000	2.3590000000
14	H	0.0951300000	2.4512200000	-1.6587300000
15	H	-0.6641200000	0.3525400000	-2.7607300000
16	H	-1.4037200000	-1.9918500000	-2.5000800000
17	H	0.0174800000	-2.0933100000	1.5695100000
18	O	3.8823300000	1.7506600000	-1.0321300000
19	N	3.5362900000	-0.2982800000	-0.0597200000
20	O	2.7409300000	-2.0318500000	1.2250000000
21	H	2.8278100000	1.9419400000	1.6162200000
22	H	0.7874400000	3.0163100000	1.0889500000
23	H	1.8554000000	3.3056300000	-0.2789300000
24	C	4.5789500000	-1.0150500000	-0.7682700000
25	H	4.6349600000	-2.0261000000	-0.3502100000
26	H	5.5389700000	-0.4996900000	-0.6353100000
27	H	4.3409000000	-1.0693000000	-1.8393600000
28	H	-0.4849000000	0.5108300000	1.2208900000
29	C	-1.2258600000	-3.5519400000	-0.2739900000
30	H	-1.6963000000	-3.9257800000	-1.2080600000
31	H	-1.9748600000	-3.6221100000	0.5431600000
32	H	-0.3623800000	-4.2077500000	-0.0330400000
33	C	-1.5438200000	2.4032100000	0.2962800000
34	C	-1.5939200000	1.7138500000	1.5059800000
35	C	-2.5665800000	1.7814900000	-0.6066300000
36	C	-2.6221700000	0.6568500000	1.3965100000
37	N	-3.1039800000	0.7076500000	0.0837200000
38	O	-2.9902200000	-0.1495800000	2.2291400000
39	O	-2.8762000000	2.1003400000	-1.7372200000
40	H	-1.2343200000	2.0586300000	2.4738500000
41	H	-1.2897600000	3.4540400000	0.1734400000
42	C	-4.0398500000	-0.2352700000	-0.4831600000
43	H	-4.3583600000	-0.9149900000	0.3156300000
44	H	-3.5567600000	-0.8141000000	-1.2855800000
45	H	-4.9127000000	0.2895800000	-0.8940000000

Table S35. Energy, thermodynamic quantities, and corresponding xyz coordinates (in Angstroms; calculated with ωB97XD density functional and aug-cc-pVDZ basis set) of 4-methoxystyrene maleimide intermediate.

Energy = -783.497075 hartrees
Total enthalpy = 161.10 kcal/mol
Total entropy = 118.32 cal/mol×K

ATOM		X	Y	Z
1	C	-0.3394300000	-1.4278200000	0.4244000000
2	C	-1.3673500000	-1.3022100000	1.4496100000
3	C	-2.4645200000	-0.5458400000	1.2546200000
4	C	-2.6564700000	0.1880700000	-0.0009200000
5	C	-1.7610200000	0.1262600000	-0.9975600000
6	C	-0.5175300000	-0.7091100000	-0.9022600000
7	C	0.7849600000	-2.1503800000	0.5969600000
8	C	1.8023200000	-2.1848900000	-0.5033100000
9	C	2.0577500000	-0.7639700000	-1.0455900000
10	C	2.8721700000	0.0413700000	-0.0471100000
11	C	0.7818000000	0.0700400000	-1.2664900000
12	C	0.9801300000	1.3130700000	-0.4134800000
13	H	0.6961300000	0.4082800000	-2.3091800000
14	H	-0.5852200000	-1.4832400000	-1.6922300000
15	H	0.9626700000	-2.6952100000	1.5276000000
16	H	-1.2111000000	-1.8257800000	2.3960900000
17	H	-3.2144600000	-0.4370200000	2.0405400000
18	O	-3.7535500000	1.0074700000	-0.1111800000
19	H	-1.9332200000	0.6947200000	-1.9135000000
20	O	3.9449100000	-0.2564800000	0.4370800000
21	N	2.1926700000	1.2066300000	0.2488000000
22	O	0.2334300000	2.2639100000	-0.3128800000
23	H	2.6523100000	-0.8328200000	-1.9677400000
24	H	1.4343600000	-2.8056000000	-1.3400900000
25	H	2.7534400000	-2.6209700000	-0.1706500000
26	C	-5.0031400000	0.3294500000	-0.0971900000
27	H	-5.7809900000	1.1012000000	-0.1528400000
28	H	-5.1472900000	-0.2557100000	0.8267400000
29	H	-5.0913600000	-0.3453400000	-0.9658200000
30	H	2.5505000000	1.9239500000	0.8966700000

Table S36. Energy, thermodynamic quantities, and corresponding xyz coordinates (in Angstroms; calculated with ωB97XD density functional and aug-cc-pVDZ basis set) of 4-methoxystyrene N-phenylmaleimide intermediate.

Energy = -1014.489074 hartrees
Total enthalpy = 215.10 kcal/mol
Total entropy = 139.71 cal/mol×K

ATOM		X	Y	Z
1	C	1.6607774000	-1.1412525000	-1.0246774000
2	C	2.1959647000	-0.0578200000	-1.8391433000
3	C	2.9242369000	0.9361112000	-1.2949861000
4	C	3.1970236000	0.9633159000	0.1453996000
5	C	2.7250622000	0.0188266000	0.9713152000
6	C	1.9074396000	-1.1375216000	0.4750322000
7	C	0.9061281000	-2.1426967000	-1.5194553000
8	C	0.3421756000	-3.1550847000	-0.5676809000

9	C	-0.3032306000	-2.4463025000	0.6445479000
10	C	-1.5992654000	-1.7831353000	0.2111685000
11	C	0.5511404000	-1.3065127000	1.2261073000
12	C	-0.3176202000	-0.0664631000	1.1107348000
13	H	0.7632856000	-1.4594889000	2.2936333000
14	H	2.4629746000	-2.0609965000	0.7301428000
15	H	0.6812393000	-2.2027704000	-2.5872139000
16	H	1.9711776000	-0.0630572000	-2.9086430000
17	H	3.2951267000	1.7603056000	-1.9074437000
18	O	3.8999739000	2.0314695000	0.6479162000
19	H	2.9374129000	0.0809444000	2.0404657000
20	O	-2.5519140000	-2.3147321000	-0.3140570000
21	N	-1.5167048000	-0.4234133000	0.4944605000
22	O	-0.0562969000	1.0556051000	1.4807286000
23	H	-0.5538165000	-3.1937039000	1.4102496000
24	H	1.1448573000	-3.8106600000	-0.1852809000
25	H	-0.4028495000	-3.8017672000	-1.0489869000
26	C	5.2587029000	2.0868087000	0.2325263000
27	H	5.6850345000	3.0003982000	0.6653038000
28	H	5.3536745000	2.1323747000	-0.8656605000
29	H	5.8148222000	1.2089110000	0.6035767000
30	C	-2.5312504000	0.5192435000	0.1490255000
31	C	-2.2481202000	1.4949229000	-0.8066951000
32	C	-3.2309003000	2.4202874000	-1.1518172000
33	C	-4.4899678000	2.3616110000	-0.5518958000
34	C	-4.7650464000	1.3782050000	0.3996375000
35	C	-3.7834827000	0.4546116000	0.7581706000
36	H	-1.2625170000	1.5235316000	-1.2740309000
37	H	-3.0132614000	3.1867430000	-1.8975311000
38	H	-5.2601498000	3.0842675000	-0.8275266000
39	H	-5.7483696000	1.3304576000	0.8706550000
40	H	-3.9844149000	-0.3132646000	1.5064782000

Table S37. Energy, thermodynamic quantities, and corresponding xyz coordinates (in Angstroms; calculated with ωB97XD density functional and aug-cc-pVDZ basis set) of 4-methoxystyrene N-methylmaleimide intermediate.

Energy = -822.798923 hartrees
Total enthalpy = 180.17 kcal/mol
Total entropy = 126.72 cal/mol×K

ATOM		X	Y	Z
1	C	-0.3136691000	-1.4145782000	0.4517354000
2	C	-1.3152602000	-1.2288175000	1.4943599000
3	C	-2.4166431000	-0.4829149000	1.2843116000
4	C	-2.6421893000	0.1745908000	-0.0062962000
5	C	-1.7647353000	0.0705674000	-1.0141935000
6	C	-0.5080175000	-0.7418357000	-0.8981742000
7	C	0.8150543000	-2.1301011000	0.6260250000
8	C	1.8126594000	-2.1835822000	-0.4925291000

9	C	2.0618416000	-0.7659700000	-1.0488980000
10	C	2.8546050000	0.0553557000	-0.0464201000
11	C	0.7783289000	0.0494317000	-1.2871554000
12	C	0.9571782000	1.3070844000	-0.4509703000
13	H	0.6805306000	0.3656628000	-2.3352659000
14	H	-0.5654320000	-1.5385975000	-1.6653436000
15	H	1.0198272000	-2.6357931000	1.5728897000
16	H	-1.1334428000	-1.6957774000	2.4655552000
17	H	-3.1445963000	-0.3222396000	2.0818448000
18	O	-3.7516131000	0.9744845000	-0.1364493000
19	H	-1.9615072000	0.5873383000	-1.9555845000
20	O	3.9259734000	-0.2268842000	0.4504806000
21	N	2.1553419000	1.2108509000	0.2398848000
22	O	0.2060967000	2.2567092000	-0.3752565000
23	H	2.6650432000	-0.8368684000	-1.9652793000
24	H	1.4302426000	-2.8079326000	-1.3197266000
25	H	2.7674858000	-2.6196521000	-0.1710647000
26	C	-4.9903316000	0.2782736000	-0.0825967000
27	H	-5.7815033000	1.0355129000	-0.1501114000
28	H	-5.1083679000	-0.2802053000	0.8612438000
29	H	-5.0803174000	-0.4235699000	-0.9293120000
30	C	2.6400344000	2.2190170000	1.1615456000
31	H	1.8774168000	3.0025079000	1.2304799000
32	H	3.5827031000	2.6479867000	0.7952051000
33	H	2.8077333000	1.7720692000	2.1506146000

Table S38. Energy, thermodynamic quantities, and corresponding xyz coordinates (in Angstroms; calculated with ωB97XD density functional and aug-cc-pVDZ basis set) of 4-methoxystyrene maleimide double-Diels−Alder product.

Energy = -1142.952546 hartrees
Total enthalpy = 212.63 kcal/mol
Total entropy = 138.05 cal/mol×K

ATOM		X	Y	Z
1	C	0.5877600000	-1.5354000000	0.1731400000
2	C	-0.6363500000	-1.2192000000	0.9953200000
3	C	-0.6421000000	0.2755200000	1.2644000000
4	C	-0.6249300000	1.0115800000	0.1384300000
5	C	-0.5562700000	0.2238400000	-1.1478000000
6	C	0.6419100000	-0.7618300000	-1.1294100000
7	C	1.5563700000	-2.4006900000	0.4789100000
8	C	2.7306800000	-2.5199500000	-0.4550300000
9	C	3.1730900000	-1.1176200000	-0.8972400000
10	C	3.7671700000	-0.3602200000	0.2818600000
11	C	2.0572400000	-0.1858500000	-1.4186400000
12	C	2.3430300000	1.1392300000	-0.7324600000
13	H	2.1378700000	-0.0101400000	-2.4991600000
14	H	0.4689100000	-1.4800100000	-1.9489600000
15	H	1.5216800000	-2.9878100000	1.4000300000

16	H	-0.6686100000	-1.8081900000	1.9190700000
17	H	-0.7147800000	0.6698800000	2.2763700000
18	O	-0.7477200000	2.3429200000	-0.0043500000
19	H	-0.5315800000	0.8886500000	-2.0188600000
20	O	4.5797900000	-0.7673900000	1.0867500000
21	N	3.2507900000	0.9208100000	0.2918800000
22	O	1.8949900000	2.2273800000	-1.0251600000
23	H	3.9731700000	-1.1985300000	-1.6493400000
24	H	2.4688600000	-3.1113200000	-1.3488000000
25	H	3.5798400000	-3.0212900000	0.0286400000
26	C	-0.8091300000	3.0926400000	1.1965500000
27	H	-0.8834400000	4.1453400000	0.9013200000
28	H	0.1018900000	2.9387400000	1.7986300000
29	H	-1.6915400000	2.8112500000	1.7955300000
30	C	-1.8616800000	-1.5544600000	0.0944200000
31	C	-1.8233500000	-0.6753600000	-1.1703700000
32	C	-3.1662200000	-1.1946000000	0.7736400000
33	C	-3.1022400000	0.1404700000	-1.1132900000
34	N	-3.8099300000	-0.2281200000	0.0192800000
35	O	-3.4651500000	0.9849800000	-1.9055900000
36	O	-3.6057000000	-1.6412600000	1.8130400000
37	H	-1.8383000000	-1.2687000000	-2.0953000000
38	H	-1.8718500000	-2.6285400000	-0.1368400000
39	H	-4.7162500000	0.1865100000	0.2817500000
40	H	3.5617100000	1.6540500000	0.9460200000

Table S39. Energy, thermodynamic quantities, and corresponding xyz coordinates (in Angstroms; calculated with ωB97XD density functional and aug-cc-pVDZ basis set) of 4-methoxystyrene N-phenylmaleimide double-Diels−Alder product.

Energy = -1604.941099 hartrees
Total enthalpy = 319.62 kcal/mol
Total entropy = 186.77 cal/mol×K

ATOM		X	Y	Z
1	C	-0.9113876000	2.1680823000	0.6696070000
2	C	0.2565518000	1.4296613000	1.2752364000
3	C	0.1329604000	-0.0263814000	0.8606699000
4	C	0.1078647000	-0.1905038000	-0.4737807000
5	C	0.1636882000	1.0860255000	-1.2832520000
6	C	-0.9594382000	2.0624149000	-0.8422548000
7	C	-1.8511598000	2.8468015000	1.3305900000
8	C	-3.0239134000	3.3895610000	0.5584281000
9	C	-3.4877069000	2.3194967000	-0.4400408000
10	C	-3.9618994000	1.0803601000	0.3097906000
11	C	-2.3965557000	1.7831045000	-1.3850673000
12	C	-2.6964782000	0.3000374000	-1.4621535000
13	H	-2.4722164000	2.1971596000	-2.3974652000
14	H	-0.6967748000	3.0522948000	-1.2508207000
15	H	-1.8132936000	2.9504794000	2.4177975000

16	H	0.2904792000	1.5495007000	2.3639370000
17	H	0.1230898000	-0.8273405000	1.5973967000
18	O	0.1224453000	-1.3323046000	-1.1851848000
19	H	0.1301451000	0.8761181000	-2.3582964000
20	O	-4.6703819000	1.0462225000	1.2909244000
21	N	-3.4570511000	-0.0439003000	-0.3394783000
22	O	-2.3503392000	-0.4763127000	-2.3217108000
23	H	-4.3421349000	2.6883816000	-1.0281399000
24	H	-2.7588300000	4.3044985000	0.0025746000
25	H	-3.8567840000	3.6443759000	1.2269683000
26	C	0.0660620000	-2.5355042000	-0.4349293000
27	H	0.0522520000	-3.3526456000	-1.1648281000
28	H	-0.8480893000	-2.5700512000	0.1803751000
29	H	0.9502900000	-2.6335138000	0.2171312000
30	C	1.5466121000	2.0075811000	0.6234486000
31	C	1.4947896000	1.7871725000	-0.8984470000
32	C	2.7724561000	1.2492721000	1.0884639000
33	C	2.6875287000	0.9062146000	-1.2105660000
34	N	3.3656795000	0.6446935000	-0.0217531000
35	O	3.0121897000	0.4741669000	-2.2939310000
36	O	3.1814518000	1.1424433000	2.2224173000
37	H	1.5996393000	2.7219799000	-1.4660794000
38	H	1.6649008000	3.0652470000	0.8960483000
39	C	4.4886033000	-0.2309954000	0.0684308000
40	C	4.3364253000	-1.5712179000	-0.2871940000
41	C	5.4298870000	-2.4301562000	-0.1913666000
42	C	6.6589090000	-1.9519398000	0.2668717000
43	C	6.7980868000	-0.6102935000	0.6246816000
44	C	5.7127660000	0.2586389000	0.5204534000
45	H	5.8105756000	1.3110901000	0.7892844000
46	H	3.3674215000	-1.9323857000	-0.6351023000
47	H	5.3176585000	-3.4790460000	-0.4708308000
48	H	7.7577589000	-0.2336839000	0.9826263000
49	H	7.5115890000	-2.6286628000	0.3467638000
50	C	-3.6103121000	-1.3819574000	0.1265484000
51	C	-4.0777307000	-2.3801828000	-0.7274815000
52	C	-4.1539463000	-3.6932105000	-0.2617646000
53	C	-3.7818256000	-4.0000199000	1.0470397000
54	C	-3.3259323000	-2.9908351000	1.8974290000
55	C	-3.2319642000	-1.6793859000	1.4377668000
56	H	-2.8540373000	-0.8881618000	2.0856081000
57	H	-4.3669003000	-2.1329569000	-1.7482870000
58	H	-4.5118209000	-4.4791671000	-0.9290821000
59	H	-3.8453362000	-5.0287705000	1.4064067000
60	H	-3.0305486000	-3.2250595000	2.9215696000

Table S40. Energy, thermodynamic quantities, and corresponding xyz coordinates (in Angstroms; calculated with ωB97XD density functional and aug-cc-pVDZ basis set) of 4-methoxystyrene N-methylmaleimide double-Diels−Alder product.

Energy = -1221.556927 hartrees
Total enthalpy = 249.23 kcal/mol
Total entropy = 153.08 cal/mol×K

ATOM		X	Y	Z
1	C	0.5877585000	-1.5353960000	0.1731352000
2	C	-0.6363519000	-1.2192001000	0.9953212000
3	C	-0.6421009000	0.2755152000	1.2643975000
4	C	-0.6249344000	1.0115755000	0.1384302000
5	C	-0.5562655000	0.2238381000	-1.1477956000
6	C	0.6419091000	-0.7618279000	-1.1294126000
7	C	1.5563742000	-2.4006941000	0.4789126000
8	C	2.7306774000	-2.5199547000	-0.4550266000
9	C	3.1730878000	-1.1176157000	-0.8972404000
10	C	3.7671684000	-0.3602185000	0.2818583000
11	C	2.0572435000	-0.1858481000	-1.4186350000
12	C	2.3430327000	1.1392261000	-0.7324582000
13	H	2.1378696000	-0.0101384000	-2.4991581000
14	H	0.4689132000	-1.4800113000	-1.9489612000
15	H	1.5216816000	-2.9878135000	1.4000322000
16	H	-0.6686137000	-1.8081884000	1.9190695000
17	H	-0.7147781000	0.6698821000	2.2763702000
18	O	-0.7477198000	2.3429241000	-0.0043484000
19	H	-0.5315799000	0.8886502000	-2.0188566000
20	O	4.5797870000	-0.7673856000	1.0867525000
21	N	3.2507859000	0.9208073000	0.2918849000
22	O	1.8949907000	2.2273789000	-1.0251553000
23	H	3.9731655000	-1.1985329000	-1.6493396000
24	H	2.4688597000	-3.1113172000	-1.3487957000
25	H	3.5798423000	-3.0212889000	0.0286422000
26	C	-0.8091329000	3.0926363000	1.1965548000
27	H	-0.8834368000	4.1453429000	0.9013221000
28	H	0.1018911000	2.9387381000	1.7986267000
29	H	-1.6915372000	2.8112505000	1.7955298000
30	C	-1.8616817000	-1.5544583000	0.0944210000
31	C	-1.8233540000	-0.6753579000	-1.1703668000
32	C	-3.1662186000	-1.1945955000	0.7736397000
33	C	-3.1022382000	0.1404702000	-1.1132943000
34	N	-3.8099339000	-0.2281172000	0.0192784000
35	O	-3.4651512000	0.9849766000	-1.9055857000
36	O	-3.6056954000	-1.6412598000	1.8130443000
37	H	-1.8383015000	-1.2687048000	-2.0953030000
38	H	-1.8718492000	-2.6285441000	-0.1368415000
39	C	-5.0782815000	0.3533654000	0.4107326000
40	C	3.6828061000	1.9425366000	1.2240866000
41	H	-5.3593069000	1.0917379000	-0.3483958000
42	H	-4.9810985000	0.8433775000	1.3890654000
43	H	-5.8478405000	-0.4277451000	0.4727186000
44	H	3.2470597000	2.8955835000	0.9041464000
45	H	4.7784798000	2.0133859000	1.2223792000
46	H	3.3379366000	1.6984525000	2.2383851000

Table S41. Energy, thermodynamic quantities, and corresponding xyz coordinates (in Angstroms; calculated with ωB97XD density functional and aug-cc-pVDZ basis set) of 4-methoxystyrene maleimide ene product.

Energy = -1142.984021 hartrees
Total enthalpy = 212.32 kcal/mol
Total entropy = 144.62 cal/mol×K

ATOM		X	Y	Z
1	C	-0.0540000000	-0.4613600000	0.2173200000
2	C	-1.1510600000	-1.2526300000	-0.1239400000
3	C	-1.0848800000	-2.6467000000	-0.1695900000
4	C	0.1168200000	-3.2877000000	0.1351100000
5	C	1.2320200000	-2.5129300000	0.4741000000
6	C	1.1532400000	-1.1254800000	0.5174700000
7	C	-0.0776000000	1.0583900000	0.3009400000
8	C	0.7252500000	1.4920400000	1.5358700000
9	C	2.1896300000	1.0651100000	1.4305100000
10	C	2.9539800000	1.9940000000	0.5005400000
11	C	2.4059300000	-0.3382400000	0.8334700000
12	C	3.2282300000	-0.0981700000	-0.4336900000
13	H	3.0485600000	-0.9447600000	1.4882500000
14	H	0.4276200000	1.4597300000	-0.5969500000
15	H	-2.1129300000	-0.8025800000	-0.3671300000
16	H	-1.9792300000	-3.2088700000	-0.4348500000
17	H	2.1697100000	-3.0211700000	0.7060400000
18	O	3.0647600000	3.1984300000	0.5984300000
19	N	3.5148500000	1.2525100000	-0.5219900000
20	O	3.5920200000	-0.9321100000	-1.2336900000
21	H	2.6630000000	1.1475700000	2.4188400000
22	H	0.2768300000	1.0432800000	2.4357500000
23	H	0.6861700000	2.5848100000	1.6570300000
24	O	0.3038900000	-4.6298000000	0.1316900000
25	C	-0.7982000000	-5.4505500000	-0.2201600000
26	H	-0.4323200000	-6.4827900000	-0.1785600000
27	H	-1.1522100000	-5.2293000000	-1.2404100000
28	H	-1.6333300000	-5.3297800000	0.4893900000
29	C	-1.4594000000	1.7243400000	0.3181400000
30	C	-2.4455000000	1.3219800000	1.4199800000
31	C	-2.2479700000	1.5600500000	-0.9707300000
32	C	-3.7835800000	1.2037900000	0.7231700000
33	N	-3.5720000000	1.3221000000	-0.6454800000
34	O	-4.8691100000	1.0196800000	1.2306700000
35	O	-1.8326900000	1.6472200000	-2.1075700000
36	H	-2.5162200000	2.0492800000	2.2383700000
37	H	-1.2754200000	2.8098200000	0.3821400000
38	H	-2.2081500000	0.3432100000	1.8647400000
39	H	4.0703300000	1.6662700000	-1.2852300000
40	H	-4.3216000000	1.2274700000	-1.3464700000

Table S42. Energy, thermodynamic quantities, and corresponding xyz coordinates (in Angstroms; calculated with ωB97XD density functional and aug-cc-pVDZ basis set) of 4-methoxystyrene N-phenylmaleimide ene product.

Energy = -1604.968554 hartrees
Total enthalpy = 319.88 kcal/mol
Total entropy = 190.29 cal/mol×K

ATOM		X	Y	Z
1	C	0.0052988000	-1.3330441000	0.4668728000
2	C	-1.1730242000	-1.7995078000	-0.1146822000
3	C	-1.2509544000	-3.0378889000	-0.7557838000
4	C	-0.1160558000	-3.8472543000	-0.8274485000
5	C	1.0827714000	-3.3884279000	-0.2680770000
6	C	1.1463298000	-2.1561565000	0.3709862000
7	C	0.1392910000	-0.0189431000	1.2220409000
8	C	0.9855047000	-0.2724593000	2.4781634000
9	C	2.4057902000	-0.7016894000	2.1019822000
10	C	3.1999965000	0.5134704000	1.6520540000
11	C	2.4762399000	-1.6792400000	0.9136618000
12	C	3.2450206000	-0.9237840000	-0.1707830000
13	H	3.0945329000	-2.5533988000	1.1635648000
14	H	0.6837036000	0.6956070000	0.5774375000
15	H	-2.0866631000	-1.2061677000	-0.0849689000
16	H	-2.2030288000	-3.3525787000	-1.1808816000
17	H	1.9683872000	-4.0225996000	-0.3379839000
18	O	3.4190076000	1.5166434000	2.2926669000
19	N	3.6254663000	0.3128158000	0.3417723000
20	O	3.4943008000	-1.3064580000	-1.2897105000
21	H	2.9123712000	-1.1120075000	2.9859046000
22	H	0.5112732000	-1.0580275000	3.0862254000
23	H	1.0448226000	0.6357271000	3.0958721000
24	O	-0.0704796000	-5.0715194000	-1.4061518000
25	C	-1.2742933000	-5.5946521000	-1.9442315000
26	H	-1.0245982000	-6.5881109000	-2.3338522000
27	H	-1.6544923000	-4.9660194000	-2.7660597000
28	H	-2.0517040000	-5.6898277000	-1.1684225000
29	C	-1.1638799000	0.6964278000	1.5937961000
30	C	-2.2031276000	-0.0559861000	2.4299350000
31	C	-1.9357571000	1.2168641000	0.3947968000
32	C	-3.5378881000	0.2928278000	1.8084780000
33	N	-3.2935477000	1.0009808000	0.6270526000
34	O	-4.6425912000	0.0201286000	2.2179199000
35	O	-1.4840054000	1.7562634000	-0.5900427000
36	H	-2.2077029000	0.2306668000	3.4889078000
37	H	-0.8690509000	1.6125149000	2.1327454000
38	H	-2.0809478000	-1.1488667000	2.3833038000
39	C	4.2937890000	1.3154797000	-0.4263042000
40	C	-4.3199196000	1.4761081000	-0.2444011000

41	C	-5.1317944000	0.5679320000	-0.9228601000
42	C	-6.1279554000	1.0474238000	-1.7732023000
43	C	-6.2989605000	2.4214652000	-1.9488375000
44	C	-5.4769147000	3.3216978000	-1.2690281000
45	C	-4.4864559000	2.8507739000	-0.4092293000
46	H	-3.8424353000	3.5435571000	0.1341544000
47	H	-4.9807855000	-0.5037533000	-0.7846164000
48	H	-6.7685189000	0.3422201000	-2.3054966000
49	H	-7.0768227000	2.7927538000	-2.6186358000
50	H	-5.6090967000	4.3965727000	-1.4033212000
51	C	5.6159475000	1.1267659000	-0.8232562000
52	C	6.2477633000	2.1163753000	-1.5765242000
53	C	5.5611796000	3.2822505000	-1.9188444000
54	C	4.2372822000	3.4610030000	-1.5140311000
55	C	3.5956543000	2.4730085000	-0.7698133000
56	H	2.5579347000	2.5930051000	-0.4545887000
57	H	6.1431660000	0.2144214000	-0.5413961000
58	H	7.2828455000	1.9758766000	-1.8923090000
59	H	6.0611397000	4.0563128000	-2.5039113000
60	H	3.6971150000	4.3700392000	-1.7834780000

Table S43. Energy, thermodynamic quantities, and corresponding xyz coordinates (in Angstroms; calculated with ωB97XD density functional and aug-cc-pVDZ basis set) of 4-methoxystyrene N-methylmaleimide ene product.

Energy = -1221.587753 hartrees
Total enthalpy = 249.59 kcal/mol
Total entropy = 162.38 cal/mol×K

ATOM		X	Y	Z
1	C	-0.0510124000	-0.4593595000	0.2197081000
2	C	-1.1420926000	-1.2568031000	-0.1265773000
3	C	-1.0676984000	-2.6503603000	-0.1736454000
4	C	0.1365028000	-3.2846890000	0.1351157000
5	C	1.2457368000	-2.5037722000	0.4793589000
6	C	1.1590612000	-1.1167537000	0.5235256000
7	C	-0.0845750000	1.0601928000	0.3036608000
8	C	0.7160042000	1.4993691000	1.5379389000
9	C	2.1827303000	1.0824456000	1.4301791000
10	C	2.9366201000	2.0107436000	0.4909911000
11	C	2.4075799000	-0.3235652000	0.8428163000
12	C	3.2342601000	-0.0874510000	-0.4222870000
13	H	3.0485486000	-0.9246283000	1.5041008000
14	H	0.4177717000	1.4648672000	-0.5943769000
15	H	-2.1056628000	-0.8122302000	-0.3731265000
16	H	-1.9577048000	-3.2173109000	-0.4432845000
17	H	2.1855582000	-3.0067924000	0.7139800000
18	O	3.0328636000	3.2173439000	0.5764946000
19	N	3.5081043000	1.2649826000	-0.5225402000
20	O	3.6097623000	-0.9252644000	-1.2128030000

21	H	2.6596601000	1.1754568000	2.4159310000
22	H	0.2715992000	1.0480398000	2.4385435000
23	H	0.6700083000	2.5919175000	1.6584460000
24	O	0.3314893000	-4.6256615000	0.1306472000
25	C	-0.7632640000	-5.4523058000	-0.2303061000
26	H	-0.3910117000	-6.4823166000	-0.1901604000
27	H	-1.1129089000	-5.2296703000	-1.2517598000
28	H	-1.6031354000	-5.3394682000	0.4749511000
29	C	-1.4705252000	1.7174413000	0.3208973000
30	C	-2.4552454000	1.3073247000	1.4210812000
31	C	-2.2568483000	1.5507741000	-0.9690547000
32	C	-3.7918450000	1.1820799000	0.7226948000
33	N	-3.5796634000	1.3040826000	-0.6455206000
34	O	-4.8767533000	0.9904918000	1.2287690000
35	O	-1.8410136000	1.6427434000	-2.1053213000
36	H	-2.5312509000	2.0328301000	2.2405758000
37	H	-1.2931836000	2.8039149000	0.3868723000
38	C	-4.6229074000	1.1676801000	-1.6426070000
39	C	4.2893669000	1.8561222000	-1.5917173000
40	H	-5.5643133000	0.9797001000	-1.1144403000
41	H	-4.3956155000	0.3266318000	-2.3117638000
42	H	-4.7057730000	2.0892259000	-2.2342254000
43	H	4.6979247000	1.0406958000	-2.1986956000
44	H	5.1064995000	2.4540286000	-1.1676178000
45	H	3.6542449000	2.5004553000	-2.2148395000
46	H	-2.2122204000	0.3292902000	1.8643854000

Table S44. Energy, thermodynamic quantities, and corresponding xyz coordinates (in Angstroms; calculated with ωB97XD density functional and aug-cc-pVDZ basis set) of 4-methoxystyrene.

Energy = -424.082755 hartrees
Total enthalpy = 111.20 kcal/mol
Total entropy = 92.55 cal/mol×K

ATOM		X	Y	Z
1	C	1.4442341000	0.2425704000	0.0007160000
2	C	0.5030617000	1.2773889000	-0.0017196000
3	C	-0.8718314000	1.0317919000	-0.0042531000
4	C	-1.3343239000	-0.2869189000	-0.0025394000
5	C	-0.4044996000	-1.3401471000	0.0009596000
6	C	0.9553044000	-1.0779910000	0.0028376000
7	C	2.8825134000	0.5718219000	0.0034772000
8	C	3.9077294000	-0.2887645000	-0.0021823000
9	H	3.1029148000	1.6453294000	0.0110602000
10	H	3.7664005000	-1.3729225000	-0.0106766000
11	H	4.9369861000	0.0759906000	0.0023049000
12	H	0.8497783000	2.3141179000	-0.0026462000
13	H	-0.7780378000	-2.3657420000	0.0028545000
14	H	1.6488464000	-1.9213230000	0.0074131000
15	H	-1.5631831000	1.8739481000	-0.0073838000

16	O	-2.6389714000	-0.6498961000	-0.0060321000
17	C	-3.6154234000	0.3790289000	0.0058673000
18	H	-4.5881263000	-0.1259162000	0.0010148000
19	H	-3.5337539000	1.0214172000	-0.8862900000
20	H	-3.5306419000	1.0015879000	0.9116264000

Table S45. Energy, thermodynamic quantities, and corresponding xyz coordinates (in Angstroms; calculated with ωB97XD density functional and aug-cc-pVDZ basis set) of 4-methoxystyrene maleimide first transition state.

Energy = -783.442211 hartrees
Total enthalpy = 159.06 kcal/mol
Total entropy = 119.45 cal/mol×K

ATOM		X	Y	Z
1	O	3.7697000000	-0.0250000000	-0.5395600000
2	C	2.5887900000	0.3901100000	-0.0528500000
3	C	2.1043100000	0.0396400000	1.2349200000
4	C	1.8424400000	1.2342800000	-0.8744800000
5	C	0.8795300000	0.5036500000	1.6481500000
6	C	-2.2305000000	2.0186200000	0.2215300000
7	C	-1.2718900000	1.5800900000	1.1573700000
8	C	0.0665000000	1.3150100000	0.8058400000
9	C	0.5876600000	1.6741500000	-0.4688700000
10	H	2.6875500000	-0.6008200000	1.8948100000
11	H	2.2500700000	1.5182300000	-1.8450700000
12	H	0.4984100000	0.2174100000	2.6311200000
13	H	-3.2330400000	2.2072400000	0.6154200000
14	H	-1.9244000000	2.7442200000	-0.5371400000
15	H	-1.6185400000	1.2100800000	2.1265700000
16	H	0.0563600000	2.3908300000	-1.0921500000
17	O	0.2454600000	-1.5742200000	-1.4631300000
18	N	-1.5769700000	-1.3319800000	-0.0410200000
19	O	-3.6608100000	-0.7627500000	0.7634200000
20	C	-0.7490500000	-0.9389900000	-1.1317700000
21	C	-2.7066700000	-0.5601000000	0.0325400000
22	C	-2.4958200000	0.5891000000	-0.9352400000
23	C	-1.3528200000	0.2436900000	-1.6924400000
24	H	-3.3899200000	1.0007400000	-1.4098900000
25	H	-1.0161600000	0.6999800000	-2.6186200000
26	C	4.5795900000	-0.8802000000	0.2601800000
27	H	5.4745700000	-1.0859200000	-0.3369500000
28	H	4.0582800000	-1.8248700000	0.4803100000
29	H	4.8716600000	-0.3875800000	1.2006300000
30	H	-1.4137100000	-2.1733600000	0.5310900000

Table S46. Energy, thermodynamic quantities, and corresponding xyz coordinates (in Angstroms; calculated with ωB97XD density functional and aug-cc-pVDZ basis set) of 4-methoxystyrene N-phenylmaleimide first transition state.

Energy = -1014.437717 hartrees
Total enthalpy = 212.69 kcal/mol
Total entropy = 142.14 cal/mol×K

ATOM		X	Y	Z
1	O	-3.7781114000	-1.5367672000	0.4445513000
2	C	-2.9321573000	-0.6721216000	-0.1355839000
3	C	-2.0161117000	-1.0365547000	-1.1571080000
4	C	-3.0110737000	0.6535551000	0.2933231000
5	C	-1.1780742000	-0.0866825000	-1.6869129000
6	C	0.1372627000	3.3230525000	-0.8807943000
7	C	-0.1573553000	2.1439501000	-1.5963646000
8	C	-1.1843797000	1.2584196000	-1.2167948000
9	C	-2.1364149000	1.6040711000	-0.2160015000
10	H	-1.9632718000	-2.0625007000	-1.5182159000
11	H	-3.7485751000	0.9172359000	1.0517422000
12	H	-0.4603049000	-0.3719305000	-2.4589815000
13	H	0.9498135000	3.9354719000	-1.2813557000
14	H	-0.6951544000	3.9157422000	-0.4912002000
15	H	0.5693690000	1.7829624000	-2.3300875000
16	H	-2.2603845000	2.6436411000	0.0814353000
17	O	-0.4251716000	-0.2992231000	2.0031746000
18	N	1.3275637000	0.5239741000	0.6897223000
19	O	2.9206032000	1.9817979000	-0.1553883000
20	C	0.1412464000	0.6590153000	1.4956673000
21	C	1.8451956000	1.7667320000	0.3680095000
22	C	0.7937301000	2.7765902000	0.7696308000
23	C	-0.1422934000	2.0660781000	1.5554522000
24	H	1.1599594000	3.7608184000	1.0711108000
25	H	-0.9146795000	2.4838251000	2.1941790000
26	C	-3.7497924000	-2.9024327000	0.0416662000
27	H	-4.5057092000	-3.4075857000	0.6525107000
28	H	-2.7618933000	-3.3494653000	0.2329399000
29	H	-4.0073351000	-3.0084680000	-1.0235669000
30	C	1.8975070000	-0.7131309000	0.2965215000
31	C	2.2640288000	-0.9076393000	-1.0393880000
32	C	2.8151745000	-2.1234205000	-1.4376217000
33	C	2.9884373000	-3.1547544000	-0.5134963000
34	C	2.6167016000	-2.9581090000	0.8168129000
35	C	2.0794931000	-1.7386816000	1.2282481000
36	H	1.7956549000	-1.5815966000	2.2677395000
37	H	2.1166176000	-0.1059665000	-1.7631783000
38	H	3.1018236000	-2.2664279000	-2.4811360000
39	H	2.7526689000	-3.7582318000	1.5469125000
40	H	3.4131474000	-4.1094252000	-0.8291975000

Table S47. Energy, thermodynamic quantities, and corresponding xyz coordinates (in Angstroms; calculated with ωB97XD density functional and aug-cc-pVDZ basis set) of 4-methoxystyrene N-methylmaleimide first transition state.

Energy = -822.744147 hartrees
Total enthalpy = 177.70 kcal/mol
Total entropy = 128.02 cal/mol×K

ATOM		X	Y	Z
1	O	3.7697002000	-0.0249992000	-0.5395643000
2	C	2.5887862000	0.3901087000	-0.0528479000
3	C	2.1043115000	0.0396393000	1.2349216000
4	C	1.8424384000	1.2342772000	-0.8744781000
5	C	0.8795342000	0.5036452000	1.6481533000
6	C	-2.2304958000	2.0186186000	0.2215305000
7	C	-1.2718892000	1.5800866000	1.1573744000
8	C	0.0664991000	1.3150065000	0.8058448000
9	C	0.5876598000	1.6741452000	-0.4688730000
10	H	2.6875503000	-0.6008244000	1.8948112000
11	H	2.2500698000	1.5182295000	-1.8450735000
12	H	0.4984055000	0.2174050000	2.6311213000
13	H	-3.2330441000	2.2072389000	0.6154236000
14	H	-1.9243966000	2.7442241000	-0.5371433000
15	H	-1.6185397000	1.2100800000	2.1265710000
16	H	0.0563646000	2.3908288000	-1.0921488000
17	O	0.2454583000	-1.5742165000	-1.4631282000
18	N	-1.5769659000	-1.3319771000	-0.0410230000
19	O	-3.6608070000	-0.7627488000	0.7634171000
20	C	-0.7490498000	-0.9389928000	-1.1317664000
21	C	-2.7066682000	-0.5601046000	0.0325407000
22	C	-2.4958229000	0.5890969000	-0.9352415000
23	C	-1.3528201000	0.2436944000	-1.6924395000
24	H	-3.3899171000	1.0007431000	-1.4098896000
25	H	-1.0161558000	0.6999752000	-2.6186242000
26	C	4.5795882000	-0.8802042000	0.2601752000
27	H	5.4745700000	-1.0859231000	-0.3369541000
28	H	4.0582832000	-1.8248656000	0.4803129000
29	H	4.8716614000	-0.3875771000	1.2006284000
30	C	-1.3101234000	-2.4717066000	0.8034582000
31	H	-0.4994140000	-3.0462283000	0.3396122000
32	H	-2.2084829000	-3.0973862000	0.8943576000
33	H	-0.9946935000	-2.1502272000	1.8082459000

Table S48. Energy, thermodynamic quantities, and corresponding xyz coordinates (in Angstroms; calculated with ωB97XD density functional and aug-cc-pVDZ basis set) of 4-methoxystyrene maleimide second transition state for Diels−Alder mechanism.

Energy = -1142.869107 hartrees
Total enthalpy = 209.42 kcal/mol
Total entropy = 145.35 cal/mol×K

ATOM		X	Y	Z
1	C	-0.7306100000	1.2504200000	0.3728900000

2	C	0.2230300000	0.7866100000	1.3670600000
3	C	0.7992800000	-0.4576200000	1.2775500000
4	C	0.8361500000	-1.1071300000	0.0182000000
5	C	0.4268600000	-0.3953500000	-1.1158100000
6	C	-0.7066900000	0.5942000000	-0.9879500000
7	C	-1.6961700000	2.1540000000	0.6204700000
8	C	-2.7273100000	2.3872800000	-0.4445000000
9	C	-3.2311300000	1.0251800000	-0.9688600000
10	C	-4.0444800000	0.3253000000	0.1083800000
11	C	-2.1188700000	0.0185300000	-1.3233000000
12	C	-2.4577500000	-1.2281100000	-0.5219500000
13	H	-2.1324900000	-0.2586300000	-2.3861100000
14	H	-0.5622000000	1.3814100000	-1.7472400000
15	H	-1.7746300000	2.6488000000	1.5912600000
16	H	0.2956500000	1.3448000000	2.3034500000
17	H	1.3356500000	-0.8637600000	2.1340700000
18	H	0.5084500000	-0.9223400000	-2.0693900000
19	O	-4.9837800000	0.7776100000	0.7297700000
20	N	-3.5465900000	-0.9507500000	0.2877800000
21	O	-1.8998100000	-2.3050800000	-0.5640600000
22	H	-3.8982600000	1.1881900000	-1.8276100000
23	H	-2.2975600000	2.9477200000	-1.2928900000
24	H	-3.5801800000	2.9658600000	-0.0674100000
25	O	1.4305000000	-2.2903300000	-0.1977400000
26	C	2.0603300000	-2.9487500000	0.8956200000
27	H	2.4837100000	-3.8705500000	0.4824600000
28	H	1.3297000000	-3.1975700000	1.6808100000
29	H	2.8674800000	-2.3322200000	1.3193900000
30	H	-3.9524700000	-1.6315000000	0.9466700000
31	C	2.0278300000	1.8933900000	-0.2644000000
32	C	1.9840300000	0.9793500000	-1.3144000000
33	C	3.0972300000	1.5040700000	0.6475600000
34	C	3.1598500000	0.0573800000	-1.1389000000
35	N	3.6733200000	0.3315200000	0.1129900000
36	O	3.5853300000	-0.7803700000	-1.9110700000
37	O	3.5095700000	2.0298200000	1.6700200000
38	H	1.6476600000	1.2124500000	-2.3245900000
39	H	1.5008800000	2.8390800000	-0.1827200000
40	H	4.4794100000	-0.1578500000	0.5285300000

Table S49. Energy, thermodynamic quantities, and corresponding xyz coordinates (in Angstroms; calculated with ωB97XD density functional and aug-cc-pVDZ basis set) of 4-methoxystyrene N-phenylmaleimide second transition state for Diels−Alder mechanism.

Energy = -1604.856624 hartrees
Total enthalpy = 317.15 kcal/mol
Total entropy = 185.46 cal/mol×K

ATOM		X	Y	Z
1	C	-0.8690336000	1.6104016000	0.8833095000

2	C	-0.0494963000	0.5513349000	1.4473586000
3	C	0.4623483000	-0.4541502000	0.6650737000
4	C	0.5621568000	-0.2510385000	-0.7354907000
5	C	0.2939918000	1.0239413000	-1.2514698000
6	C	-0.7830832000	1.8586041000	-0.6039672000
7	C	-1.7887740000	2.3024109000	1.5814320000
8	C	-2.7071162000	3.2062980000	0.8129439000
9	C	-3.2684924000	2.4399269000	-0.4086020000
10	C	-4.1909326000	1.3356856000	0.0850417000
11	C	-2.2000714000	1.7116347000	-1.2476414000
12	C	-2.6555344000	0.2646396000	-1.2868403000
13	H	-2.1514107000	2.0847216000	-2.2790542000
14	H	-0.5376232000	2.9216236000	-0.7641050000
15	H	-1.9202764000	2.1521415000	2.6553307000
16	H	-0.0268181000	0.4496379000	2.5349013000
17	H	0.9008075000	-1.3327009000	1.1365526000
18	H	0.4117210000	1.1374213000	-2.3316785000
19	O	-5.1385544000	1.4501821000	0.8293306000
20	N	-3.7522833000	0.1256165000	-0.4417654000
21	O	-2.1686504000	-0.6485487000	-1.9155429000
22	H	-3.8641733000	3.1233903000	-1.0296356000
23	H	-2.1677245000	4.0952307000	0.4424998000
24	H	-3.5418555000	3.5627485000	1.4293634000
25	O	1.0821979000	-1.1429904000	-1.5871060000
26	C	1.5448367000	-2.3912324000	-1.0784131000
27	H	1.8916535000	-2.9553873000	-1.9506591000
28	H	0.7286804000	-2.9398880000	-0.5837472000
29	H	2.3813535000	-2.2470927000	-0.3781910000
30	C	-4.2727319000	-1.1466232000	-0.0510809000
31	C	-5.3808093000	-1.6952103000	-0.6917028000
32	C	-5.8537180000	-2.9420281000	-0.2805813000
33	C	-5.2208062000	-3.6260219000	0.7585784000
34	C	-4.1104205000	-3.0658963000	1.3929113000
35	C	-3.6328931000	-1.8215803000	0.9883646000
36	H	-2.7667338000	-1.3646912000	1.4708594000
37	H	-5.5942711000	-4.6017591000	1.0746053000
38	H	-3.6143053000	-3.5983002000	2.2060057000
39	H	-5.8632491000	-1.1501271000	-1.5042917000
40	H	-6.7199425000	-3.3818089000	-0.7777525000
41	C	1.9997525000	2.2201263000	0.7996915000
42	C	1.9284161000	2.1072867000	-0.5862223000
43	C	2.9929205000	1.2875558000	1.3003702000
44	C	3.0165507000	1.1672396000	-1.0127727000
45	N	3.5209657000	0.6057114000	0.1580842000
46	O	3.3802662000	0.9139551000	-2.1424124000
47	O	3.3921980000	1.0858063000	2.4337764000
48	H	1.6573665000	2.9195590000	-1.2604953000
49	H	1.5400021000	2.9748471000	1.4300274000
50	C	4.5732180000	-0.3460098000	0.2113369000
51	C	5.6737729000	-0.2183037000	-0.6408894000
52	C	6.6928990000	-1.1680783000	-0.6026159000

53	C	6.6335706000	-2.2313755000	0.2980467000
54	C	5.5425729000	-2.3436276000	1.1613326000
55	C	4.5087672000	-1.4091421000	1.1183399000
56	H	3.6493824000	-1.5024771000	1.7827054000
57	H	7.4351588000	-2.9716621000	0.3275370000
58	H	5.4892661000	-3.1699851000	1.8726548000
59	H	5.7270878000	0.6155593000	-1.3386511000
60	H	7.5443035000	-1.0681370000	-1.2784279000

Table S50. Energy, thermodynamic quantities, and corresponding xyz coordinates (in Angstroms; calculated with ωB97XD density functional and aug-cc-pVDZ basis set) of 4-methoxystyrene N-methylmaleimide second transition state for Diels−Alder mechanism.

Energy = -1221.472814 hartrees
Total enthalpy = 246.52 kcal/mol
Total entropy = 160.14 cal/mol×K

ATOM		X	Y	Z
1	C	-0.7298448000	1.2433103000	0.4069056000
2	C	0.2244807000	0.7705450000	1.3959298000
3	C	0.8103232000	-0.4678411000	1.2905753000
4	C	0.8551712000	-1.1031965000	0.0247004000
5	C	0.4380062000	-0.3817218000	-1.1008962000
6	C	-0.6991645000	0.6005398000	-0.9595827000
7	C	-1.7048024000	2.1362856000	0.6577481000
8	C	-2.7258032000	2.3793670000	-0.4144233000
9	C	-3.2237944000	1.0284777000	-0.9762692000
10	C	-4.0806235000	0.3235062000	0.0629146000
11	C	-2.1046044000	0.0182141000	-1.2989121000
12	C	-2.4577253000	-1.2209787000	-0.4920619000
13	H	-2.1077484000	-0.2718157000	-2.3587939000
14	H	-0.5604914000	1.3957105000	-1.7116729000
15	H	-1.7958330000	2.6196554000	1.6332587000
16	H	0.2939817000	1.3171885000	2.3392269000
17	H	1.3517185000	-0.8769406000	2.1425324000
18	H	0.5168351000	-0.8998067000	-2.0596005000
19	O	-5.0509453000	0.7704036000	0.6391049000
20	N	-3.5793947000	-0.9464956000	0.2723119000
21	O	-1.8855953000	-2.2913994000	-0.4956160000
22	H	-3.8582945000	1.2117524000	-1.8549635000
23	H	-2.2863739000	2.9571054000	-1.2462831000
24	H	-3.5845449000	2.9507574000	-0.0393245000
25	O	1.4524755000	-2.2814605000	-0.2056824000
26	C	2.0877006000	-2.9495852000	0.8788894000
27	H	2.4976448000	-3.8741399000	0.4586124000
28	H	1.3639622000	-3.1937631000	1.6719106000
29	H	2.9048090000	-2.3407169000	1.2952530000
30	C	-4.1872361000	-1.8929722000	1.1868252000
31	H	-3.5693845000	-2.7975215000	1.1923213000
32	H	-5.2052548000	-2.1389249000	0.8552853000

33	H	-4.2309157000	-1.4616601000	2.1957180000
34	C	2.0354531000	1.8934742000	-0.2350055000
35	C	1.9799115000	0.9962625000	-1.3002928000
36	C	3.1190009000	1.4932979000	0.6538574000
37	C	3.1602635000	0.0740969000	-1.1546907000
38	N	3.6872692000	0.3273986000	0.0954059000
39	O	3.5782715000	-0.7495999000	-1.9457593000
40	O	3.5455817000	2.0048076000	1.6779816000
41	H	1.6308476000	1.2440555000	-2.3026342000
42	H	1.5105899000	2.8385027000	-0.1345354000
43	C	4.8440313000	-0.3419598000	0.6461371000
44	H	4.9619862000	-1.3011993000	0.1277105000
45	H	4.6912926000	-0.5181535000	1.7188146000
46	H	5.7572854000	0.2568509000	0.5088198000

Table S51. Energy, thermodynamic quantities, and corresponding xyz coordinates (in Angstroms; calculated with ωB97XD density functional and aug-cc-pVDZ basis set) of 4-methoxystyrene maleimide second transition state for ene mechanism.

Energy = -1142.862569 hartrees
Total enthalpy = 207.97 kcal/mol
Total entropy = 145.51 cal/mol×K

ATOM		X	Y	Z
1	C	0.1398100000	0.4533600000	-0.7081300000
2	C	-0.5190700000	-0.3259600000	-1.7076600000
3	C	-0.9823200000	-1.5797200000	-1.4383300000
4	C	-0.8419100000	-2.1524800000	-0.1226900000
5	C	-0.1815900000	-1.4724500000	0.8533200000
6	C	0.3213700000	-0.1116700000	0.6369800000
7	C	0.3719300000	1.8235300000	-0.8344000000
8	C	1.3953300000	2.4809500000	0.0735800000
9	C	2.3497800000	1.4578700000	0.6849100000
10	C	3.3518500000	0.9163000000	-0.3230100000
11	C	1.7049900000	0.1991400000	1.2812400000
12	C	2.6876100000	-0.9095800000	0.9143800000
13	H	1.6036600000	0.2361500000	2.3726700000
14	H	0.1995000000	2.2798300000	-1.8122500000
15	H	-0.6447900000	0.0990900000	-2.7048400000
16	H	-1.4634800000	-2.1483700000	-2.2337200000
17	H	-0.0274300000	-1.9442900000	1.8241200000
18	O	3.9411600000	1.5334600000	-1.1848100000
19	N	3.5336500000	-0.4313100000	-0.0724600000
20	O	2.7306900000	-2.0339300000	1.3653400000
21	H	2.9559600000	1.9558400000	1.4581800000
22	H	0.9268600000	3.0404800000	0.8950300000
23	H	1.9825800000	3.2081200000	-0.5050600000
24	O	-1.3076100000	-3.3916800000	0.1871800000
25	C	-2.0937500000	-4.0855500000	-0.7691600000
26	H	-2.3998200000	-5.0186700000	-0.2821100000

27	H	-1.5161300000	-4.3266700000	-1.6761600000
28	H	-2.9923700000	-3.5101700000	-1.0460100000
29	H	-0.4045000000	0.6395400000	1.2431300000
30	H	4.2329500000	-1.0129500000	-0.5570900000
31	C	-1.4192200000	2.5053700000	0.1388000000
32	C	-1.4953600000	1.9437800000	1.4119500000
33	C	-2.4674900000	1.8392300000	-0.6981200000
34	C	-2.5451300000	0.9070800000	1.4008000000
35	N	-3.0358800000	0.8539900000	0.0902300000
36	O	-2.9316500000	0.1847500000	2.3012100000
37	O	-2.7727100000	2.0614200000	-1.8537100000
38	H	-1.1129600000	2.3604300000	2.3417700000
39	H	-1.1249400000	3.5281700000	-0.0877000000
40	H	-3.7803700000	0.2151000000	-0.2252100000

Table S52. Energy, thermodynamic quantities, and corresponding xyz coordinates (in Angstroms; calculated with ωB97XD density functional and aug-cc-pVDZ basis set) of 4-methoxystyrene N-phenylmaleimide second transition state for ene mechanism.

Energy = -1604.852899 hartrees
Total enthalpy = 315.50 kcal/mol
Total entropy = 189.33 cal/mol×K

ATOM		X	Y	Z
1	C	0.1398100000	0.4533600000	-0.7081300000
2	C	-0.5190700000	-0.3259600000	-1.7076600000
3	C	-0.9823200000	-1.5797200000	-1.4383300000
4	C	-0.8419100000	-2.1524800000	-0.1226900000
5	C	-0.1815900000	-1.4724500000	0.8533200000
6	C	0.3213700000	-0.1116700000	0.6369800000
7	C	0.3719300000	1.8235300000	-0.8344000000
8	C	1.3953300000	2.4809500000	0.0735800000
9	C	2.3497800000	1.4578700000	0.6849100000
10	C	3.3518500000	0.9163000000	-0.3230100000
11	C	1.7049900000	0.1991400000	1.2812400000
12	C	2.6876100000	-0.9095800000	0.9143800000
13	H	1.6036600000	0.2361500000	2.3726700000
14	H	0.1995000000	2.2798300000	-1.8122500000
15	H	-0.6447900000	0.0990900000	-2.7048400000
16	H	-1.4634800000	-2.1483700000	-2.2337200000
17	H	-0.0274300000	-1.9442900000	1.8241200000
18	O	3.9411600000	1.5334600000	-1.1848100000
19	N	3.5336500000	-0.4313100000	-0.0724600000
20	O	2.7306900000	-2.0339300000	1.3653400000
21	H	2.9559600000	1.9558400000	1.4581800000
22	H	0.9268600000	3.0404800000	0.8950300000
23	H	1.9825800000	3.2081200000	-0.5050600000
24	O	-1.3076100000	-3.3916800000	0.1871800000
25	C	-2.0937500000	-4.0855500000	-0.7691600000
26	H	-2.3998200000	-5.0186700000	-0.2821100000

27	H	-1.5161300000	-4.3266700000	-1.6761600000
28	H	-2.9923700000	-3.5101700000	-1.0460100000
29	H	-0.4045000000	0.6395400000	1.2431300000
30	C	4.5109800000	-1.2554900000	-0.7589700000
31	C	4.3132200000	-2.6506500000	-0.9096700000
32	C	5.2555600000	-3.4406100000	-1.5742100000
33	C	6.4110000000	-2.8658900000	-2.0979500000
34	C	6.6295100000	-1.4971300000	-1.9618200000
35	C	5.6934700000	-0.6947900000	-1.3029100000
36	H	5.9202800000	0.3541900000	-1.2051400000
37	H	7.1383300000	-3.4823100000	-2.6104000000
38	H	7.5308300000	-1.0536100000	-2.3652600000
39	H	3.4250200000	-3.1388600000	-0.5421800000
40	H	5.0851700000	-4.5037600000	-1.6860800000
41	C	-1.4192200000	2.5053700000	0.1388000000
42	C	-1.4953600000	1.9437800000	1.4119500000
43	C	-2.4674900000	1.8392300000	-0.6981200000
44	C	-2.5451300000	0.9070800000	1.4008000000
45	N	-3.0358800000	0.8539900000	0.0902300000
46	O	-2.9316500000	0.1847500000	2.3012100000
47	O	-2.7727100000	2.0614200000	-1.8537100000
48	H	-1.1129600000	2.3604300000	2.3417700000
49	H	-1.1249400000	3.5281700000	-0.0877000000
50	C	-4.0814300000	-0.0571600000	-0.3475600000
51	C	-4.2900200000	-1.2982200000	0.3061500000
52	C	-5.2965700000	-2.1723400000	-0.1132500000
53	C	-6.1167900000	-1.8376300000	-1.1861100000
54	C	-5.9349100000	-0.6262500000	-1.8465300000
55	C	-4.9313700000	0.2575900000	-1.4393700000
56	H	-4.8566500000	1.1873400000	-1.9741800000
57	H	-3.6699800000	-1.6214600000	1.1245800000
58	H	-5.4350700000	-3.1180700000	0.3949800000
59	H	-6.8947800000	-2.5184200000	-1.5066500000
60	H	-6.5779200000	-0.3656500000	-2.6775300000

Table S53. Energy, thermodynamic quantities, and corresponding xyz coordinates (in Angstroms; calculated with ωB97XD density functional and aug-cc-pVDZ basis set) of 4-methoxystyrene N-methylmaleimide second transition state for ene mechanism.

Energy = -1221.468689 hartrees
Total enthalpy = 245.21 kcal/mol
Total entropy = 161.05 cal/mol×K

ATOM		X	Y	Z
1	C	0.1542293000	0.4542798000	-0.7047972000
2	C	-0.4819510000	-0.3327576000	-1.7117964000
3	C	-0.9617801000	-1.5793292000	-1.4392362000
4	C	-0.8663913000	-2.1331194000	-0.1116007000
5	C	-0.2168192000	-1.4497811000	0.8699189000
6	C	0.3141858000	-0.1004521000	0.6472222000

7	C	0.3960847000	1.8226983000	-0.8367293000
8	C	1.4163717000	2.4747374000	0.0789084000
9	C	2.3586334000	1.4423680000	0.6953577000
10	C	3.3457356000	0.8850182000	-0.3192603000
11	C	1.7002804000	0.1915480000	1.2942803000
12	C	2.6686035000	-0.9302694000	0.9267526000
13	H	1.5992572000	0.2297537000	2.3856606000
14	H	0.2470668000	2.2702551000	-1.8224924000
15	H	-0.5762754000	0.0803646000	-2.7174055000
16	H	-1.4239353000	-2.1561528000	-2.2398170000
17	H	-0.0955967000	-1.9091669000	1.8514851000
18	O	3.9372270000	1.4937522000	-1.1855579000
19	N	3.5084529000	-0.4652111000	-0.0715409000
20	O	2.7024786000	-2.0525362000	1.3830539000
21	H	2.9739095000	1.9338765000	1.4654645000
22	H	0.9448465000	3.0356847000	0.8975721000
23	H	2.0128100000	3.1984498000	-0.4942422000
24	O	-1.3680195000	-3.3562198000	0.2060220000
25	C	-2.1560182000	-4.0408185000	-0.7556248000
26	H	-2.5197450000	-4.9451198000	-0.2540515000
27	H	-1.5624911000	-4.3333887000	-1.6368960000
28	H	-3.0183086000	-3.4343602000	-1.0774868000
29	C	4.4679907000	-1.2892403000	-0.7805650000
30	H	4.2887688000	-2.3338025000	-0.5031315000
31	H	5.4936606000	-1.0055776000	-0.5069904000
32	H	4.3329483000	-1.1583854000	-1.8619906000
33	C	-1.3975460000	2.5205890000	0.1008487000
34	C	-1.4798478000	1.9891309000	1.3879751000
35	C	-2.4364870000	1.8257341000	-0.7259991000
36	C	-2.5251772000	0.9498011000	1.3966175000
37	N	-2.9999840000	0.8545337000	0.0823903000
38	O	-2.9188650000	0.2516690000	2.3131472000
39	O	-2.7373515000	2.0172560000	-1.8881438000
40	H	-1.1045547000	2.4300894000	2.3094130000
41	H	-1.1097626000	3.5407057000	-0.1463948000
42	C	-3.9607888000	-0.1229635000	-0.3716954000
43	H	-4.1783900000	-0.7897738000	0.4709574000
44	H	-3.5380055000	-0.7073910000	-1.2027721000
45	H	-4.8872572000	0.3638624000	-0.7071875000
46	H	-0.3978366000	0.6704051000	1.2427260000

Table S54. Energy, thermodynamic quantities, and corresponding xyz coordinates (in Angstroms; calculated with ωB97XD density functional and aug-cc-pVDZ basis set) of 4-methoxystyrene maleic anhydride intermediate in DMSO (ε = 41.8).

Energy = -803.344933 hartrees
Total enthalpy = 152.86 kcal/mol
Total entropy = 118.08 cal/mol×K

ATOM X Y Z

1	C	-0.0439355000	-1.3560917000	0.1669521000
2	C	-1.1034370000	-1.5318987000	1.1525735000
3	C	-2.2553881000	-0.8383922000	1.0762033000
4	C	-2.4861832000	0.1195322000	-0.0100807000
5	C	-1.5590146000	0.3551903000	-0.9493254000
6	C	-0.2419388000	-0.3617839000	-0.9654341000
7	C	1.1368636000	-2.0044300000	0.2180229000
8	C	2.1791772000	-1.6792248000	-0.8109527000
9	C	2.3214225000	-0.1474782000	-0.9497185000
10	C	2.9868721000	0.4249655000	0.2839366000
11	C	0.9839444000	0.5997924000	-1.0335817000
12	C	1.0266311000	1.5628506000	0.1334855000
13	H	0.9020679000	1.2042220000	-1.9477305000
14	H	-0.1843422000	-0.9183071000	-1.9206300000
15	H	1.3492339000	-2.7280647000	1.0084348000
16	H	-0.9232952000	-2.2261644000	1.9769059000
17	H	-3.0272017000	-0.9549424000	1.8392157000
18	O	-3.6588442000	0.8316525000	-0.0103782000
19	H	-1.7610598000	1.0814953000	-1.7390028000
20	O	4.0355317000	0.1334770000	0.7805916000
21	O	2.1966820000	1.4013590000	0.8466014000
22	O	0.2257469000	2.3826090000	0.4743449000
23	H	2.9716701000	0.0867537000	-1.8043029000
24	H	1.8838565000	-2.0745956000	-1.7989382000
25	H	3.1559070000	-2.1147143000	-0.5646910000
26	C	-4.8311570000	0.0410641000	-0.1687805000
27	H	-5.6825423000	0.7323093000	-0.1457696000
28	H	-4.9454179000	-0.6960502000	0.6435117000
29	H	-4.8149482000	-0.4892938000	-1.1360816000

Table S55. Energy, thermodynamic quantities, and corresponding xyz coordinates (in Angstroms; calculated with ωB97XD density functional and aug-cc-pVDZ basis set) of 4-methoxystyrene maleic anhydride double-Diels−Alder product in DMSO (ε = 41.8).

Energy = -1182.650121 hartrees
Total enthalpy = 196.30 kcal/mol
Total entropy = 136.87 cal/mol×K

ATOM		X	Y	Z
1	C	0.5743977000	-1.4387458000	0.0810321000
2	C	-0.6842221000	-1.2850170000	0.9014737000
3	C	-0.7486737000	0.1578906000	1.3683725000
4	C	-0.7247576000	1.0431410000	0.3543974000
5	C	-0.6113992000	0.4392928000	-1.0262200000
6	C	0.6184557000	-0.5016339000	-1.1102124000
7	C	1.5803328000	-2.2844938000	0.3121623000
8	C	2.7991411000	-2.2148813000	-0.5687647000
9	C	3.1599011000	-0.7431907000	-0.8108262000
10	C	3.6006162000	-0.0816855000	0.4802073000
11	C	2.0134944000	0.1604365000	-1.2988828000

12	C	2.2215335000	1.4275534000	-0.4958899000
13	H	2.1198735000	0.4385135000	-2.3548010000
14	H	0.4879632000	-1.1142268000	-2.0180795000
15	H	1.5489712000	-2.9860353000	1.1491914000
16	H	-0.7155632000	-1.9954740000	1.7348239000
17	H	-0.8402848000	0.4074104000	2.4238921000
18	O	-0.8544714000	2.3792801000	0.3914509000
19	H	-0.5935388000	1.2139174000	-1.8012164000
20	O	4.3108228000	-0.5018071000	1.3456845000
21	O	3.0758213000	1.1890573000	0.5607607000
22	O	1.7875875000	2.5249624000	-0.6848019000
23	H	4.0166253000	-0.6696674000	-1.4990193000
24	H	2.6152166000	-2.6965624000	-1.5433836000
25	H	3.6567730000	-2.7250588000	-0.1112693000
26	C	-0.9466684000	2.9563500000	1.6851394000
27	H	-1.0299634000	4.0386045000	1.5373634000
28	H	-0.0445641000	2.7275824000	2.2758409000
29	H	-1.8364912000	2.5822418000	2.2180193000
30	C	-1.8713204000	-1.5374535000	-0.0744130000
31	C	-1.8406902000	-0.4916961000	-1.1990610000
32	C	-3.2044977000	-1.3130102000	0.5987477000
33	C	-3.1449930000	0.2582855000	-1.0453838000
34	O	-3.8878049000	-0.2859161000	-0.0209563000
35	O	-3.5554285000	1.1957464000	-1.6637104000
36	O	-3.6835167000	-1.8778132000	1.5371307000
37	H	-1.8299321000	-0.9460256000	-2.1997473000
38	H	-1.8430672000	-2.5701519000	-0.4473521000

Table S56. Energy, thermodynamic quantities, and corresponding xyz coordinates (in Angstroms; calculated with ωB97XD density functional and aug-cc-pVDZ basis set) of 4-methoxystyrene maleic anhydride ene product in DMSO (ε = 41.8).

Energy = -1182.679955 hartrees
Total enthalpy = 195.71 kcal/mol
Total entropy = 144.94 cal/mol×K

ATOM		X	Y	Z
1	C	-0.0540000000	-0.4613600000	0.2173200000
2	C	-1.1510600000	-1.2526300000	-0.1239400000
3	C	-1.0848800000	-2.6467000000	-0.1695900000
4	C	0.1168200000	-3.2877000000	0.1351100000
5	C	1.2320200000	-2.5129300000	0.4741000000
6	C	1.1532400000	-1.1254800000	0.5174700000
7	C	-0.0776000000	1.0583900000	0.3009400000
8	C	0.7252500000	1.4920400000	1.5358700000
9	C	2.1896300000	1.0651100000	1.4305100000
10	C	2.9539800000	1.9940000000	0.5005400000
11	C	2.4059300000	-0.3382400000	0.8334700000
12	C	3.2282300000	-0.0981700000	-0.4336900000
13	H	3.0485600000	-0.9447600000	1.4882500000

14	H	0.4276200000	1.4597300000	-0.5969500000
15	H	-2.1129300000	-0.8025800000	-0.3671300000
16	H	-1.9792300000	-3.2088700000	-0.4348500000
17	H	2.1697100000	-3.0211700000	0.7060400000
18	O	3.0647600000	3.1984300000	0.5984300000
19	O	3.5148500000	1.2525100000	-0.5219900000
20	O	3.5920200000	-0.9321100000	-1.2336900000
21	H	2.6630000000	1.1475700000	2.4188400000
22	H	0.2768300000	1.0432800000	2.4357500000
23	H	0.6861700000	2.5848100000	1.6570300000
24	O	0.3038900000	-4.6298000000	0.1316900000
25	C	-0.7982000000	-5.4505500000	-0.2201600000
26	H	-0.4323200000	-6.4827900000	-0.1785600000
27	H	-1.1522100000	-5.2293000000	-1.2404100000
28	H	-1.6333300000	-5.3297800000	0.4893900000
29	C	-1.4594000000	1.7243400000	0.3181400000
30	C	-2.4455000000	1.3219800000	1.4199800000
31	C	-2.2479700000	1.5600500000	-0.9707300000
32	C	-3.7835800000	1.2037900000	0.7231700000
33	O	-3.5720000000	1.3221000000	-0.6454800000
34	O	-4.8691100000	1.0196800000	1.2306700000
35	O	-1.8326900000	1.6472200000	-2.1075700000
36	H	-2.5162200000	2.0492800000	2.2383700000
37	H	-1.2754200000	2.8098200000	0.3821400000
38	H	-2.2081500000	0.3432100000	1.8647400000

Table S57. Energy, thermodynamic quantities, and corresponding xyz coordinates (in Angstroms; calculated with ωB97XD density functional and aug-cc-pVDZ basis set) of 4-methoxystyrene maleic anhydride first transition state in DMSO (ε = 41.8).

Energy = -803.298381 hartrees
Total enthalpy = 150.92 kcal/mol
Total entropy = 118.88 cal/mol×K

ATOM		X	Y	Z
1	O	3.6884738000	-0.2985304000	-0.4639415000
2	C	2.5067711000	0.2707589000	-0.1895348000
3	C	2.0661454000	0.5811698000	1.1176347000
4	C	1.7058666000	0.5834722000	-1.2950286000
5	C	0.8255116000	1.1521762000	1.2969038000
6	C	-2.3795808000	1.7589737000	-0.5168026000
7	C	-1.3765256000	1.8044738000	0.4397420000
8	C	-0.0290956000	1.4245512000	0.1986183000
9	C	0.4545552000	1.1437333000	-1.1059876000
10	H	2.6892296000	0.3667374000	1.9844685000
11	H	2.0827101000	0.3668804000	-2.2947899000
12	H	0.4747716000	1.3684386000	2.3083525000
13	H	-3.3830239000	2.0678821000	-0.2147386000
14	H	-2.1424242000	1.9409811000	-1.5688687000
15	H	-1.6672449000	1.9380798000	1.4863612000

16	H	-0.1319292000	1.4263364000	-1.9791343000
17	O	0.1529925000	-2.2068344000	-0.3804736000
18	O	-1.5944973000	-1.3950184000	0.8197206000
19	O	-3.6183280000	-0.5914821000	1.3623910000
20	C	-0.8437470000	-1.5251230000	-0.3905278000
21	C	-2.7368828000	-0.7067552000	0.5526761000
22	C	-2.6443038000	-0.1741375000	-0.8461375000
23	C	-1.5362898000	-0.7944773000	-1.4096010000
24	H	-3.5769037000	0.0301908000	-1.3776006000
25	H	-1.2230006000	-0.7903837000	-2.4491587000
26	C	4.5623048000	-0.6235131000	0.6129006000
27	H	5.4466280000	-1.0738884000	0.1495513000
28	H	4.0942720000	-1.3493114000	1.2959214000
29	H	4.8576879000	0.2787193000	1.1703653000

Table S58. Energy, thermodynamic quantities, and corresponding xyz coordinates (in Angstroms; calculated with ωB97XD density functional and aug-cc-pVDZ basis set) of 4-methoxystyrene maleic anhydride second transition state for Diels−Alder mechanism in DMSO (ε = 41.8).

Energy = -1182.573189 hartrees
Total enthalpy = 193.19 kcal/mol
Total entropy = 143.50 cal/mol×K

ATOM		X	Y	Z
1	C	-0.7586345000	1.1018810000	0.4444247000
2	C	0.2242347000	0.7901688000	1.4654083000
3	C	0.9272241000	-0.3851126000	1.4583477000
4	C	0.9795055000	-1.1494328000	0.2615951000
5	C	0.4905259000	-0.5854897000	-0.9293880000
6	C	-0.6986229000	0.3452582000	-0.8621981000
7	C	-1.7854903000	1.9507608000	0.6358780000
8	C	-2.8252822000	2.0528761000	-0.4400734000
9	C	-3.2406509000	0.6378568000	-0.9103633000
10	C	-4.0839027000	-0.0350592000	0.1523891000
11	C	-2.0720317000	-0.3354848000	-1.1271029000
12	C	-2.3672065000	-1.4827437000	-0.1830568000
13	H	-2.0658912000	-0.7528666000	-2.1437079000
14	H	-0.6098613000	1.0750143000	-1.6843838000
15	H	-1.8910309000	2.5117755000	1.5668503000
16	H	0.2616639000	1.4321760000	2.3486478000
17	H	1.5181402000	-0.6648990000	2.3289925000
18	H	0.5555471000	-1.2223295000	-1.8149844000
19	O	-5.0893996000	0.3498768000	0.6720165000
20	O	-3.5325845000	-1.2456495000	0.5106026000
21	O	-1.7522231000	-2.4923873000	-0.0001653000
22	H	-3.8657752000	0.7241748000	-1.8098219000
23	H	-2.4194633000	2.5906037000	-1.3142215000
24	H	-3.7152496000	2.5999788000	-0.1049346000
25	O	1.6513053000	-2.2935355000	0.1405919000
26	C	2.4531765000	-2.7502676000	1.2317412000

27	H	2.9284250000	-3.6711915000	0.8793193000
28	H	1.8318152000	-2.9648934000	2.1135664000
29	H	3.2231206000	-2.0043726000	1.4803948000
30	C	1.9572576000	1.8657818000	-0.4757057000
31	C	1.9317962000	0.7841922000	-1.3609734000
32	C	3.0453973000	1.6833996000	0.4426302000
33	C	3.1762882000	0.0060687000	-1.0769288000
34	O	3.7135909000	0.4738443000	0.0871870000
35	O	3.6760922000	-0.8993923000	-1.6879481000
36	O	3.4822003000	2.3289188000	1.3637437000
37	H	1.5834863000	0.8392568000	-2.3928879000
38	H	1.3517149000	2.7662486000	-0.5007990000

Table S59. Energy, thermodynamic quantities, and corresponding xyz coordinates (in Angstroms; calculated with ωB97XD density functional and aug-cc-pVDZ basis set) of 4-methoxystyrene maleic anhydride second transition state for ene mechanism in DMSO (ε = 41.8).

Energy = -1182.564506 hartrees
Total enthalpy = 191.86 kcal/mol
Total entropy = 144.56 cal/mol×K

ATOM		X	Y	Z
1	C	0.1398100000	0.4533600000	-0.7081300000
2	C	-0.5190700000	-0.3259600000	-1.7076600000
3	C	-0.9823200000	-1.5797200000	-1.4383300000
4	C	-0.8419100000	-2.1524800000	-0.1226900000
5	C	-0.1815900000	-1.4724500000	0.8533200000
6	C	0.3213700000	-0.1116700000	0.6369800000
7	C	0.3719300000	1.8235300000	-0.8344000000
8	C	1.3953300000	2.4809500000	0.0735800000
9	C	2.3497800000	1.4578700000	0.6849100000
10	C	3.3641600000	0.8879000000	-0.2635100000
11	C	1.7049900000	0.1991400000	1.2812400000
12	C	2.6904300000	-0.8534400000	0.8156900000
13	H	1.6036600000	0.2361500000	2.3726700000
14	H	0.1995000000	2.2798300000	-1.8122500000
15	H	-0.6447900000	0.0990900000	-2.7048400000
16	H	-1.4634800000	-2.1483700000	-2.2337200000
17	H	-0.0274300000	-1.9442900000	1.8241200000
18	O	4.0876300000	1.5656000000	-0.9754000000
19	O	3.5544100000	-0.4171500000	-0.1002000000
20	O	2.6443100000	-2.0264700000	1.1517000000
21	H	2.9559600000	1.9558400000	1.4581800000
22	H	0.9268600000	3.0404800000	0.8950300000
23	H	1.9825800000	3.2081200000	-0.5050600000
24	O	-1.3076100000	-3.3916800000	0.1871800000
25	C	-2.0937500000	-4.0855500000	-0.7691600000
26	H	-2.3998200000	-5.0186700000	-0.2821100000
27	H	-1.5161300000	-4.3266700000	-1.6761600000
28	H	-2.9923700000	-3.5101700000	-1.0460100000

29	H	-0.4045000000	0.6395400000	1.2431300000
30	C	-1.4192200000	2.5053700000	0.1388000000
31	C	-1.4953600000	1.9437800000	1.4119500000
32	C	-2.4674900000	1.8392300000	-0.6981200000
33	C	-2.5451300000	0.9070800000	1.4008000000
34	O	-3.0358800000	0.8539900000	0.0902300000
35	O	-2.9316500000	0.1847500000	2.3012100000
36	O	-2.7727100000	2.0614200000	-1.8537100000
37	H	-1.1129600000	2.3604300000	2.3417700000
38	H	-1.1249400000	3.5281700000	-0.0877000000

Appendix B: Characterization for Wagner-Jauregg Products

General purification procedure: 2−3 vials containing replicates of the same reaction were combined, the H_2O was removed via pipette, then the organic material was partially dissolved in a minimum amount of EtOAc. The dissolved portion was loaded onto a silica gel column and purified by flash chromatography (gradient eluent from 1:1 EtOAc/hexanes to 100% EtOAc). The column was monitored by TLC to afford purified DA-ene as white solids and, in some cases, DDA products, although when DDA product was a very minor component of the reaction mixture, it was sometimes not isolated. DA-ene product could also be isolated in some cases by rinsing the remaining, insoluble white solid that was not loaded on the column with additional EtOAc. That solid, upon drying under high vacuum, afforded pure DA-ene products8 as white solids. No efforts were made to quantitatively isolate either DDA or ene products.

Additional details for respective products, along with characterization information, are described below:

4b: ^{1}H NMR (400 MHz, acetone-d_6) δ 6.33 (t, J = 6.0 Hz, 2H), 6.29 – 6.19 (m, 3H), 5.13 (dddd, J = 26.2, 6.4, 4.9, 1.1 Hz, 2H), 3.48 (ddd, J = 5.2, 2.2, 1.1 Hz, 1H), 3.38 (ddt, J = 4.9, 2.6, 1.3 Hz, 1H), 3.15 (t, J = 7.1 Hz, 1H), 3.06 (ddd, J = 6.7, 4.2, 1.8 Hz, 1H), 3.01 – 2.87 (m, 2H), 2.78 (dd, J = 12.2, 1.9 Hz, 1H), 2.68 – 2.65 (m, 1H), 2.61 (d, J = 3.7 Hz, 6H), 2.45 (ddd, J = 12.2, 4.2, 1.6 Hz, 1H). ^{13}C{^{1}H} NMR (101 MHz, acetone-d_6) δ 179.66, 178.82, 178.39, 178.03, 141.57, 136.27, 133.76, 131.46, 129.27, 129.27, 129.01, 127.88, 46.49, 44.98, 44.18, 42.82, 41.90, 37.59, 37.05, 30.74, 24.75, 24.58. GC-LRMS (EI+) m/z calculated for $C_{24}H_{22}N_2O_4$ $[M]^+$ 402.2, found 402.1.

8a: ^{1}H NMR (400 MHz, chloroform-d) δ 7.50 – 7.30 (m, 6H), 7.20 – 7.09 (m, 4H), 5.88 (dt, J = 6.7, 2.9 Hz, 1H), 4.86 (dd, J = 7.1, 2.3 Hz, 1H), 3.80 (dt, J = 7.0, 3.1 Hz, 2H), 3.49 (t, J = 8.7 Hz, 1H), 3.29 (ddd, J = 8.5, 5.7, 2.1 Hz, 1H), 3.21 (s, 3H), 3.19 – 3.09 (m, 1H), 2.95 (ddd, J = 15.3, 7.4, 2.2 Hz, 1H), 2.49 (dq, J = 8.8, 2.3 Hz, 1H), 2.28 – 2.08 (m, 1H). ^{13}C{^{1}H} NMR (101 MHz, chloroform-d) δ 177.99, 176.69, 175.61, 174.66, 158.79, 140.92, 132.18, 131.85, 129.34, 129.11, 128.83, 128.65, 126.50, 126.28, 117.59, 92.83, 54.68, 46.26, 45.90, 42.89, 40.70, 40.52, 39.98, 39.71, 23.29. LC-LRMS (ESI+) m/z calculated for $C_{29}H_{25}N_2O_5$ $[M + H]^+$ 481.18, found 481.26.

8b: ^{1}H NMR (400 MHz, chloroform-d) δ 5.70 (dt, J = 6.7, 2.9 Hz, 1H), 4.64 (dd, J = 7.0, 2.4 Hz, 1H), 3.63 (dt, J = 3.9, 2.3 Hz, 1H), 3.57 (dd, J = 7.1, 3.0 Hz, 1H), 3.30 (d, J = 14.6 Hz, 4H), 3.07 (ddd, J = 8.4, 5.9, 1.9 Hz, 1H), 2.96 (dd, J = 7.8, 3.2 Hz, 1H), 2.88 (s, 4H), 2.79 (s, 4H), 2.39 – 2.25 (m, 1H), 2.02 (ddt, J = 12.2, 5.5, 2.5 Hz, 1H). ^{13}C{^{1}H} NMR (101 MHz, chloroform-d) δ 178.94, 177.75, 176.63, 175.69, 158.51, 140.97, 116.96, 92.35, 54.80, 46.36, 45.76, 42.63, 40.67, 40.05, 39.56, 39.26, 24.79, 24.77, 22.97. GC-LRMS (EI+) m/z calculated for $C_{19}H_{20}N_2O_5$ $[M]^+$ 356.1, found 356.1.

8c: Note – due to low solubility in all tested solvents (and failure of recrystallization to afford acceptably pure product) and coelution with **9c** on silica gel under multiple chromatographic methods (preparatory TLC, flash chromatography), this compound is only ~90% pure. ^{1}H NMR (400 MHz, acetone-d_6) δ 9.82 (s, J = 71.4 Hz, 2H), 5.71 (dt, J = 6.5, 2.9 Hz, 1H), 4.78 (dd, J = 7.0, 2.4 Hz, 1H), 3.84 – 3.77 (m, 2H), 3.46 (qd, J = 8.0, 5.3 Hz, 2H), 3.38 – 3.31 (m, 1H), 3.30 (s, 3H), 3.21 (ddd, J = 8.6, 6.0, 2.0 Hz, 1H), 3.11 (dd, J = 7.9, 3.1 Hz, 1H), 2.93 (dd, J = 8.0, 3.1 Hz, 1H), 2.67 – 2.57 (m, 1H), 2.54 – 2.46 (m, 1H). ^{13}C{^{1}H} NMR (101 MHz, acetone-d_6) δ 176.96, 152.57, 143.07, 116.47, 93.10, 54.60, 48.46, 47.85, 44.56, 42.65,

40.83, 40.06, 39.95, 23.21. LC-LRMS (ESI+) m/z calculated for $C_{17}H_{17}N_2O_5$ $[M + H]^+$ 329.11, found 329.19.

9a: ^{1}H NMR (400 MHz, chloroform-*d*) δ 7.56 – 7.36 (m, 7H), 7.32 – 7.18 (m, 4H), 7.04 (d, *J* = 8.6 Hz, 1H), 6.88 – 6.77 (m, 1H), 4.22 – 4.11 (m, 1H), 3.84 (s, 3H), 3.77 (q, *J* = 4.3 Hz, 1H), 3.41 (ddd, *J* = 11.3, 8.8, 5.8 Hz, 1H), 3.31 (ddd, *J* = 9.1, 5.5, 3.3 Hz, 1H), 2.89 (dd, *J* = 18.0, 9.4 Hz, 1H), 2.59 – 2.38 (m, 2H), 2.24 – 2.08 (m, 1H). ^{13}C{^{1}H} NMR (101 MHz, chloroform-*d*) δ 177.80, 177.69, 175.15, 174.90, 159.21, 131.77, 131.75, 130.75, 129.41, 129.35, 129.27, 128.99, 128.95, 128.90, 126.62, 126.50, 115.23, 114.89, 55.49, 45.01, 42.59, 37.97, 36.56, 31.95, 30.23. LC-LRMS (ESI+) m/z calculated for $C_{29}H_{25}N_2O_5$ $[M + H]^+$ 481.18, found 481.26.

9b: ^{1}H NMR (400 MHz, acetone-d_6) δ 7.44 (d, *J* = 2.7 Hz, 1H), 6.98 (d, *J* = 8.5 Hz, 1H), 6.80 (td, *J* = 7.6, 2.7 Hz, 1H), 4.20 (d, *J* = 8.8 Hz, 1H), 3.80 (s, 3H), 3.54 – 3.19 (m, 3H), 2.87 (s, 6H), 2.82 – 2.67 (m, 1H), 2.42 (dt, *J* = 13.3, 5.7 Hz, 1H), 2.29 (dd, *J* = 17.9, 5.2 Hz, 1H), 1.99 – 1.79 (m, 1H). ^{13}C{^{1}H} NMR (101 MHz, acetone-d_6) δ 179.55, 179.50, 177.22, 176.57, 159.36, 132.82, 129.33, 129.31, 116.09, 113.57, 55.43, 44.51, 42.88, 38.34, 37.48, 32.66, 30.22, 25.06, 24.72. GC-LRMS (EI+) m/z calculated for $C_{19}H_{20}N_2O_5$ $[M]^+$ 356.1, found 356.1.

9c: Note – due to low solubility in all tested solvents (and failure of recrystallization to afford acceptably pure product), coelution with **8c** on silica gel under multiple chromatographic conditions (flash chromatography), and decomposition on preparatory TLC, this compound was not successfully isolated. A spectrum containing a reaction mixture with **9c** as the major component, is included in the SI.

11a: ^{1}H NMR (400 MHz, acetone-d_6) δ 7.48 – 7.24 (m, 5H), 5.71 (dt, *J* = 6.7, 2.9 Hz, 1H), 4.77 (dd, *J* = 7.1, 2.4 Hz, 1H), 4.63 – 4.39 (m, 2H), 3.59 (q, *J* = 2.5 Hz, 1H), 3.47 (dd, *J* = 7.0, 3.1 Hz, 1H), 3.40 (t, *J* = 8.8 Hz, 1H), 3.26 – 3.05 (m, 2H), 2.98 – 2.90 (m, 1H), 2.90 – 2.81 (m, 6H), 2.69 (ddd, *J* = 15.2, 7.3, 2.1 Hz, 1H), 2.58 (dq, *J* = 8.9, 2.2 Hz, 1H), 2.14 – 2.07 (m, 1H). ^{13}C{^{1}H} NMR (101 MHz, acetone-d_6) δ 179.58, 178.15, 177.43, 176.63, 158.82, 142.86, 138.40, 129.36, 129.07, 128.63, 128.54, 128.42, 116.86, 94.30, 70.06, 47.16, 46.55, 43.43, 41.61, 41.00, 40.44, 40.03, 33.31, 24.66, 23.37, 14.57. GC-LRMS (EI+) m/z calculated for $C_{25}H_{24}N_2O_5$ $[M]^+$ 432.2, found 432.2.

11c: ^{1}H NMR (400 MHz, chloroform-*d*) δ 5.73 (dt, *J* = 6.8, 2.9 Hz, 1H), 5.67 (dt, *J* = 6.3, 1.7 Hz, 1H), 3.61 (dq, *J* = 2.8, 1.4 Hz, 1H), 3.53 (dd, *J* = 6.4, 3.0 Hz, 1H), 3.25 (t, *J* = 8.9 Hz, 1H), 3.12 – 2.92 (m, 3H), 2.89 (s, 3H), 2.86 – 2.75 (m, 4H), 2.36 (td, *J* = 9.8, 4.9 Hz, 1H), 2.02 (dp, *J* = 15.4, 2.6 Hz, 1H), 1.72 (d, *J* = 1.7 Hz, 3H). ^{13}C{^{1}H} NMR (101 MHz, chloroform-*d*) δ 178.92, 177.92, 177.26, 177.10, 140.87, 140.46, 123.64, 117.53, 46.63, 45.10, 42.56, 40.88, 40.86, 40.82, 40.29, 24.84, 24.74, 22.64, 22.06. GC-LRMS (EI+) m/z calculated for $C_{19}H_{20}N_2O_4$ $[M]^+$ 340.1, found 340.1.

11h: Note – compound decomposes on silica gel, as determined by 2D TLC experiments. Complete purification of product was not possible. Date: ^{1}H NMR (400 MHz, acetone-d_6) δ 6.27 (dd, *J* = 6.9, 2.2 Hz, 1H), 5.81 (dt, *J* = 7.8, 3.0 Hz, 1H), 4.33 (dd, *J* = 8.7, 5.6 Hz, 1H), 3.85 (ddt, *J* = 4.0, 3.1, 1.5 Hz, 1H), 3.62 (dd, *J* = 6.9, 3.0 Hz, 1H), 3.42 (t, *J* = 9.0 Hz, 1H), 3.29 (dd, *J* = 7.7, 3.2 Hz, 1H), 3.21 (ddd, *J* = 8.4, 5.9, 2.0 Hz, 1H), 3.01 (dd, *J* = 7.8, 3.1 Hz, 1H), 2.81 (s, 3H), 2.74 (d, *J* = 3.0 Hz, 3H), 2.70 (ddd, *J* = 15.5, 7.3, 2.1 Hz, 1H), 2.43 (s, 1H). ^{13}C{^{1}H} NMR (101 MHz, acetone-d_6) δ 179.35, 177.57, 177.26, 177.16, 176.49, 175.54, 139.61, 132.30, 121.49, 119.26, 63.47, 61.19, 47.73, 46.14, 45.39, 44.29, 42.73, 41.51, 41.07, 33.21,

30.42, 24.76, 24.67, 24.52, 22.95. GC-LRMS (EI+) m/z calculated for $C_{18}H_{17}BrN_2O_4$ $[M]^+$ 404.0, found 404.0.

11i: ^{1}H NMR (400 MHz, acetone-*d*$_6$) δ 6.80 (dd, *J* = 6.6, 1.8 Hz, 1H), 5.84 (dt, *J* = 6.7, 3.0 Hz, 1H), 4.16 (h, *J* = 1.4 Hz, 1H), 3.72 (dd, *J* = 6.6, 3.2 Hz, 1H), 3.67 (s, 3H), 3.37 (t, *J* = 8.7 Hz, 1H), 3.18 (ddd, *J* = 11.0, 6.9, 2.5 Hz, 2H), 3.09 (dd, *J* = 8.1, 3.2 Hz, 1H), 3.01 – 2.79 (m, 1H), 2.72 (s, 3H), 2.65 (s, 4H), 2.18 – 2.09 (m, 1H). ^{13}C{^{1}H} NMR (101 MHz, acetone-*d*$_6$) δ 179.48, 178.01, 177.47, 176.60, 165.09, 140.35, 137.58, 136.84, 119.71, 51.70, 46.80, 45.36, 43.23, 41.59, 41.13, 40.71, 36.97, 30.31, 24.61, 23.96. GC-LRMS (EI+) m/z calculated for $C_{20}H_{20}N_2O_6$ $[M]^+$ 384.1, found 384.1.

12a: ^{1}H NMR (400 MHz, acetone-*d*$_6$) δ 7.66 – 7.21 (m, 6H), 7.01 (d, *J* = 8.5 Hz, 1H), 6.88 (dd, *J* = 8.5, 2.7 Hz, 1H), 5.13 (s, 2H), 4.20 (d, *J* = 8.8 Hz, 1H), 3.58 – 3.22 (m, 3H), 2.90 (d, *J* = 20.4 Hz, 6H), 2.78 (dd, *J* = 17.9, 9.0 Hz, 1H), 2.44 (dt, *J* = 13.4, 5.8 Hz, 1H), 2.31 (dd, *J* = 17.9, 5.2 Hz, 1H), 1.92 (ddd, *J* = 13.9, 10.5, 4.1 Hz, 1H). ^{13}C{^{1}H} NMR (101 MHz, acetone-*d*$_6$) δ 179.66, 179.61, 177.65, 176.67, 158.76, 138.40, 133.10, 129.91, 129.53, 129.38, 128.76, 128.64, 117.27, 114.53, 70.59, 44.63, 43.06, 38.53, 37.75, 32.90, 30.18, 25.23, 24.88. GC-LRMS (EI+) m/z calculated for $C_{25}H_{24}N_2O_5$ $[M]^+$ 432.2, found 432.2.

12b: ^{1}H NMR (400 MHz, chloroform-*d*) δ 7.85 (dd, *J* = 2.2, 0.9 Hz, 1H), 7.21 (dd, *J* = 8.2, 2.1 Hz, 1H), 6.84 (d, *J* = 8.2 Hz, 1H), 4.00 (d, *J* = 8.8 Hz, 1H), 3.45 (dt, *J* = 7.1, 4.1 Hz, 1H), 3.28 (td, *J* = 9.1, 6.0 Hz, 1H), 3.14 (ddd, *J* = 9.4, 5.6, 3.9 Hz, 1H), 3.02 (d, *J* = 1.8 Hz, 6H), 2.72 (dd, *J* = 18.0, 9.2 Hz, 1H), 2.37 (dd, *J* = 18.0, 5.6 Hz, 1H), 2.26 (dt, *J* = 12.9, 6.3 Hz, 1H), 2.05 (ddd, *J* = 12.6, 9.3, 4.2 Hz, 1H), 1.33 (s, 9H). ^{13}C{^{1}H} NMR (101 MHz, chloroform-*d*) δ 179.02, 178.60, 176.65, 176.13, 151.00, 132.14, 129.30, 127.86, 126.87, 125.09, 44.30, 42.71, 38.35, 36.24, 34.75, 31.87, 31.36, 29.48, 25.39, 25.21. GC-LRMS (EI+) m/z calculated for $C_{22}H_{26}N_2O_4$ $[M]^+$ 382.2, found 382.2.

12c: ^{1}H NMR (400 MHz, acetone-*d*$_6$) δ 7.65 (s, 1H), 7.09 – 6.98 (m, 1H), 6.94 (d, *J* = 7.8 Hz, 1H), 4.18 (d, *J* = 8.8 Hz, 1H), 3.44 (ddd, *J* = 10.1, 8.8, 6.2 Hz, 1H), 3.40 – 3.27 (m, 2H), 2.91 (s, 3H), 2.87 (s, 3H), 2.83 – 2.80 (m, 2H), 2.42 – 2.28 (m, 4H), 1.99 – 1.85 (m, 1H). ^{13}C{^{1}H} NMR (101 MHz, acetone-*d*$_6$) δ 179.72, 179.55, 177.41, 176.65, 137.38, 131.73, 128.67, 128.27, 44.46, 42.98, 38.69, 37.88, 32.84, 30.48, 30.35, 30.29, 30.15, 30.10, 29.91, 29.77, 29.71, 29.52, 29.33, 25.18, 24.86, 21.21. GC-LRMS (EI+) m/z calculated for $C_{19}H_{20}N_2O_4$ $[M]^+$ 340.1, found 340.1.

12d: ^{1}H NMR (400 MHz, acetone-*d*$_6$) δ 7.84 (dt, *J* = 7.8, 1.3 Hz, 1H), 7.29 (td, *J* = 7.5, 1.4 Hz, 1H), 7.21 (ddd, *J* = 8.6, 7.4, 1.3 Hz, 1H), 7.08 (dd, *J* = 7.7, 1.4 Hz, 1H), 4.23 (d, *J* = 8.9 Hz, 1H), 3.53 – 3.30 (m, 3H), 2.90 (d, *J* = 16.7 Hz, 6H), 2.79 (dd, *J* = 17.9, 8.8 Hz, 1H), 2.51 – 2.35 (m, 1H), 2.30 (dd, *J* = 17.9, 5.0 Hz, 1H), 2.00 – 1.85 (m, 1H). ^{13}C{^{1}H} NMR (101 MHz, acetone-*d*$_6$) δ 179.60, 179.39, 177.30, 176.51, 137.57, 135.17, 131.75, 131.15, 128.33, 127.82, 44.21, 42.87, 38.52, 38.20, 32.80, 30.23, 25.09, 24.76. GC-LRMS (EI+) m/z calculated for $C_{18}H_{18}N_2O_4$ $[M]^+$ 326.1, found 326.1.

12e: ^{1}H NMR (400 MHz, acetone-*d*$_6$) δ 8.16 (t, *J* = 1.5 Hz, 1H), 7.81 – 7.63 (m, 2H), 7.58 – 7.44 (m, 3H), 7.44 – 7.32 (m, 1H), 7.19 (d, *J* = 8.0 Hz, 1H), 4.33 (d, *J* = 8.8 Hz, 1H), 3.62 – 3.35 (m, 3H), 2.92 (d, *J* = 16.4 Hz, 6H), 2.53 – 2.33 (m, 2H), 2.03 – 1.95 (m, 1H). ^{13}C{^{1}H} NMR (101 MHz, DMSO-*d*$_6$) δ 179.16, 178.76, 176.85, 176.27, 139.72, 138.64, 136.08, 131.27, 129.04, 128.19, 127.87, 127.55, 126.54, 125.14, 42.57, 41.85, 37.30, 36.45, 32.15, 27.88, 24.85, 24.51. GC-LRMS (EI+) m/z calculated for $C_{24}H_{22}N_2O_4$ $[M]^+$ 402.2, found 402.2.

12f: ^{1}H NMR (400 MHz, DMSO-*d*$_6$) δ 7.55 (dd, *J* = 10.3, 2.3 Hz, 1H), 7.12 – 7.05 (m, 2H), 4.25 (d, *J* = 8.8 Hz, 1H), 3.45 – 3.36 (m, 1H), 3.30 – 3.21 (m, 1H), 2.97 – 2.77 (m, 7H), 2.75 – 2.61 (m, 1H), 2.41 – 2.28 (m, 1H), 2.22 (dd, *J* = 17.8, 4.4 Hz, 1H), 1.78 (ddd, *J* = 13.5, 10.7, 3.2 Hz, 1H). ^{13}C{^{1}H} NMR (101 MHz, DMSO-*d*$_6$) δ 178.67, 178.48, 176.20, 175.94, 161.68, 159.27, 134.40, 132.80, 129.15, 115.89, 113.58, 42.41, 41.28, 39.94, 39.73, 39.52, 39.31, 39.10, 38.89, 38.69, 36.55, 36.15, 31.90, 27.62, 24.64, 24.25. GC-LRMS (EI+) m/z calculated for $C_{18}H_{17}FN_2O_4$ [M]$^+$ 344.1, found 344.1.

12g: ^{1}H NMR (400 MHz, DMSO-*d*$_6$) δ 7.79 (dd, *J* = 2.4, 1.0 Hz, 1H), 7.29 (ddd, *J* = 8.3, 2.4, 0.8 Hz, 1H), 7.09 (d, *J* = 8.3 Hz, 1H), 4.25 (d, *J* = 8.8 Hz, 1H), 3.41 – 3.34 (m, 1H), 3.28 (dt, *J* = 15.2, 5.8 Hz, 2H), 2.84 (d, *J* = 13.2 Hz, 6H), 2.70 (dd, *J* = 17.9, 8.5 Hz, 1H), 2.36 – 2.27 (m, 1H), 2.22 (dd, *J* = 17.9, 4.7 Hz, 1H), 1.80 (ddd, *J* = 13.5, 10.6, 3.7 Hz, 1H). ^{13}C{^{1}H} NMR (101 MHz, DMSO-*d*$_6$) δ 178.87, 178.62, 176.43, 176.16, 135.97, 132.97, 131.30, 129.33, 129.32, 126.77, 42.40, 41.31, 36.83, 36.46, 32.15, 27.53, 24.90, 24.49. GC-LRMS (EI+) m/z calculated for $C_{18}H_{17}ClN_2O_4$ [M]$^+$ 360.1, found 360.1.

12h: ^{1}H NMR (400 MHz, acetone-*d*$_6$) δ 8.05 (dd, *J* = 2.2, 1.0 Hz, 1H), 7.40 (ddd, *J* = 8.2, 2.2, 0.8 Hz, 1H), 7.09 (d, *J* = 8.3 Hz, 1H), 4.27 (d, *J* = 8.9 Hz, 1H), 3.49 (ddd, *J* = 10.7, 8.9, 6.3 Hz, 1H), 3.44 – 3.32 (m, 2H), 2.92 (s, 2H), 2.87 (s, 2H), 2.84 – 2.75 (m, 1H), 2.49 (ddd, *J* = 13.5, 6.2, 4.9 Hz, 1H), 2.33 (dd, *J* = 17.9, 4.7 Hz, 1H), 2.00 – 1.84 (m, 1H). ^{13}C{^{1}H} NMR (101 MHz, acetone-*d*$_6$) δ 179.32, 179.20, 177.00 176.42, 137.26, 134.40, 133.63, 130.76, 130.57, 121.14, 43.88, 42.46, 38.20, 38.11, 32.96, 28.89, 25.23, 24.82. GC-LRMS (EI+) m/z calculated for $C_{18}H_{17}BrN_2O_4$ [M]$^+$ 404.0, found 404.0.

12i: ^{1}H NMR (400 MHz, DMSO-*d*$_6$) δ 8.37 (s, 1H), 7.80 (dd, *J* = 8.0, 1.9 Hz, 1H), 7.21 (d, *J* = 8.1 Hz, 1H), 4.33 (d, *J* = 8.8 Hz, 1H), 3.86 (s, 3H), 3.40 (dd, *J* = 9.5, 6.9 Hz, 2H), 2.84 (d, *J* = 13.6 Hz, 7H), 2.71 (dd, *J* = 18.0, 8.4 Hz, 1H), 2.31 (dt, *J* = 13.2, 5.7 Hz, 1H), 2.22 (dd, *J* = 17.7, 4.4 Hz, 1H), 1.95 – 1.76 (m, 1H). ^{13}C{^{1}H} NMR (101 MHz, DMSO-d$_6$) δ 178.92, 178.55, 176.51, 176.11, 166.02, 142.48, 131.39, 130.71, 128.19, 127.97, 127.50, 72.06, 52.21, 42.25, 41.50, 37.03, 32.19, 27.33, 24.89, 24.50. GC-LRMS (EI+) m/z calculated for $C_{20}H_{20}N_2O_6$ [M]$^+$ 384.1, found 384.1.

12j: ^{1}H NMR (400 MHz, acetone-*d*$_6$) δ 8.22 (s, 1H), 7.57 (dd, *J* = 8.1, 2.0 Hz, 1H), 7.40 (d, *J* = 8.1 Hz, 1H), 4.37 (d, *J* = 8.9 Hz, 1H), 3.61 – 3.40 (m, 3H), 2.93 (s, 3H), 2.88 (s, 3H), 2.77 (dd, *J* = 15.8, 7.8 Hz, 1H), 2.61 – 2.47 (m, 1H), 2.37 (dd, *J* = 17.9, 5.1 Hz, 1H), 2.03 – 1.92 (m, 1H). ^{13}C{^{1}H} NMR (101 MHz, acetone-*d*$_6$) δ 206.26, 179.32, 179.10, 177.03, 176.37, 133.32, 130.26, 129.90, 129.59, 127.73, 124.46, 43.68, 42.62, 38.55, 38.30, 33.08, 28.56, 25.30, 24.88. GC-LRMS (EI+) m/z calculated for $C_{19}H_{17}F_3N_2O_4$ [M]$^+$ 394.1, found 394.1.

Appendix C: ^{1}H and ^{13}C NMR Spectra of Purified Wagner-Jauregg Products

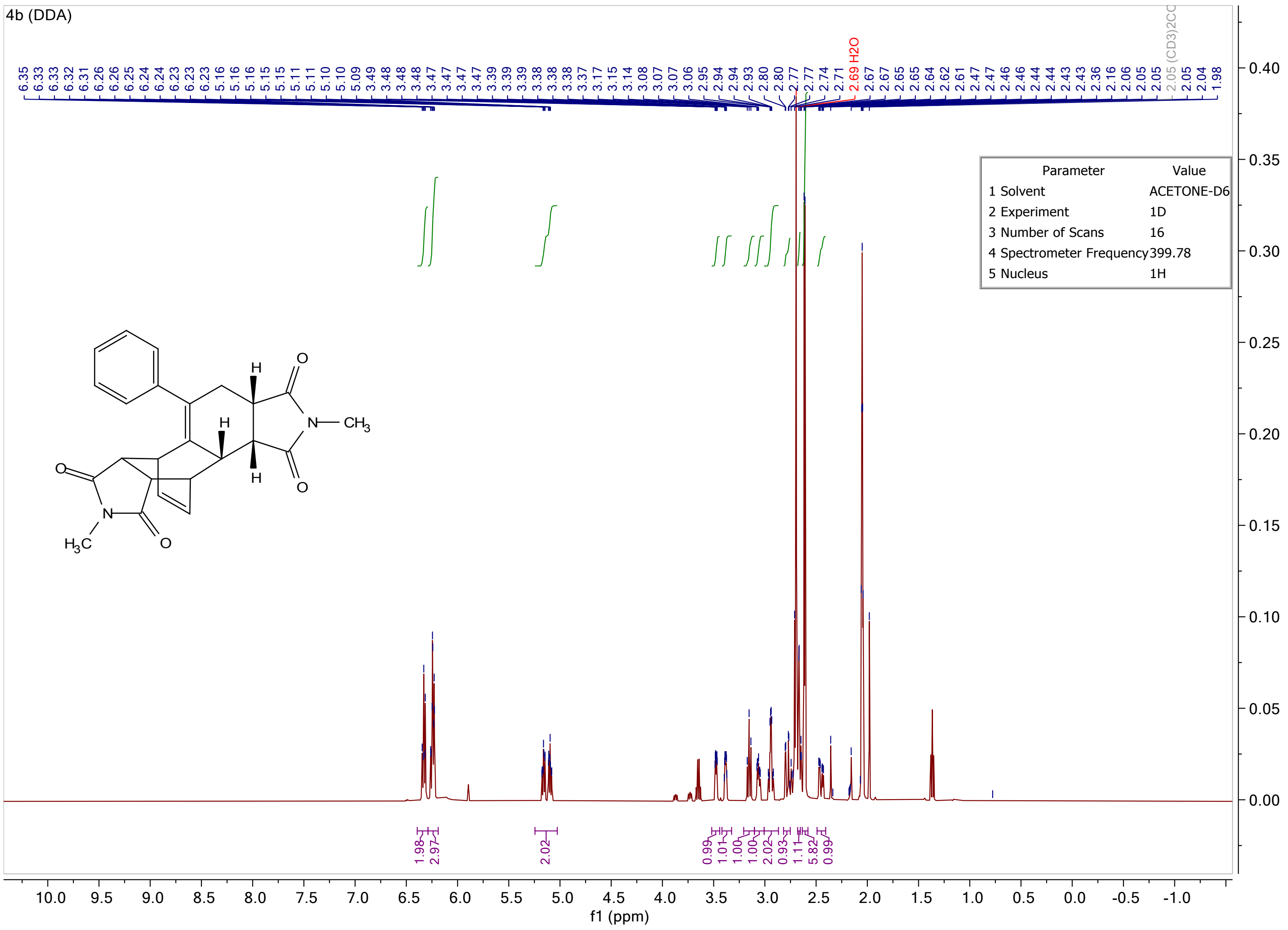

Figure S4. ^{1}H NMR spectrum of DDA adduct **4b**.

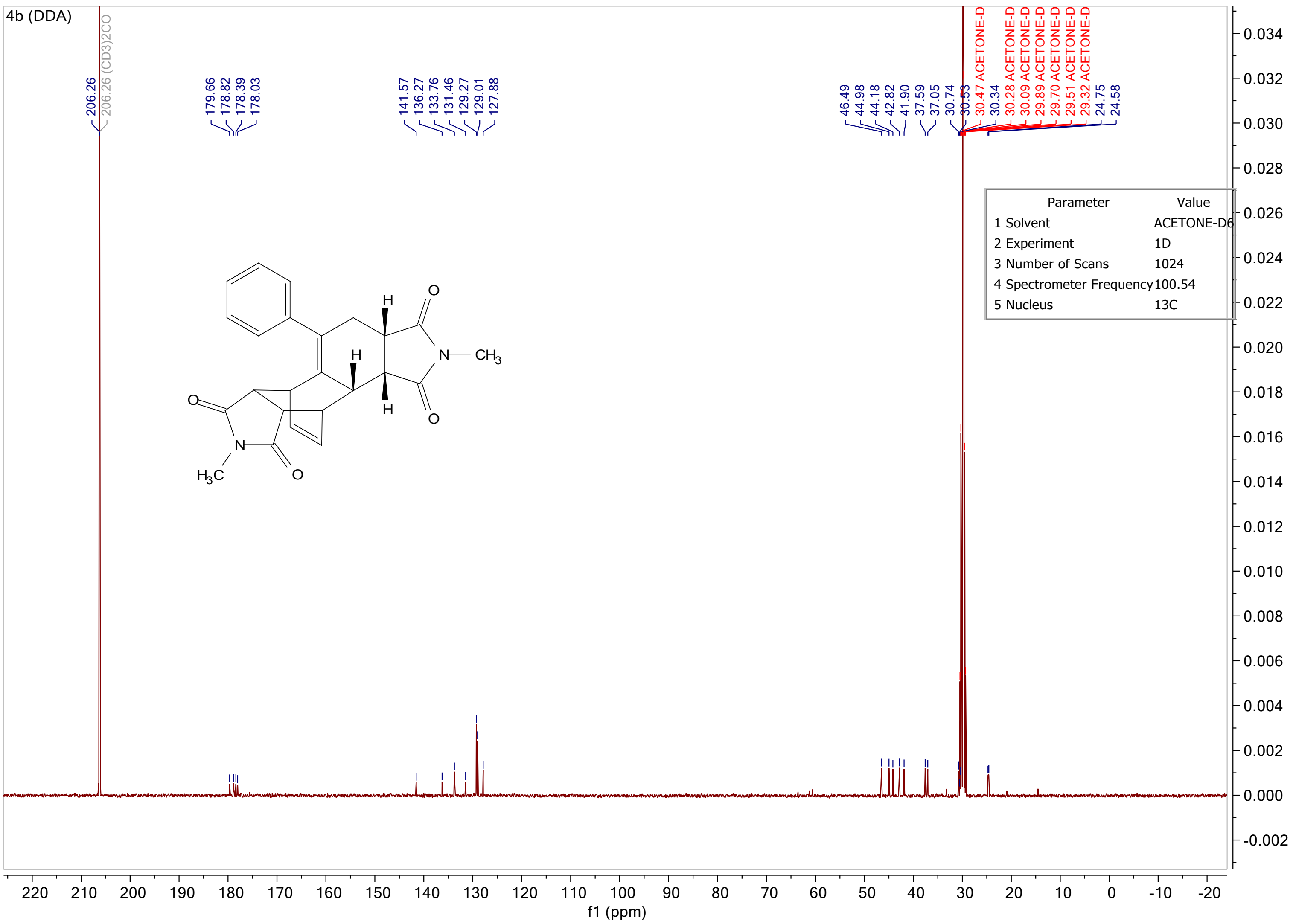

Figure S5. ^{13}C NMR spectrum of DDA adduct **4b**.

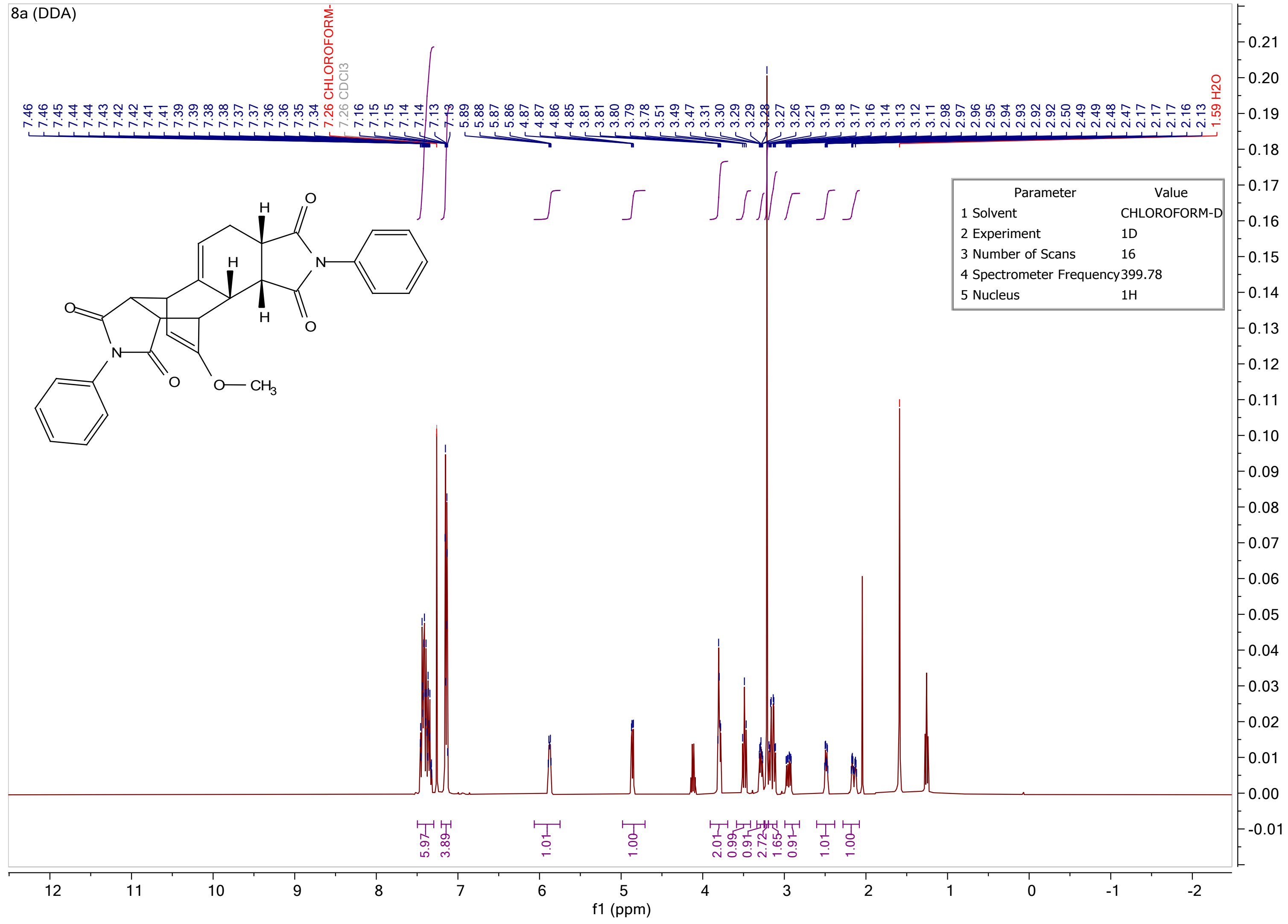

Figure S6. ^{1}H NMR spectrum of DDA adduct **8a**.

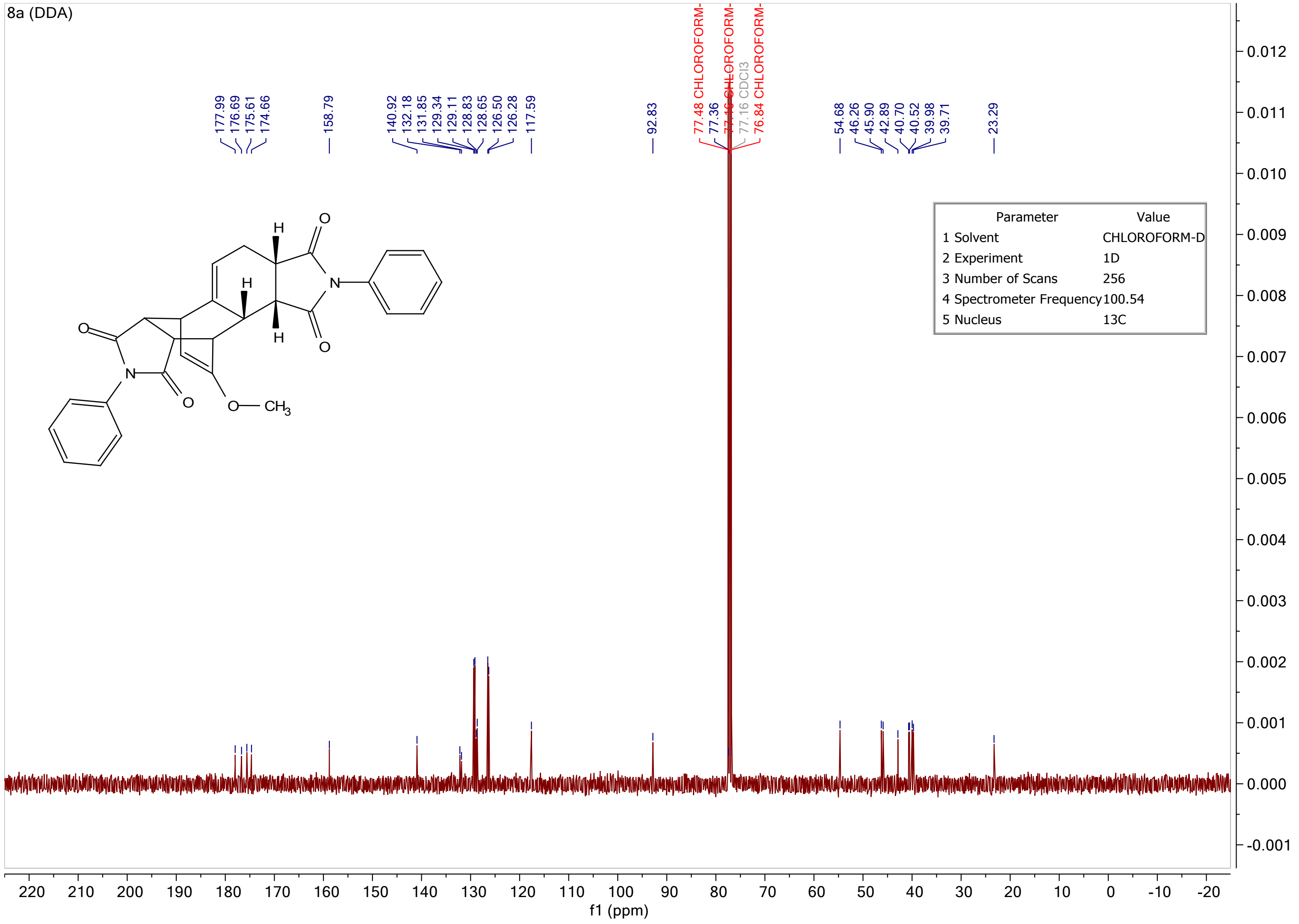

Figure S7. ^{13}C NMR spectrum of DDA adduct **8a**.

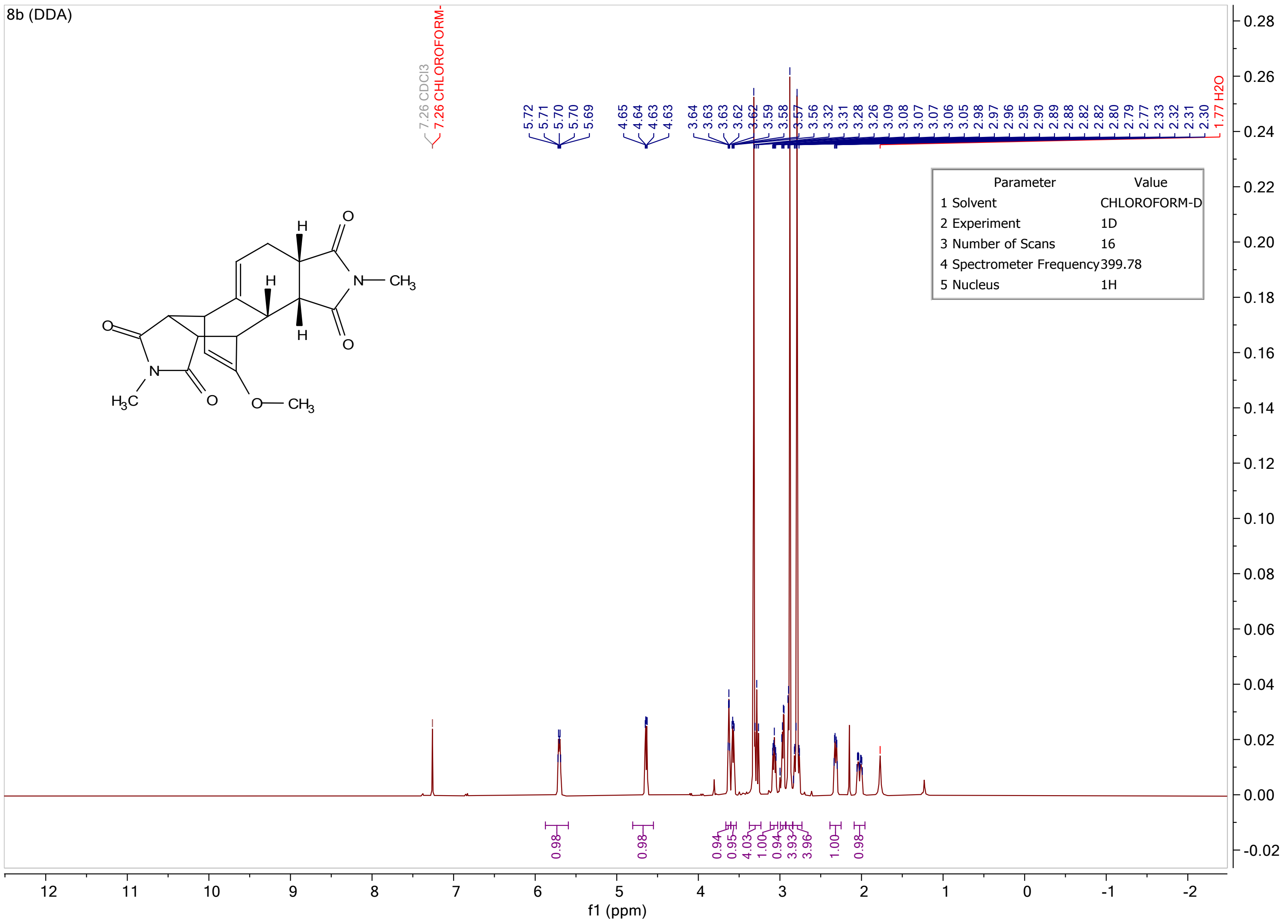

Figure S8. ^{1}H NMR spectrum of DDA adduct **8b**.

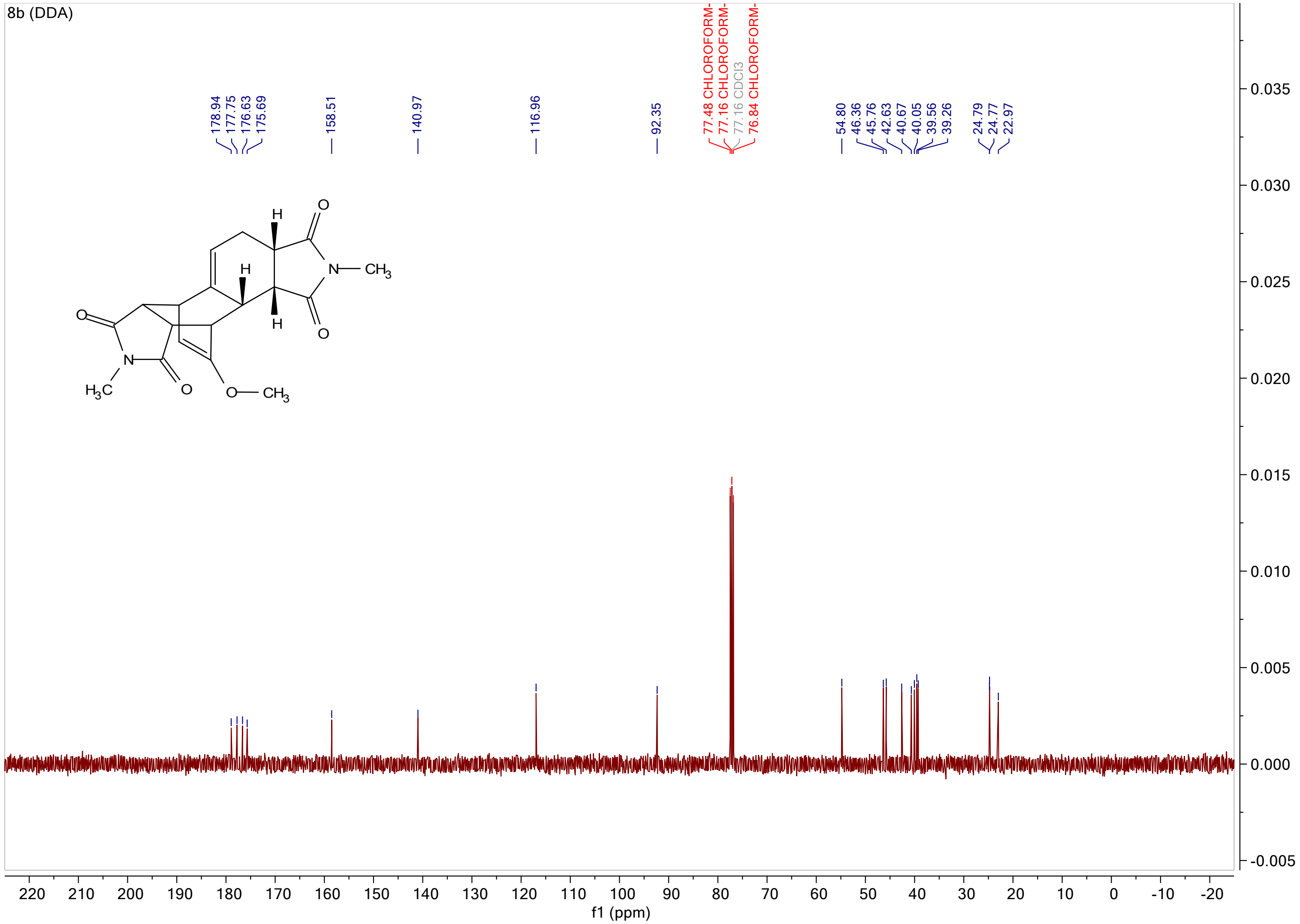

Figure S9. ^{13}C NMR spectrum of DDA adduct **8b**.

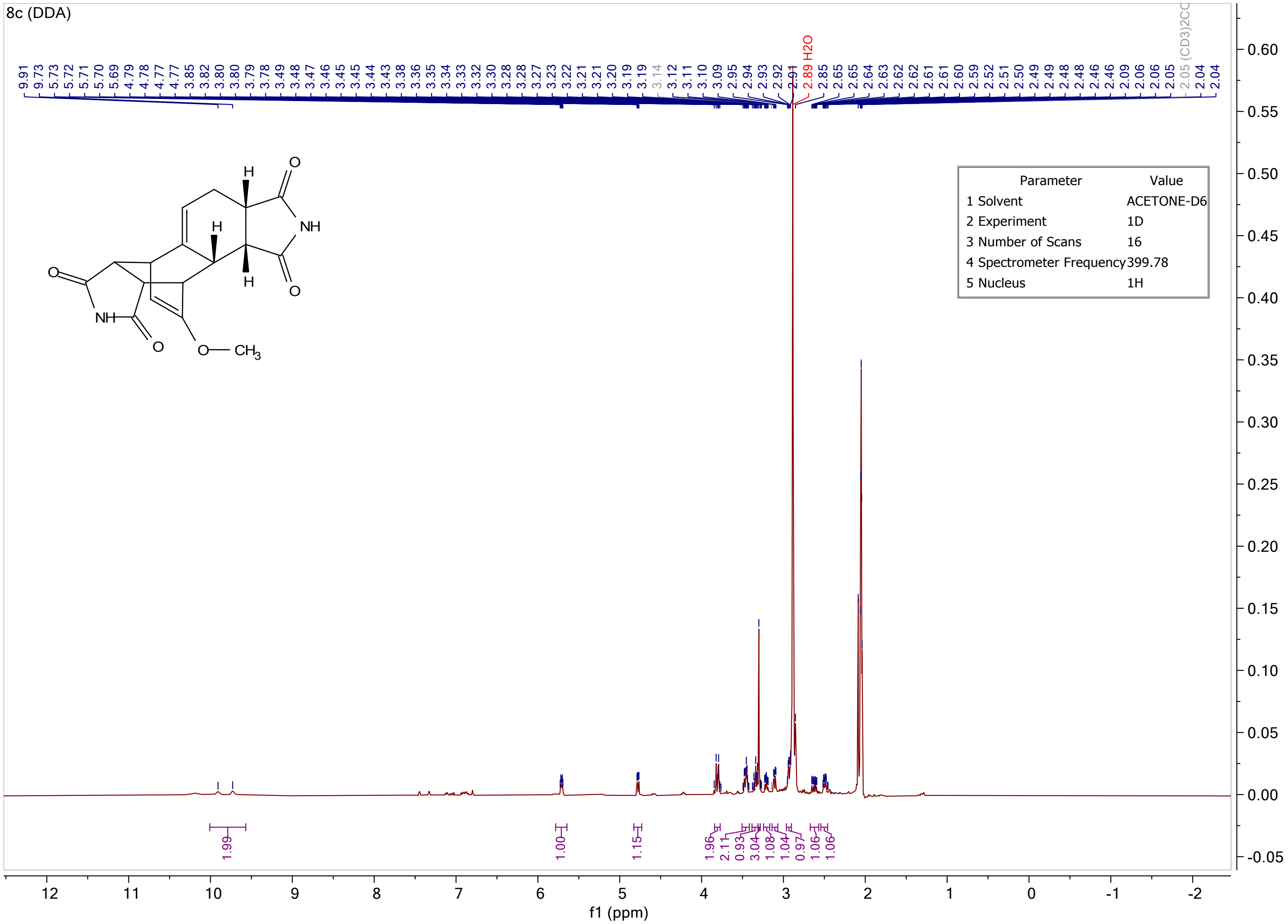

Figure S10. ^{1}H NMR spectrum of DDA adduct **8c**.

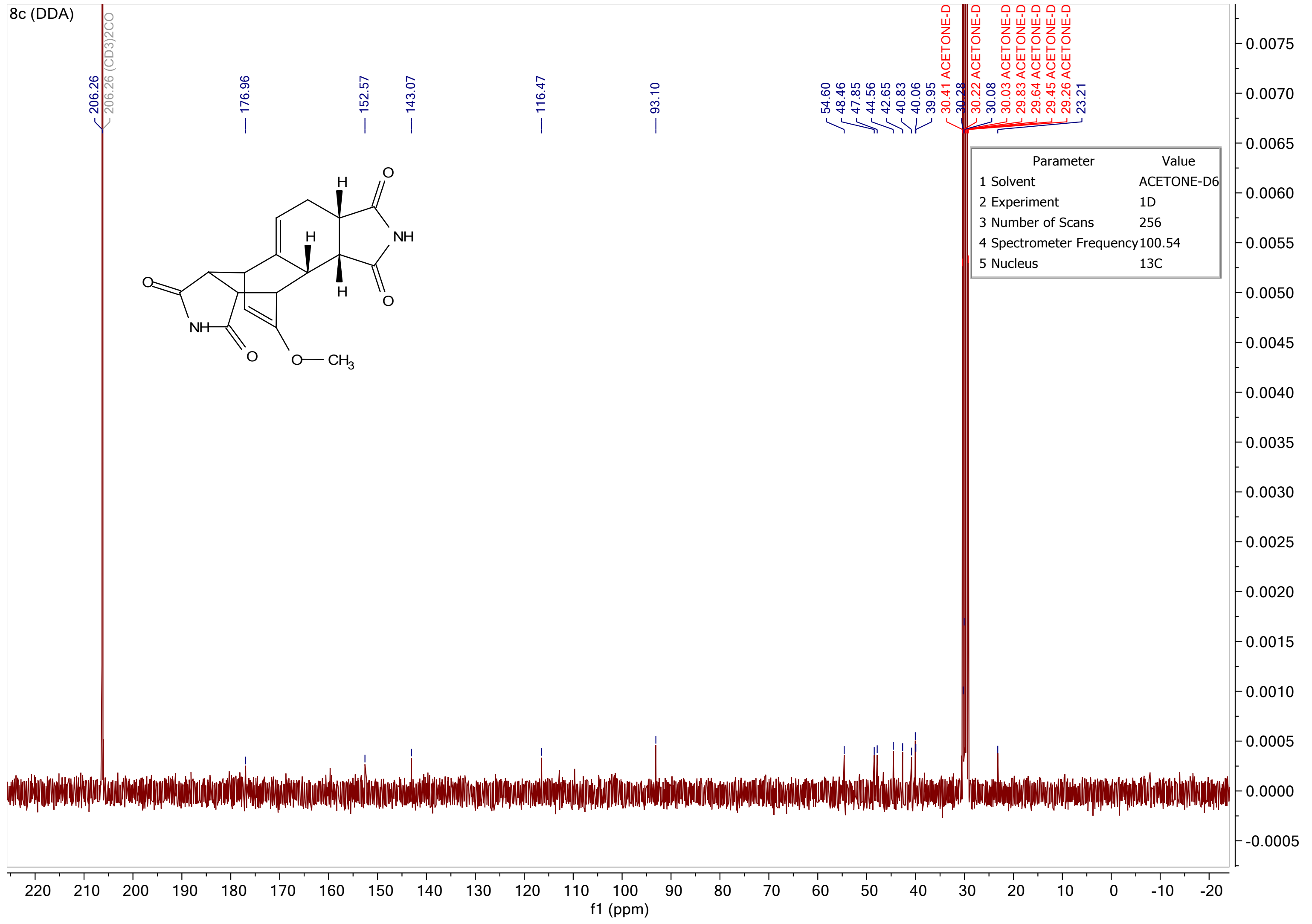

Figure S11. ^{13}C NMR spectrum of DDA adduct **8c**.

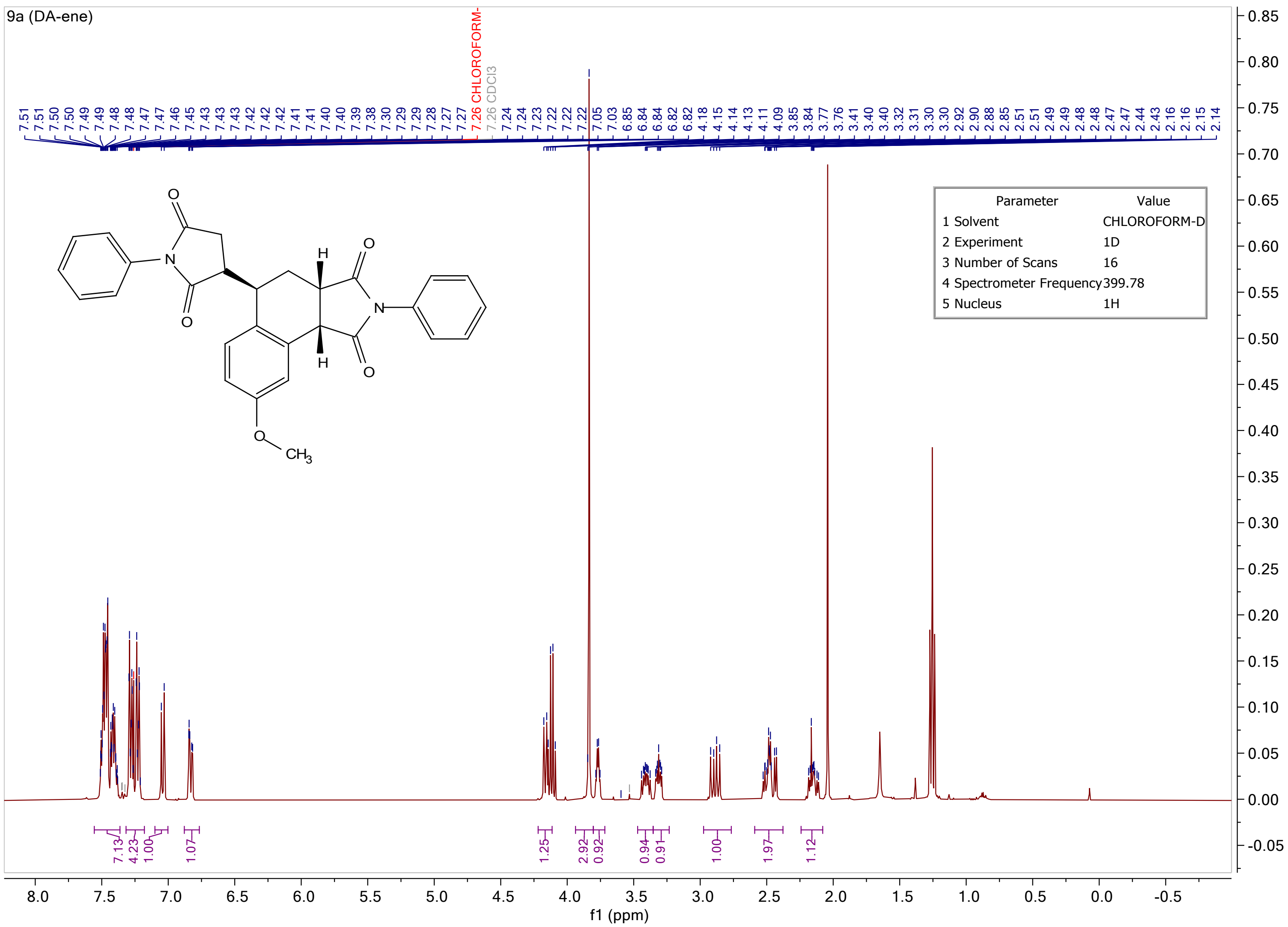

Figure S12. ^1H NMR spectrum of DA-ene adduct **9a**.

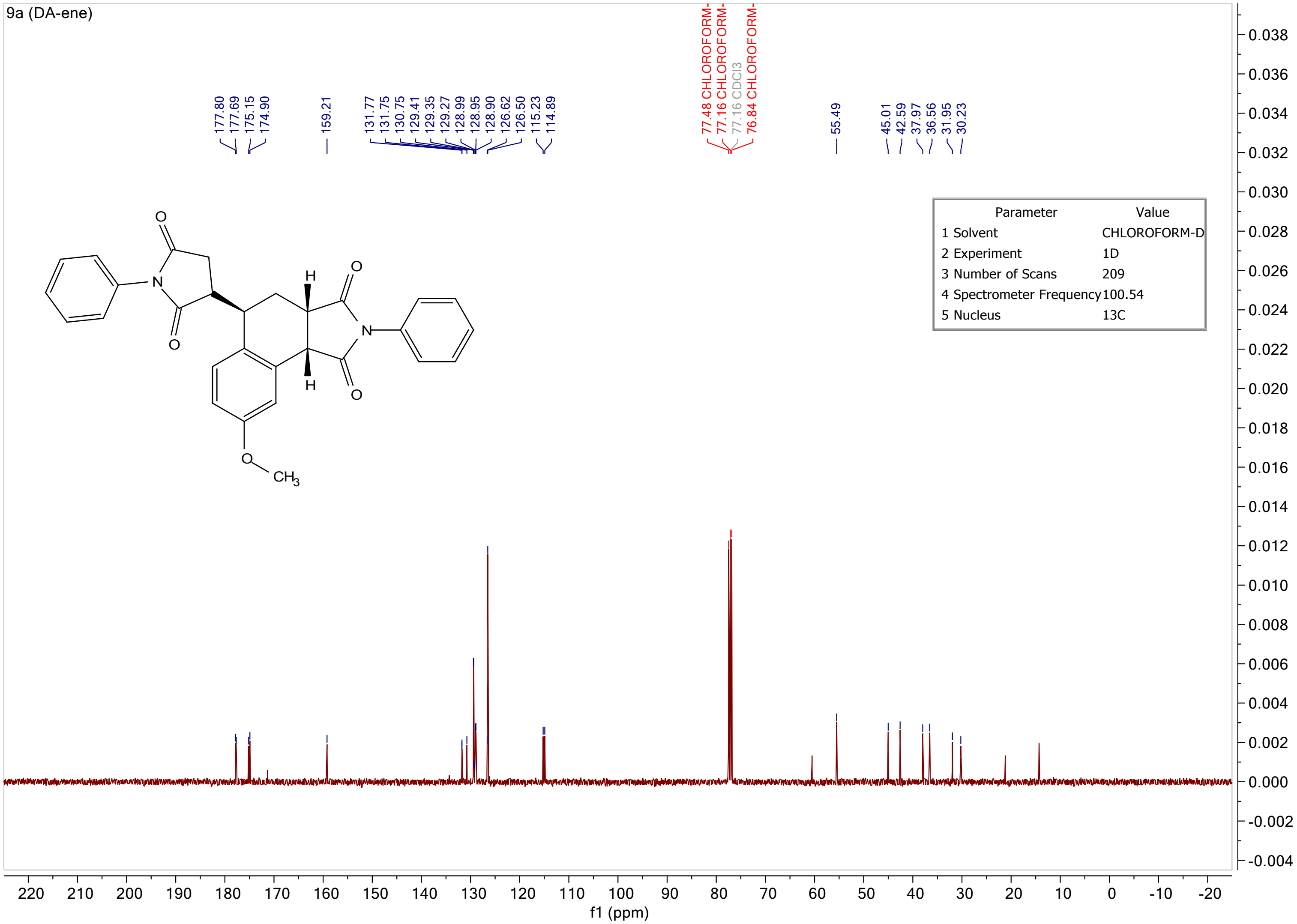

Figure S13. ^{13}C NMR spectrum of DA-ene adduct **9a**.

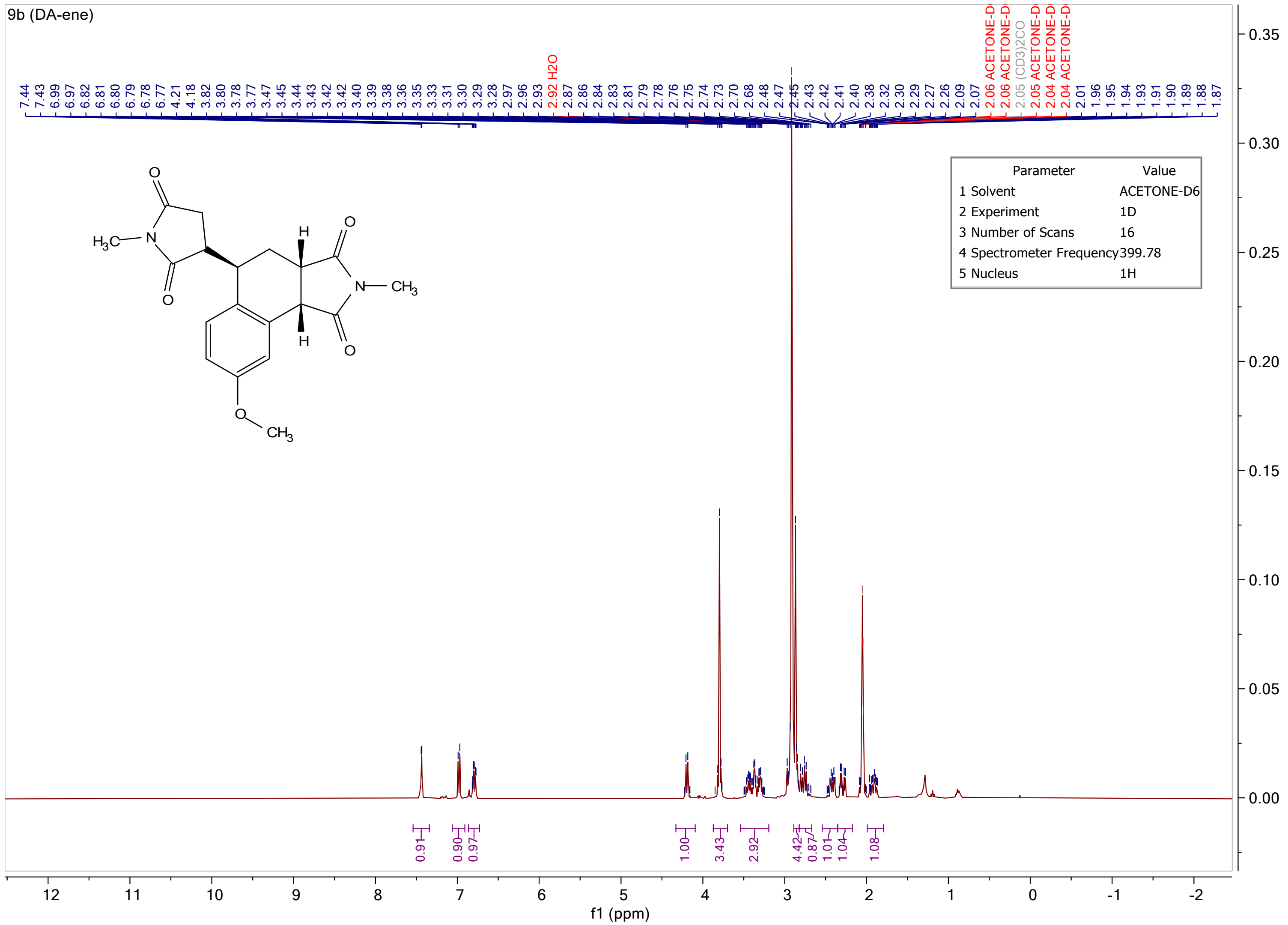

Figure S14. ^{1}H NMR spectrum of DA-ene adduct **9b**.

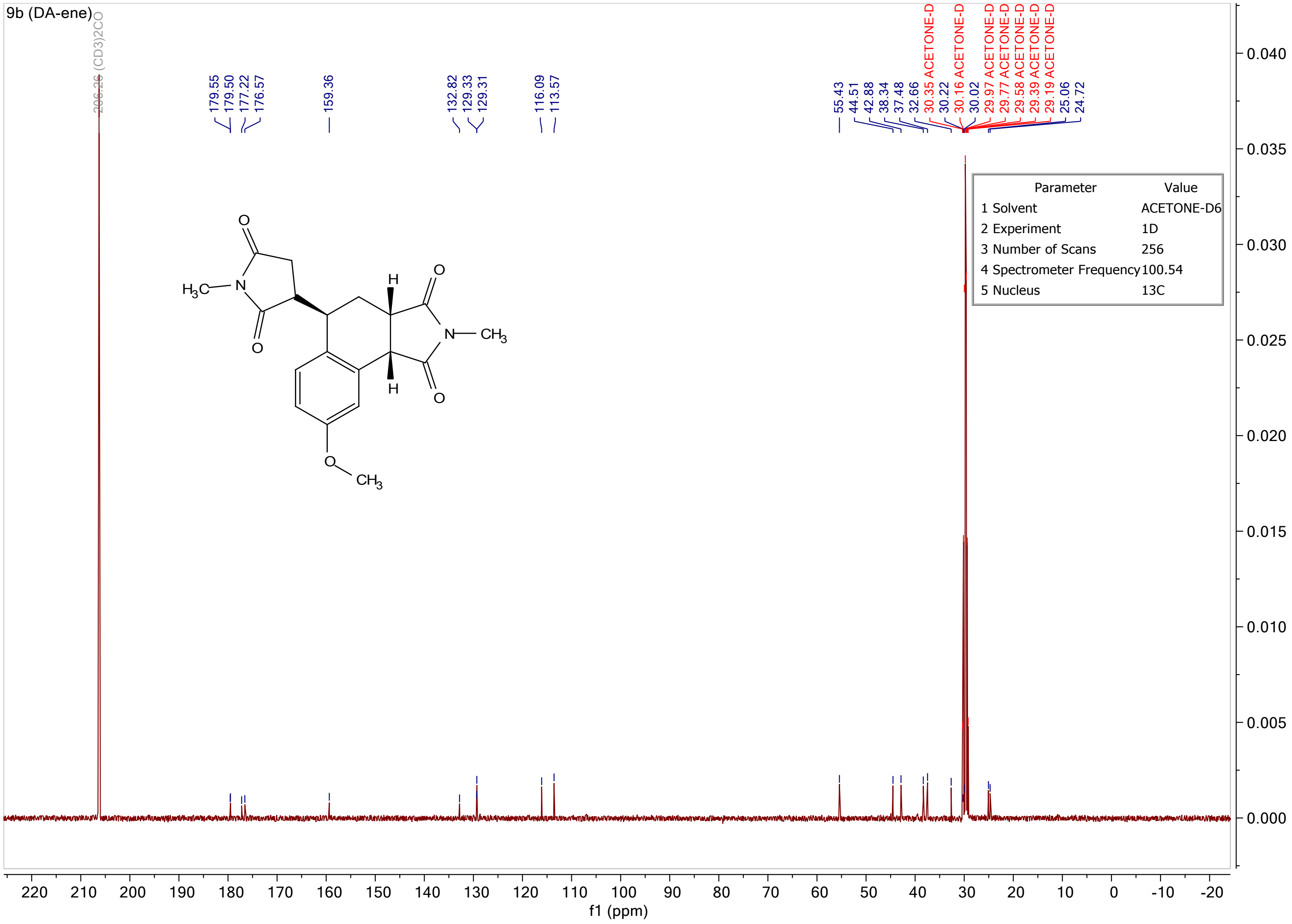

Figure S15. ^{13}C NMR spectrum of DA-ene adduct **9b**.

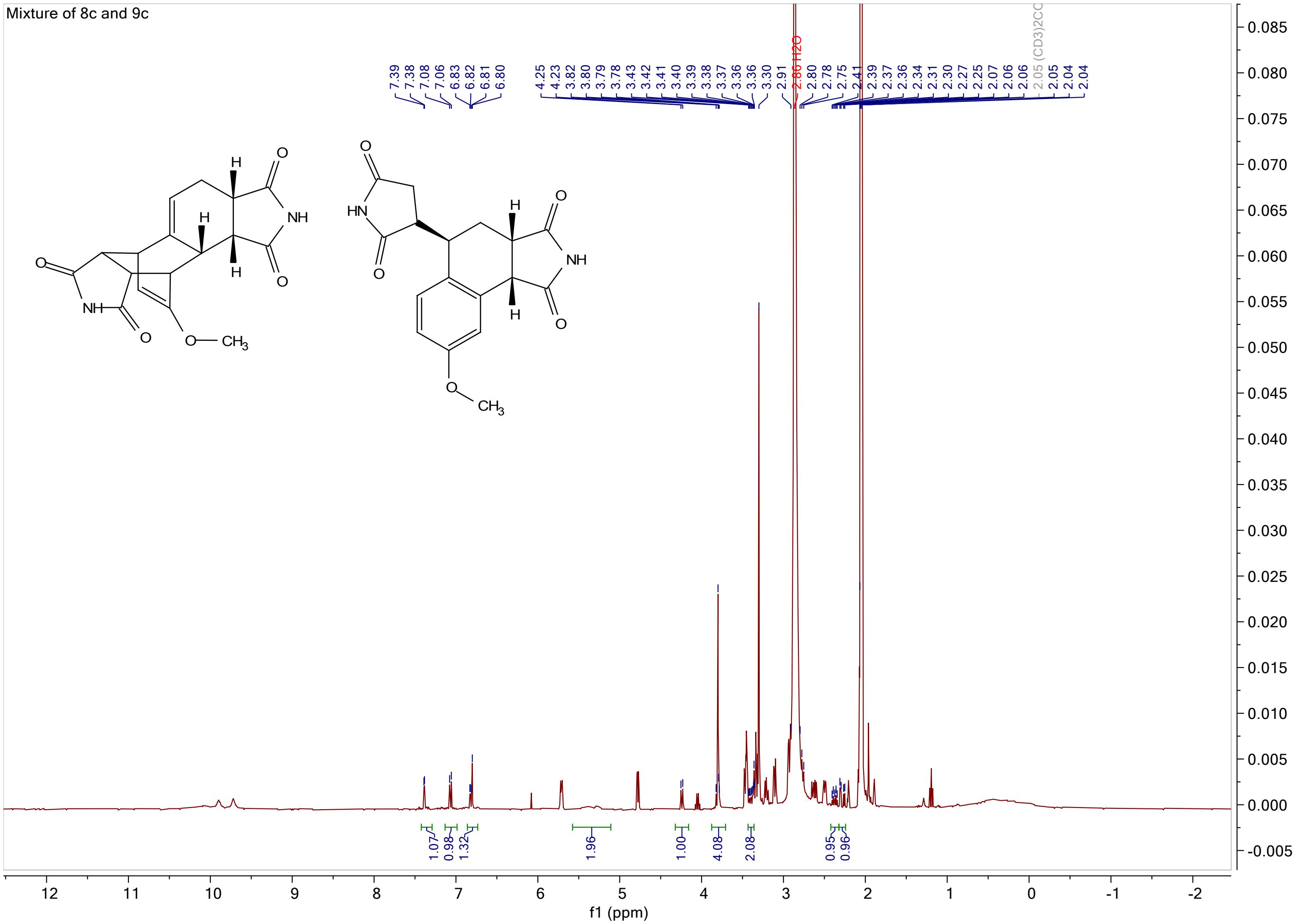

Figure S16. ^{1}H NMR spectrum of a mixture, containing adducts **8c** and **9c**.

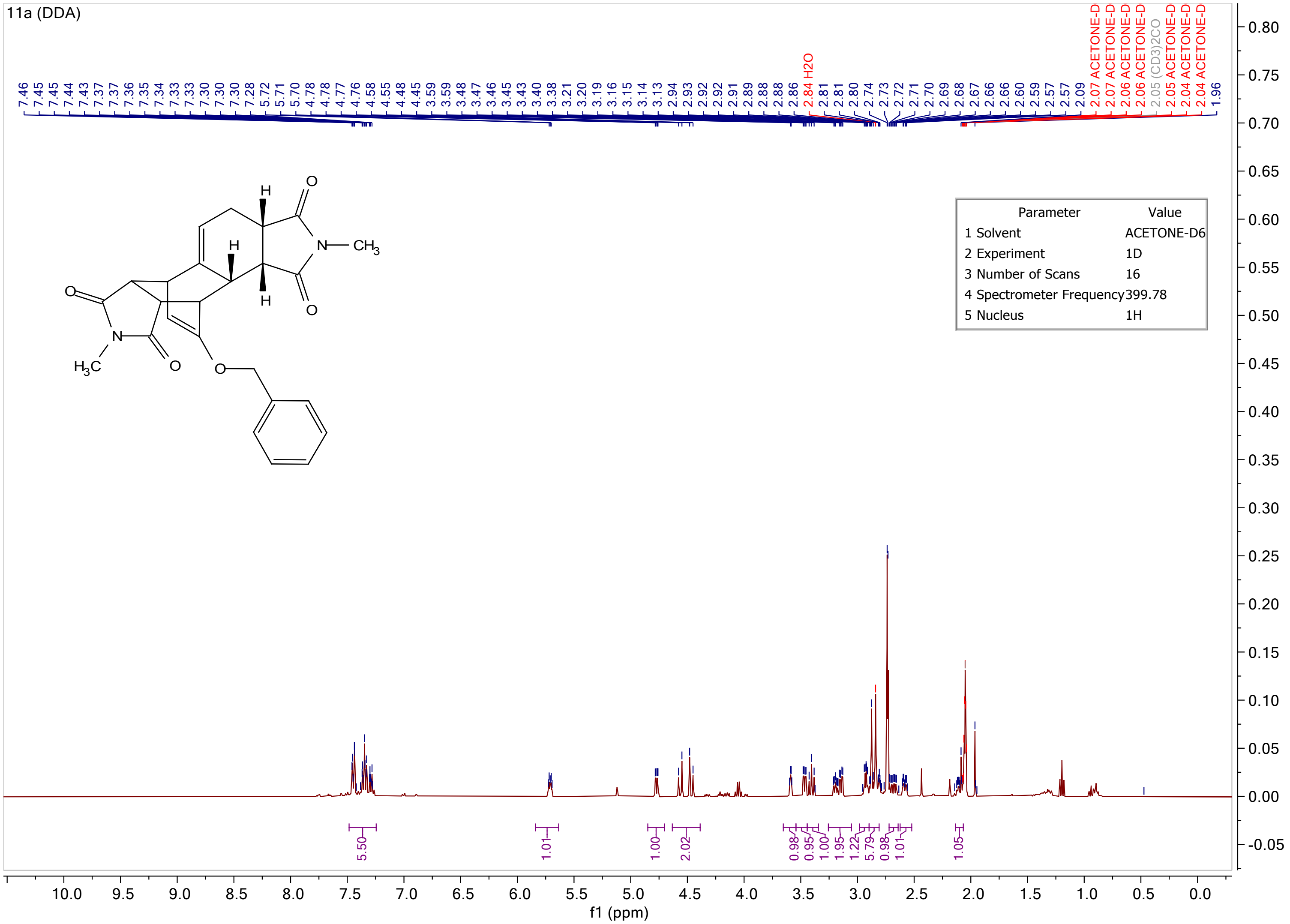

Figure S17. ^{1}H NMR spectrum of DDA adduct **11a**.

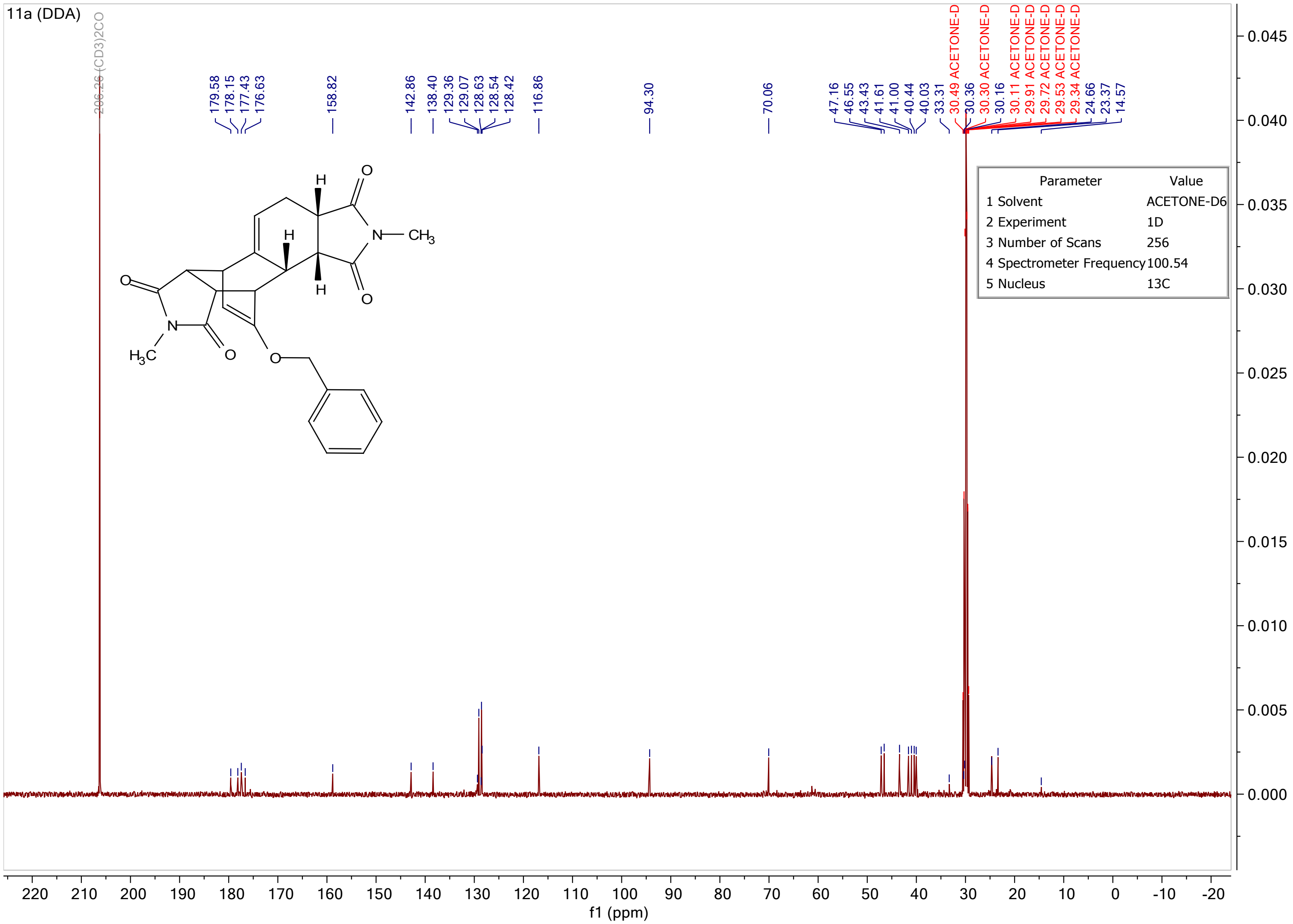

Figure S18. ^{13}C NMR spectrum of DDA adduct **11a**.

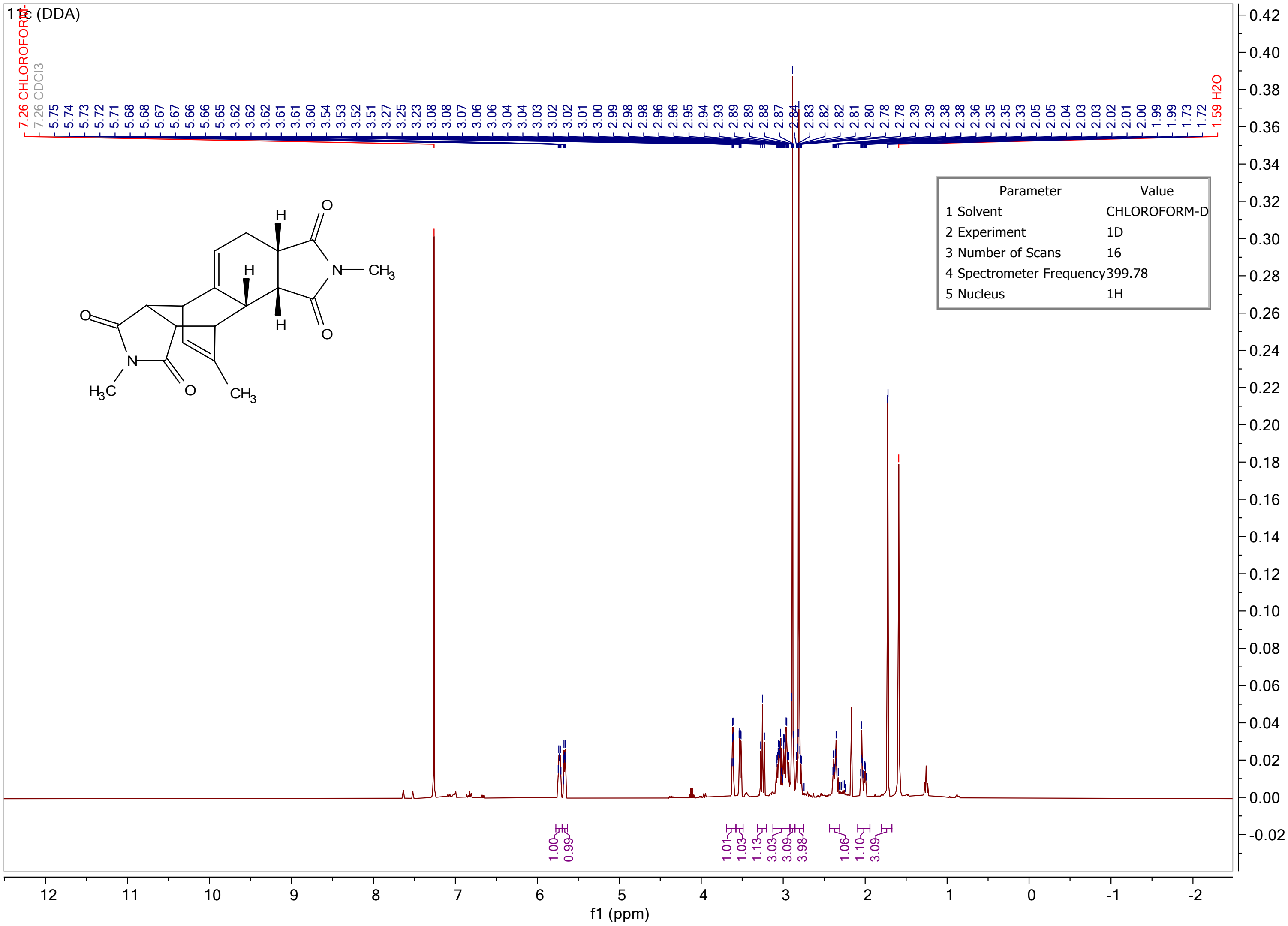

Figure S19. ^{1}H NMR spectrum of DDA adduct **11c**.

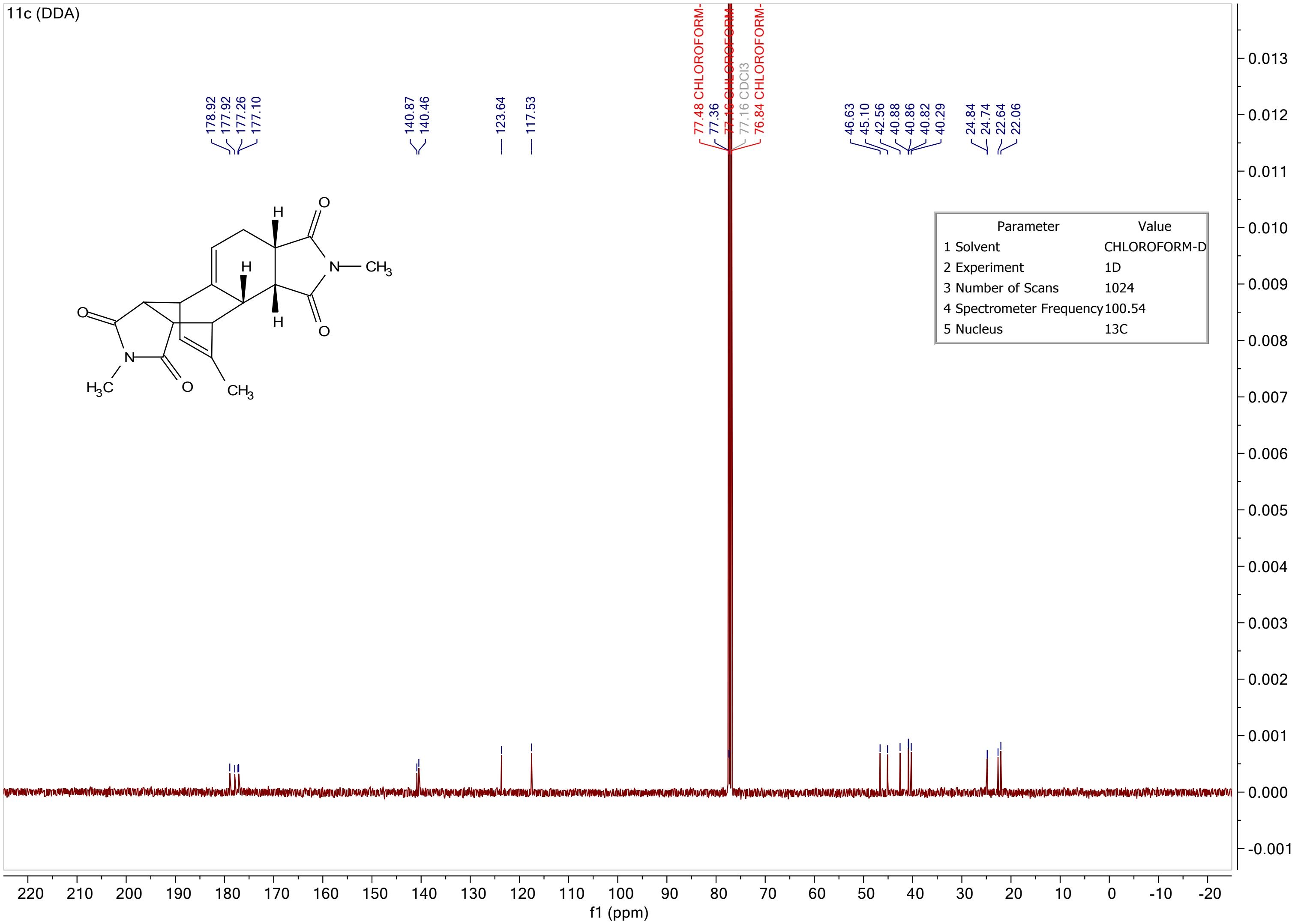

Figure S20. ^{13}C NMR spectrum of DDA adduct **11c**.

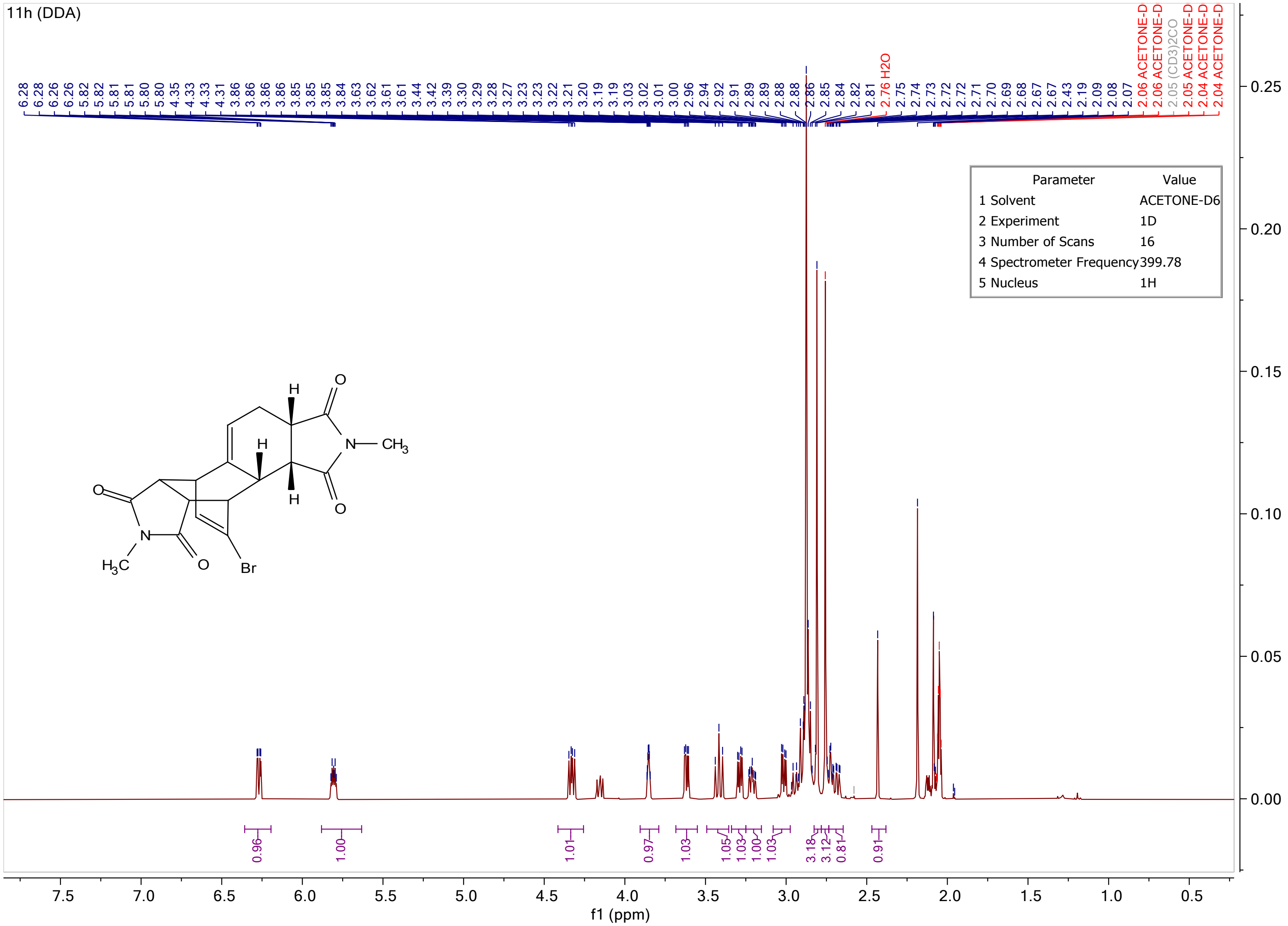

Figure S21. ^{1}H NMR spectrum of DDA adduct **11h**.

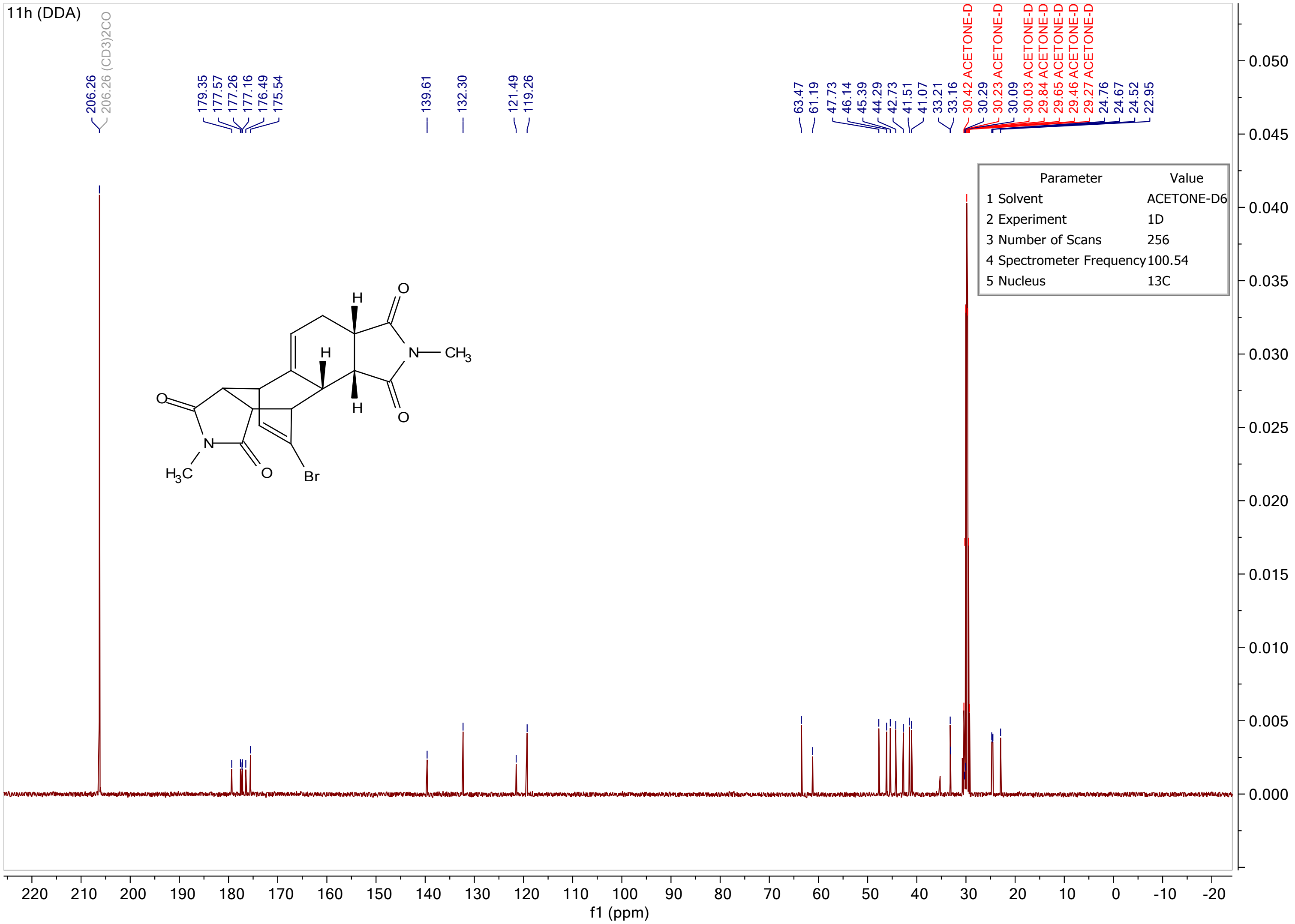

Figure S22. ^{13}C NMR spectrum of DDA adduct **11h**.

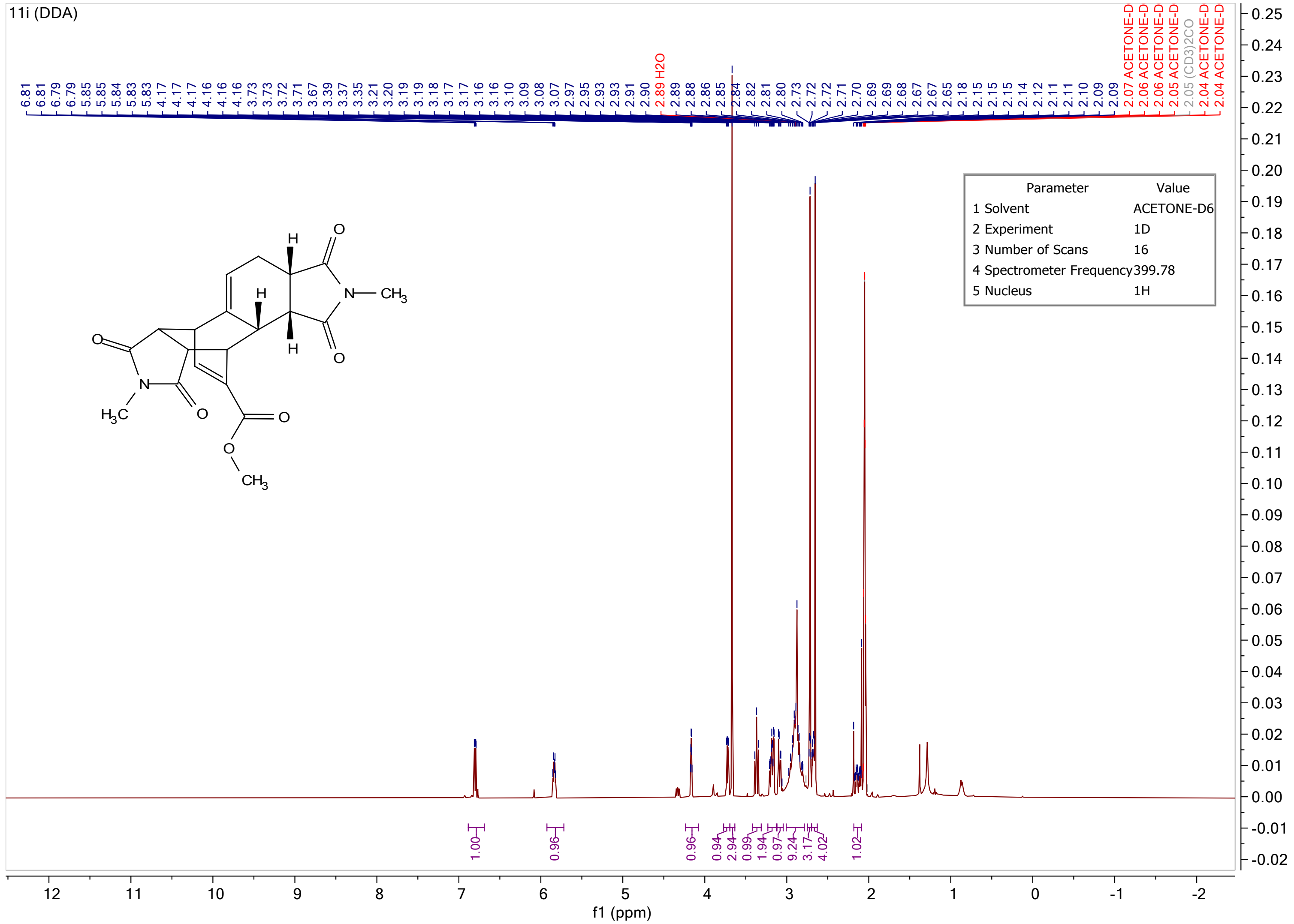

Figure S23. ^{1}H NMR spectrum of DDA adduct **11i**.

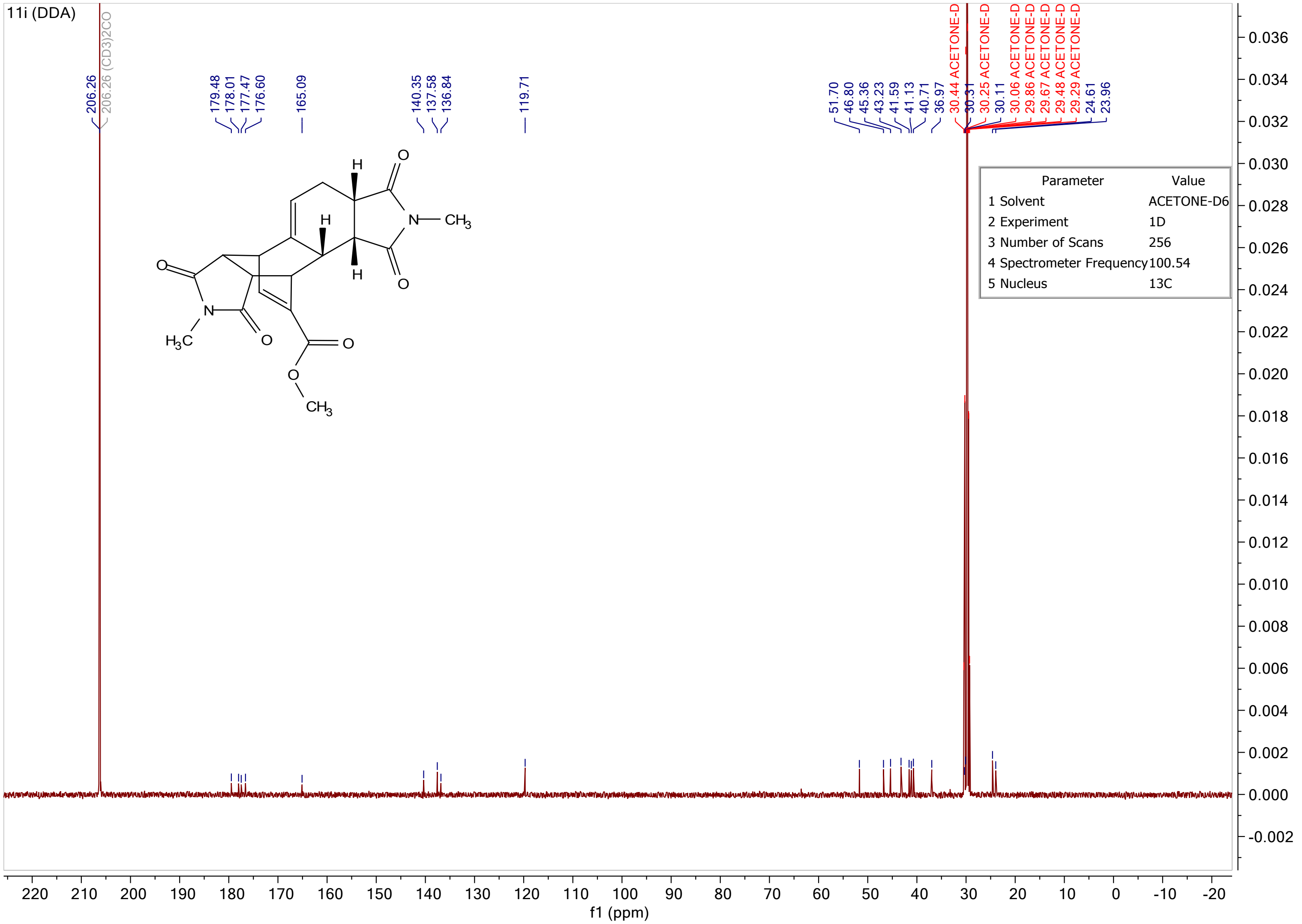

Figure S24. ^{13}C NMR spectrum of DDA adduct **11i**.

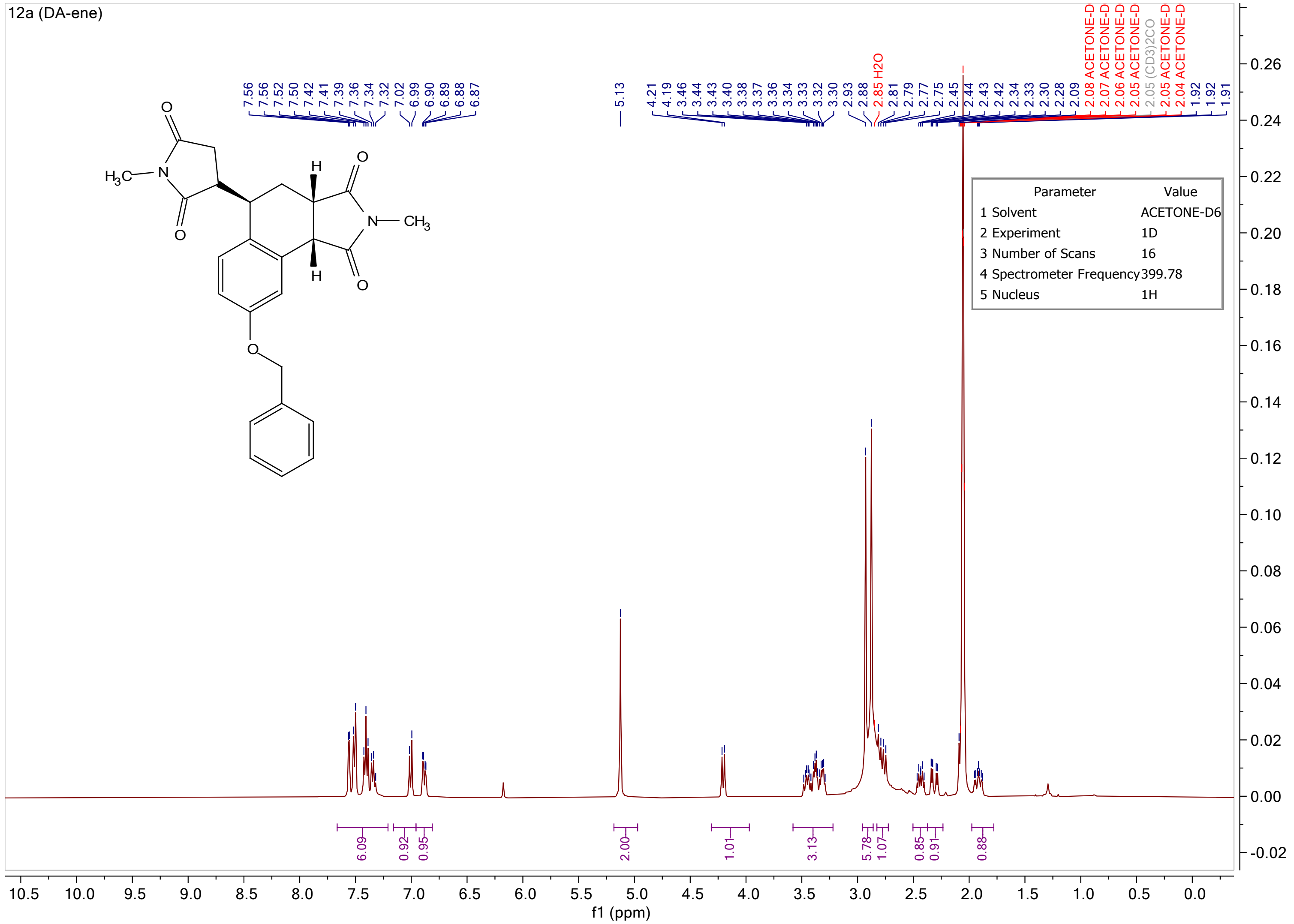

Figure S25. ^{1}H NMR spectrum of DA-ene adduct **12a**.

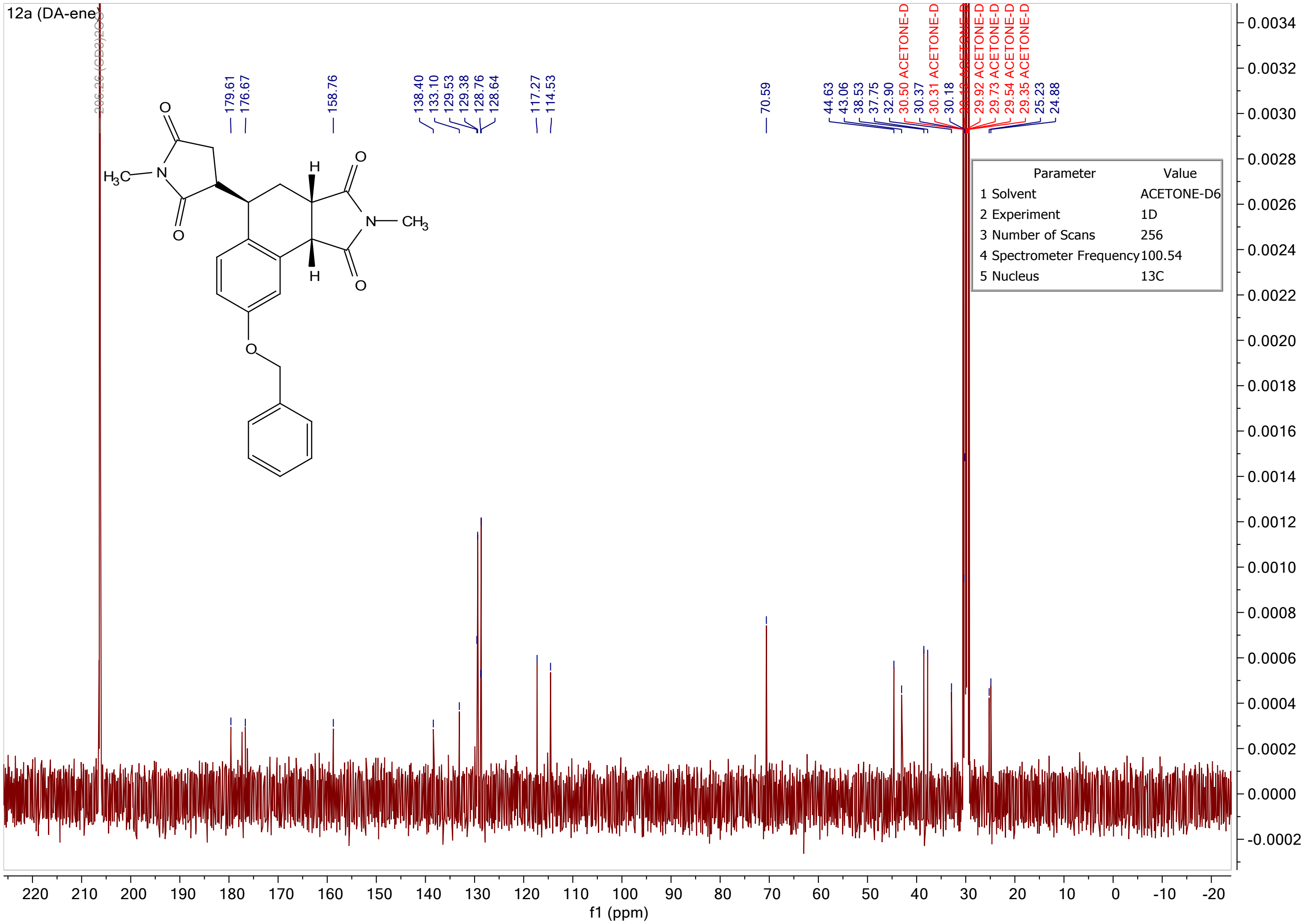

Figure S26. ^{13}C NMR spectrum of DA-ene adduct **12a**.

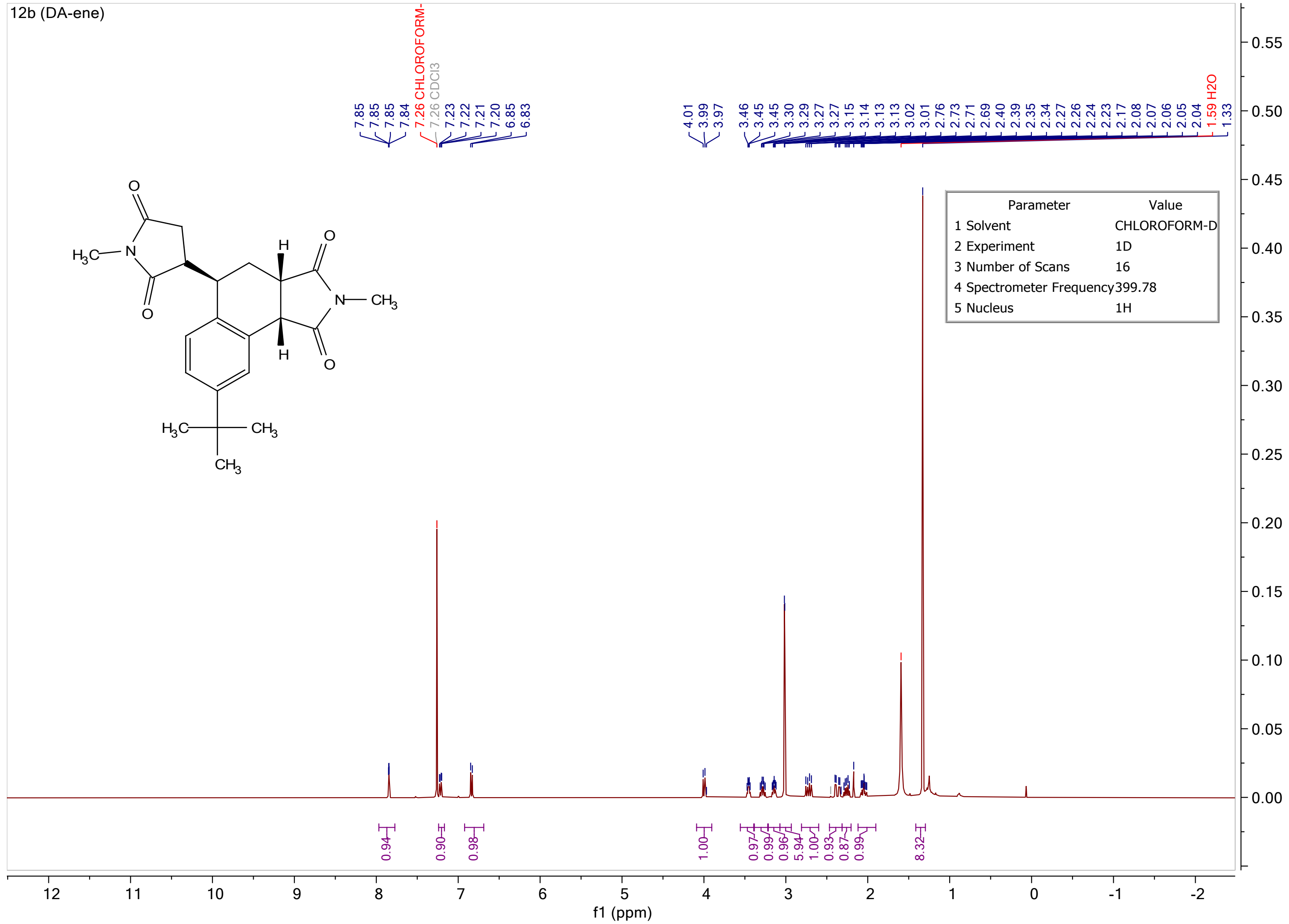

Figure S27. ^{1}H NMR spectrum of DA-ene adduct **12b**.

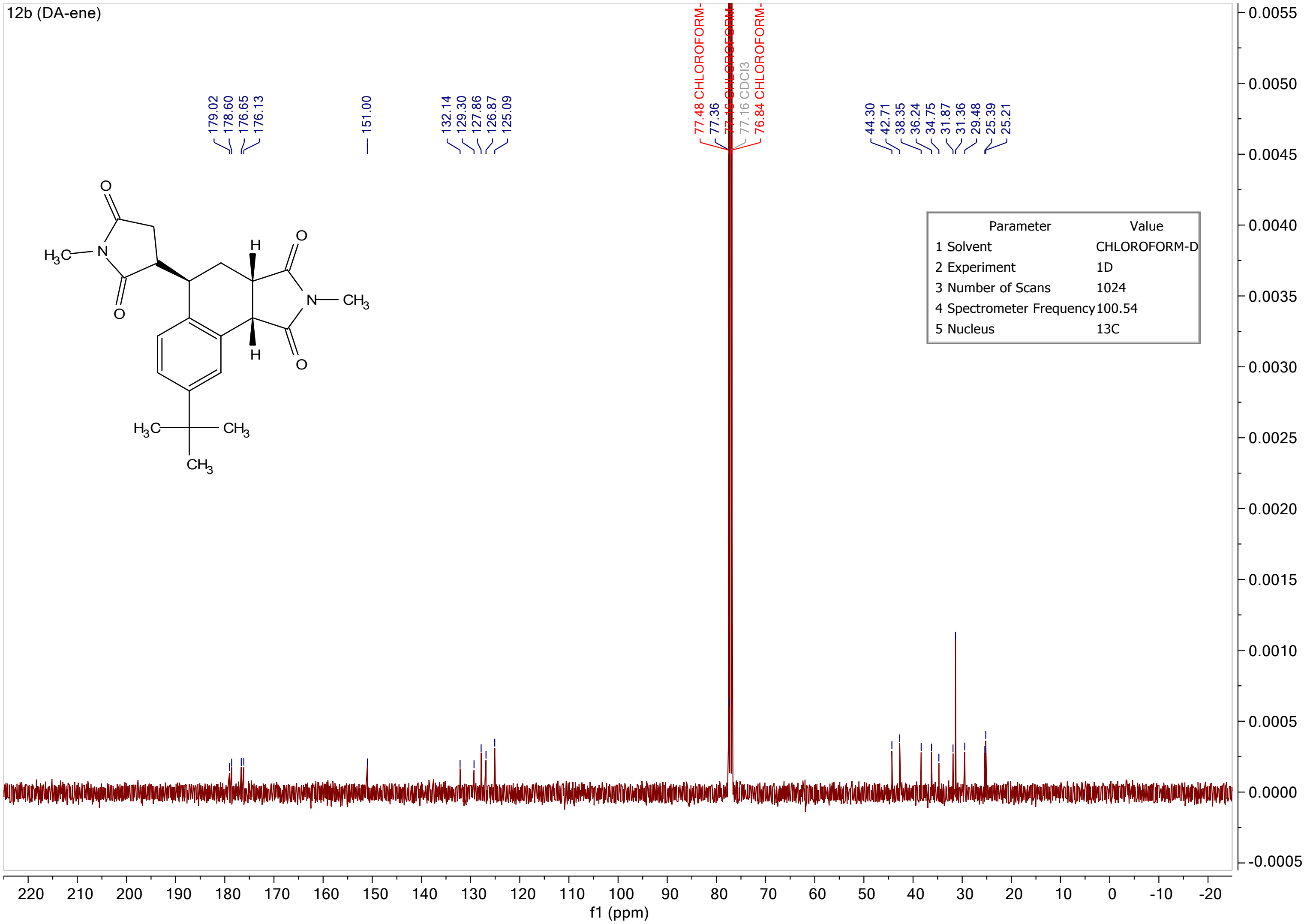

Figure S28. ^{13}C NMR spectrum of DA-ene adduct **12b**.

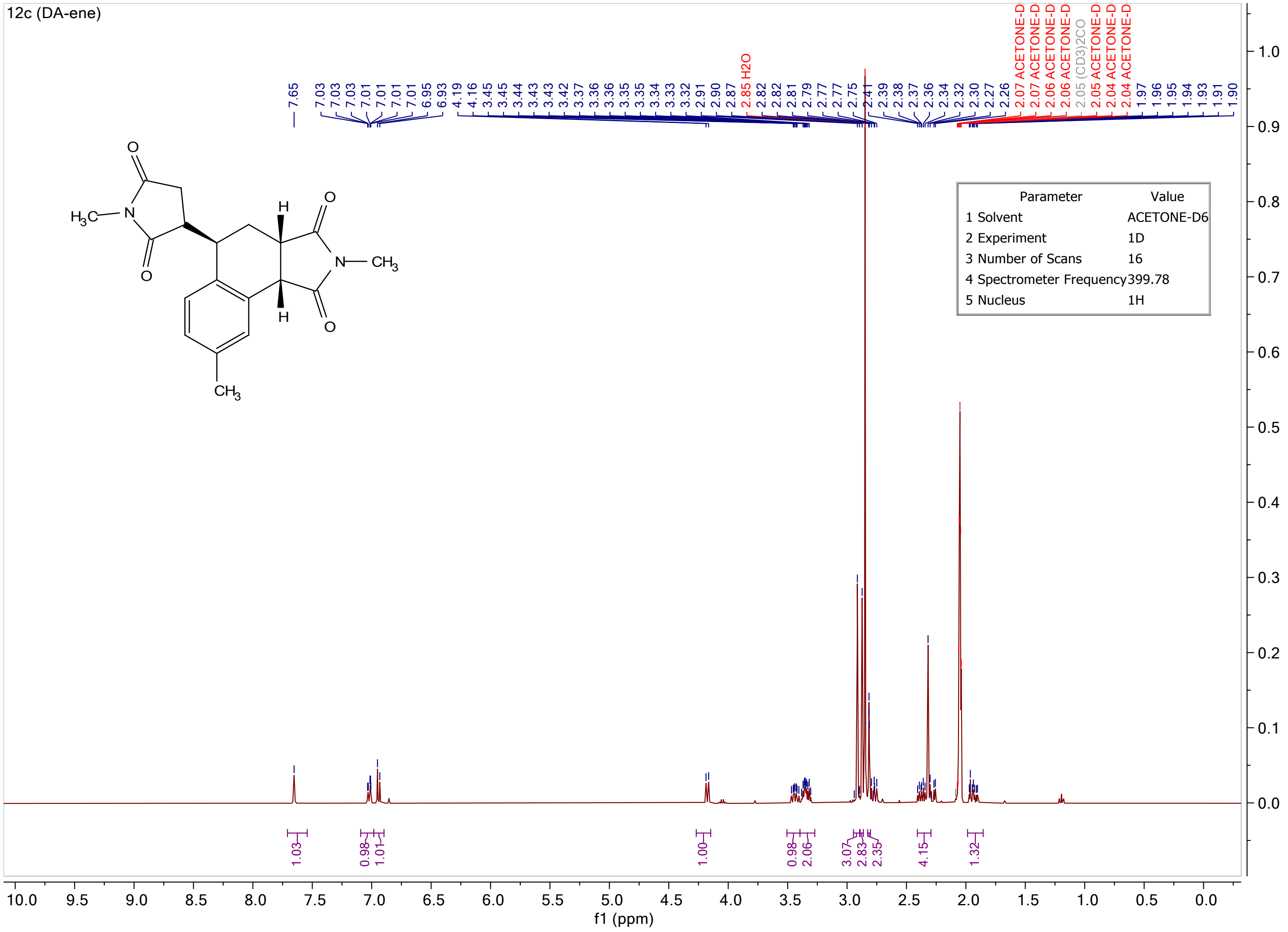

Figure S29. ^{1}H NMR spectrum of DA-ene adduct **12c**.

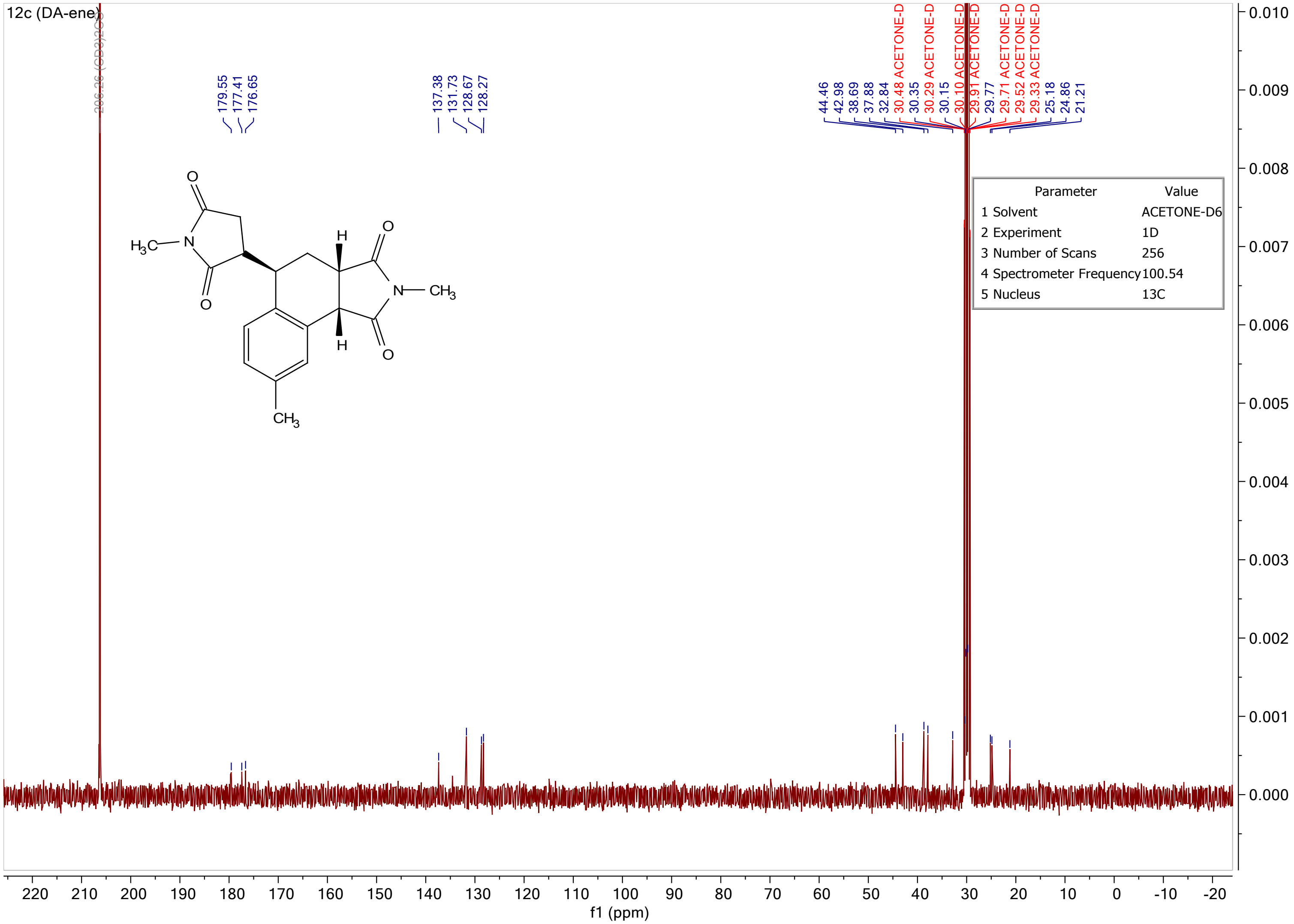

Figure S30. ^{13}C NMR spectrum of DA-ene adduct **12c**.

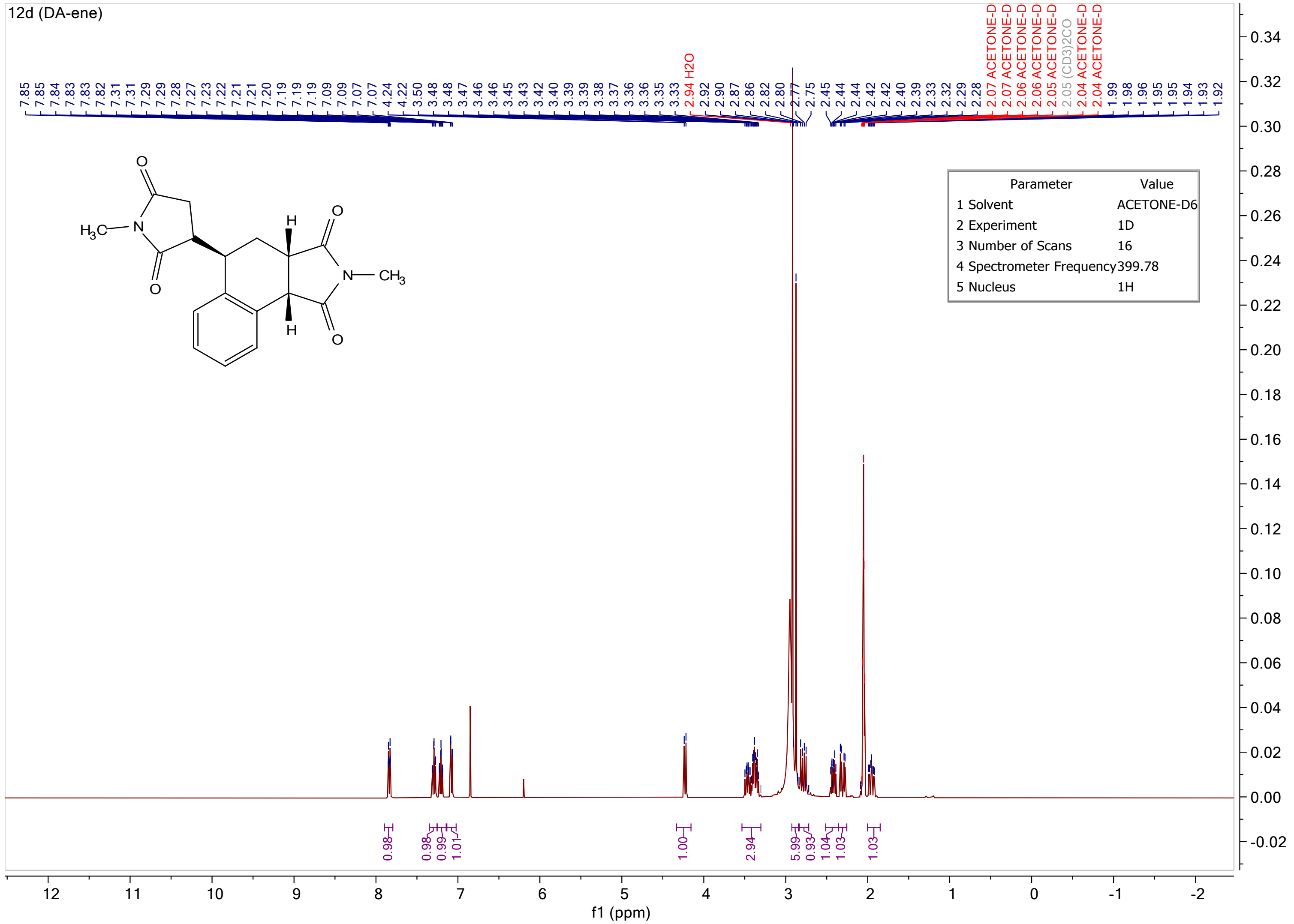

Figure S31. ^{1}H NMR spectrum of DA-ene adduct **12d**.

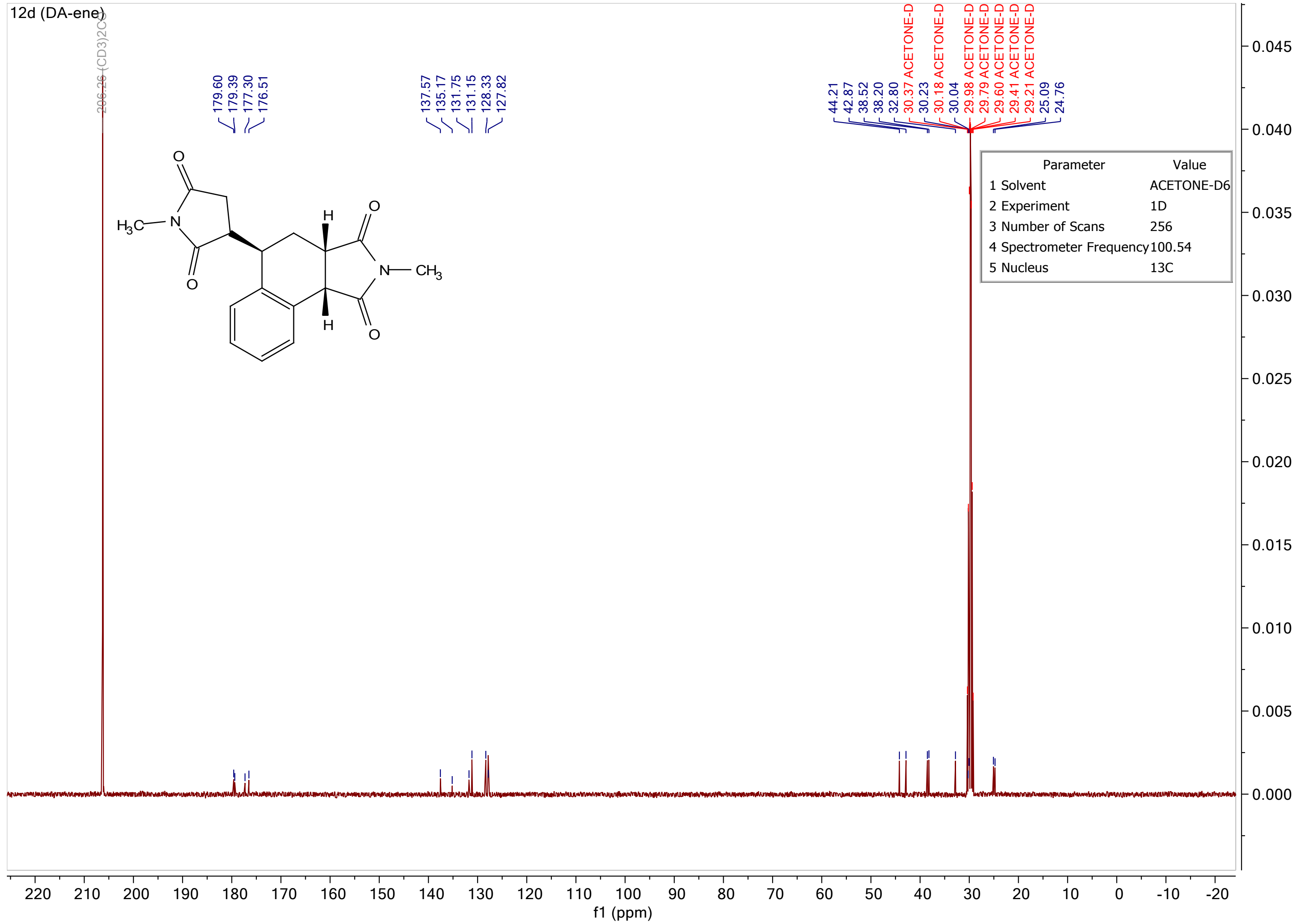

Figure S32. ^{13}C NMR spectrum of DA-ene adduct **12d**.

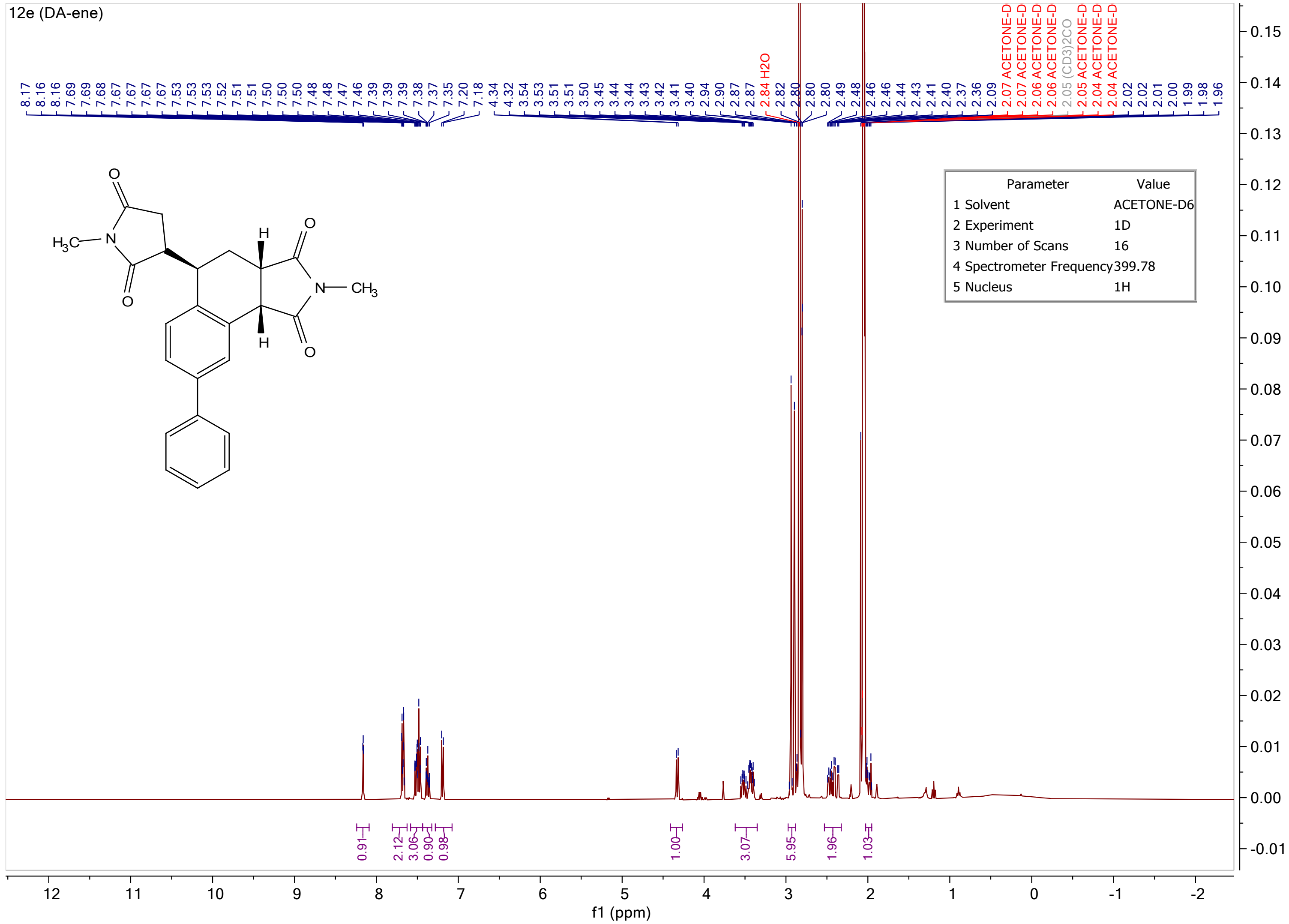

Figure S33. ^{1}H NMR spectrum of DA-ene adduct **12e**.

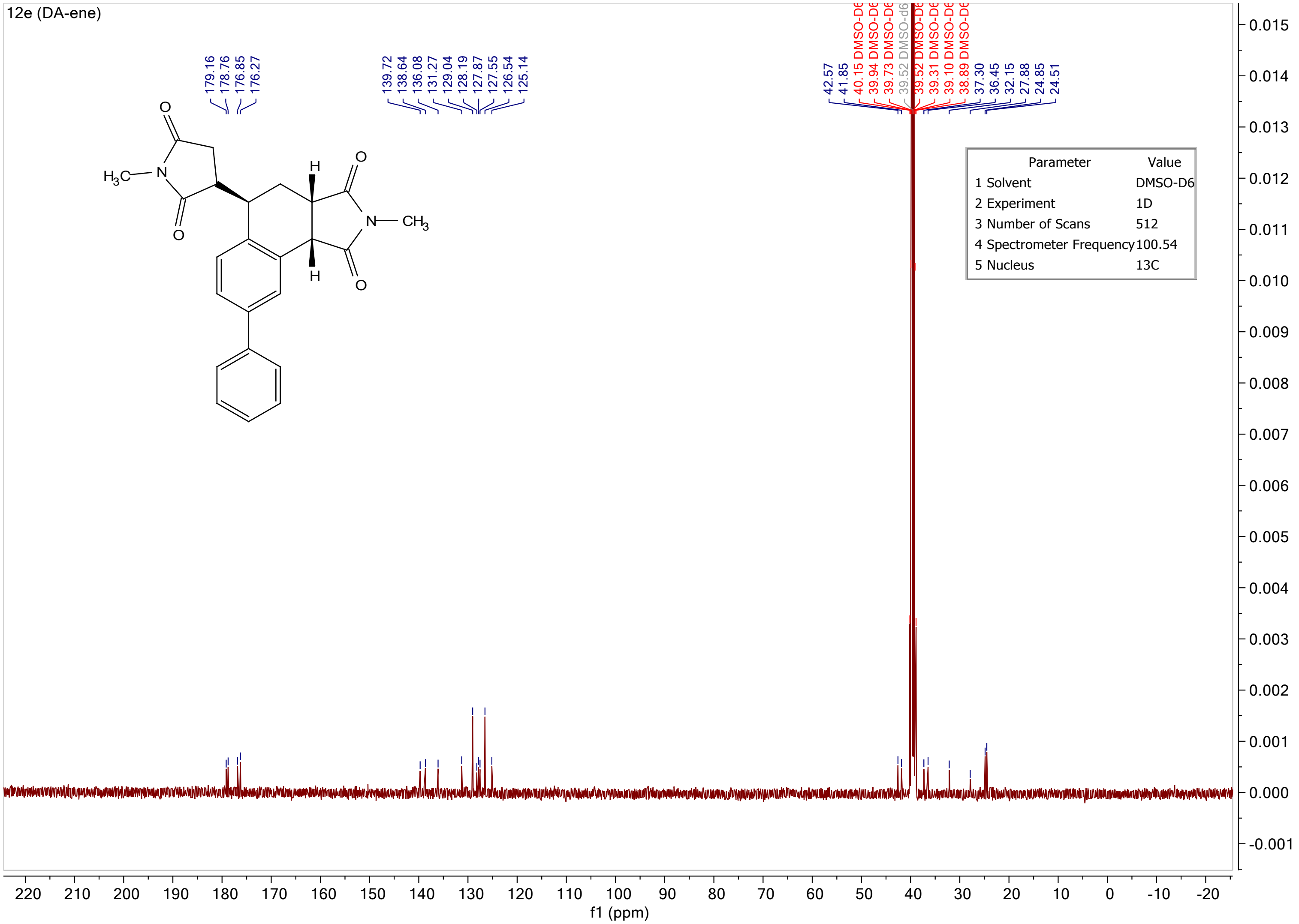

Figure S34. ^{13}C NMR spectrum of DA-ene adduct **12e**.

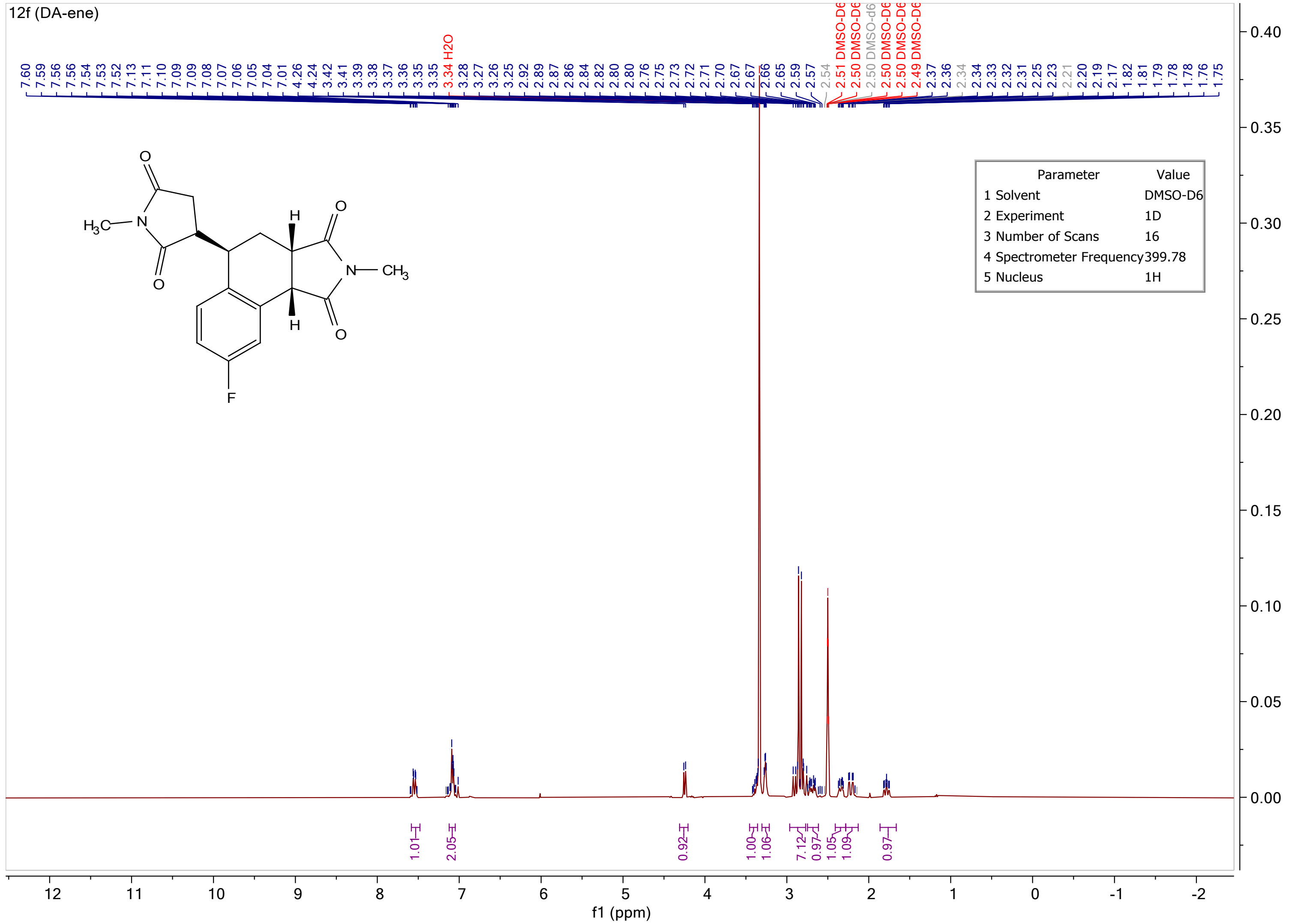

Figure S35. ^{1}H NMR spectrum of DA-ene adduct **12f**.

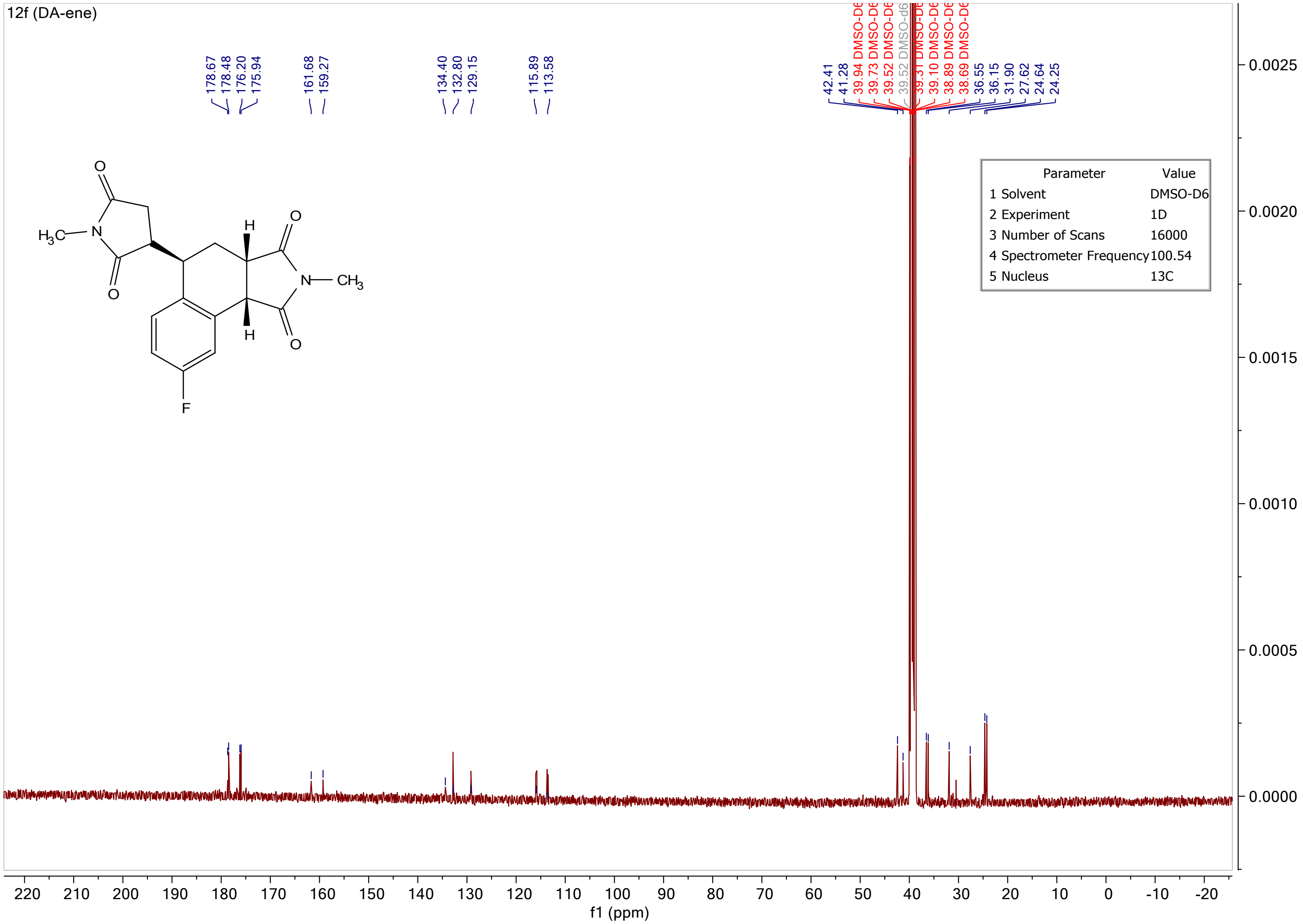

Figure S36. ^{13}C NMR spectrum of DA-ene adduct **12f**.

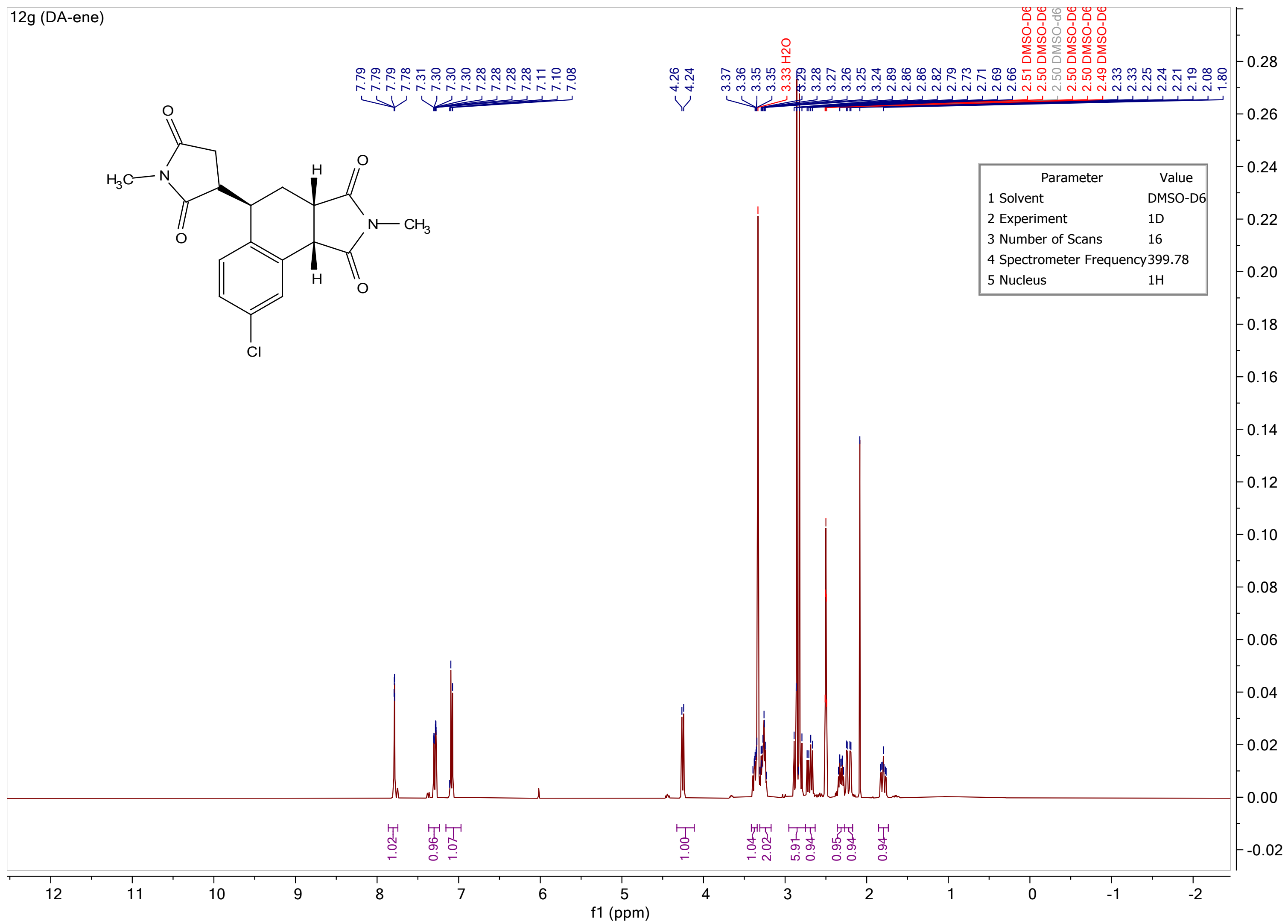

Figure S37. ^{1}H NMR spectrum of DA-ene adduct **12g**.

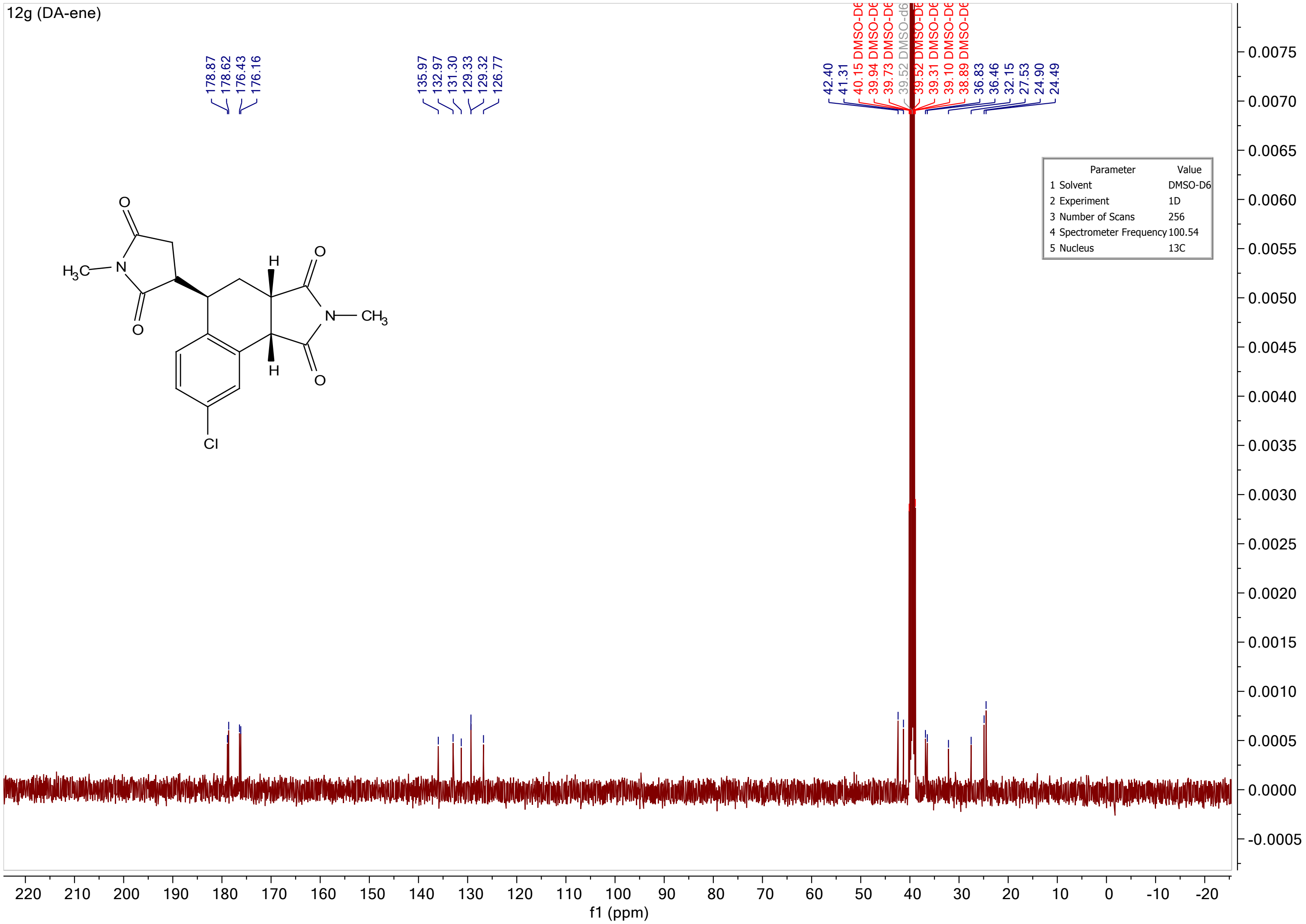

Figure S38. ^{13}C NMR spectrum of DA-ene adduct **12g**.

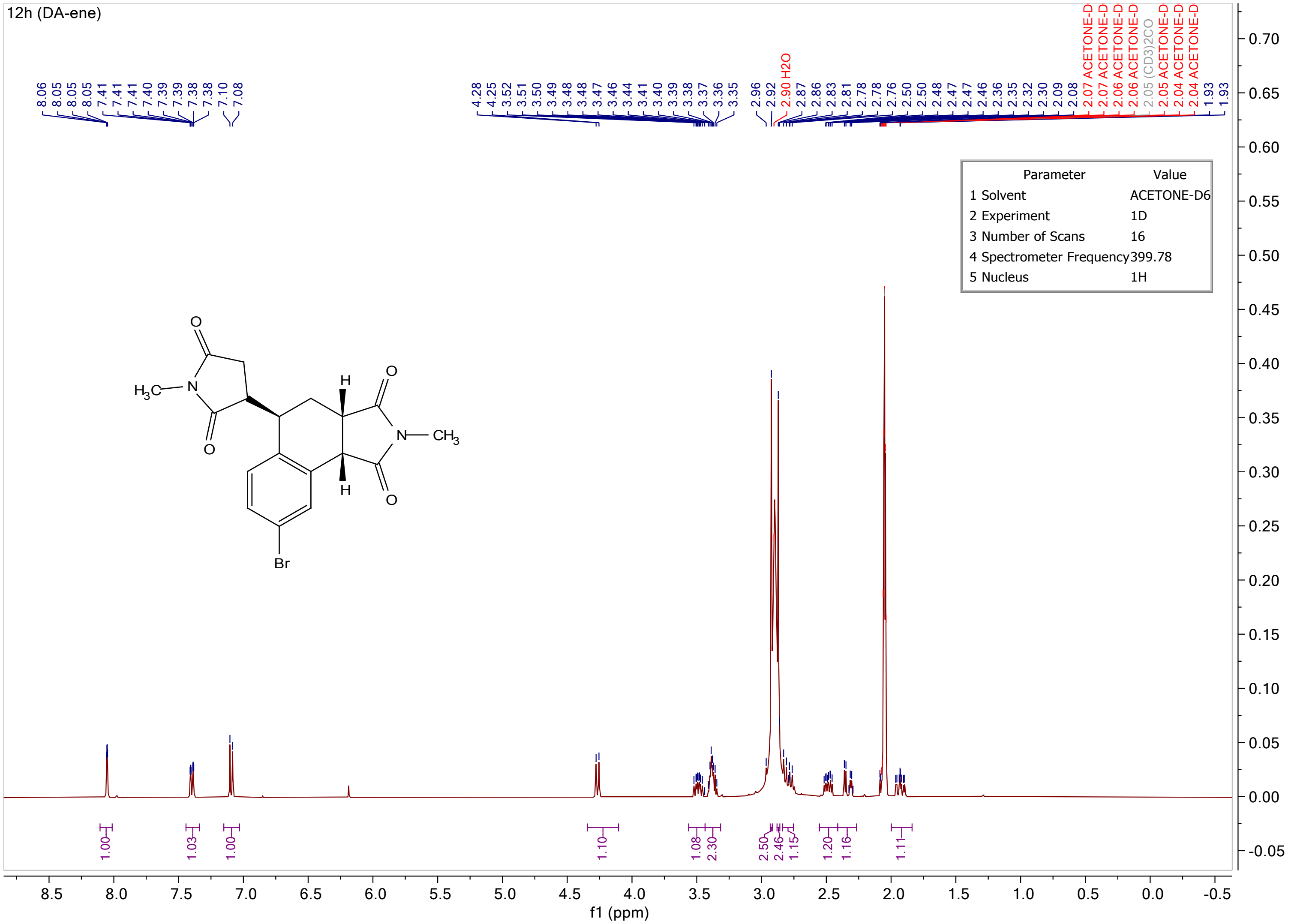

Figure S39. ^{1}H NMR spectrum of DA-ene adduct **12h**.

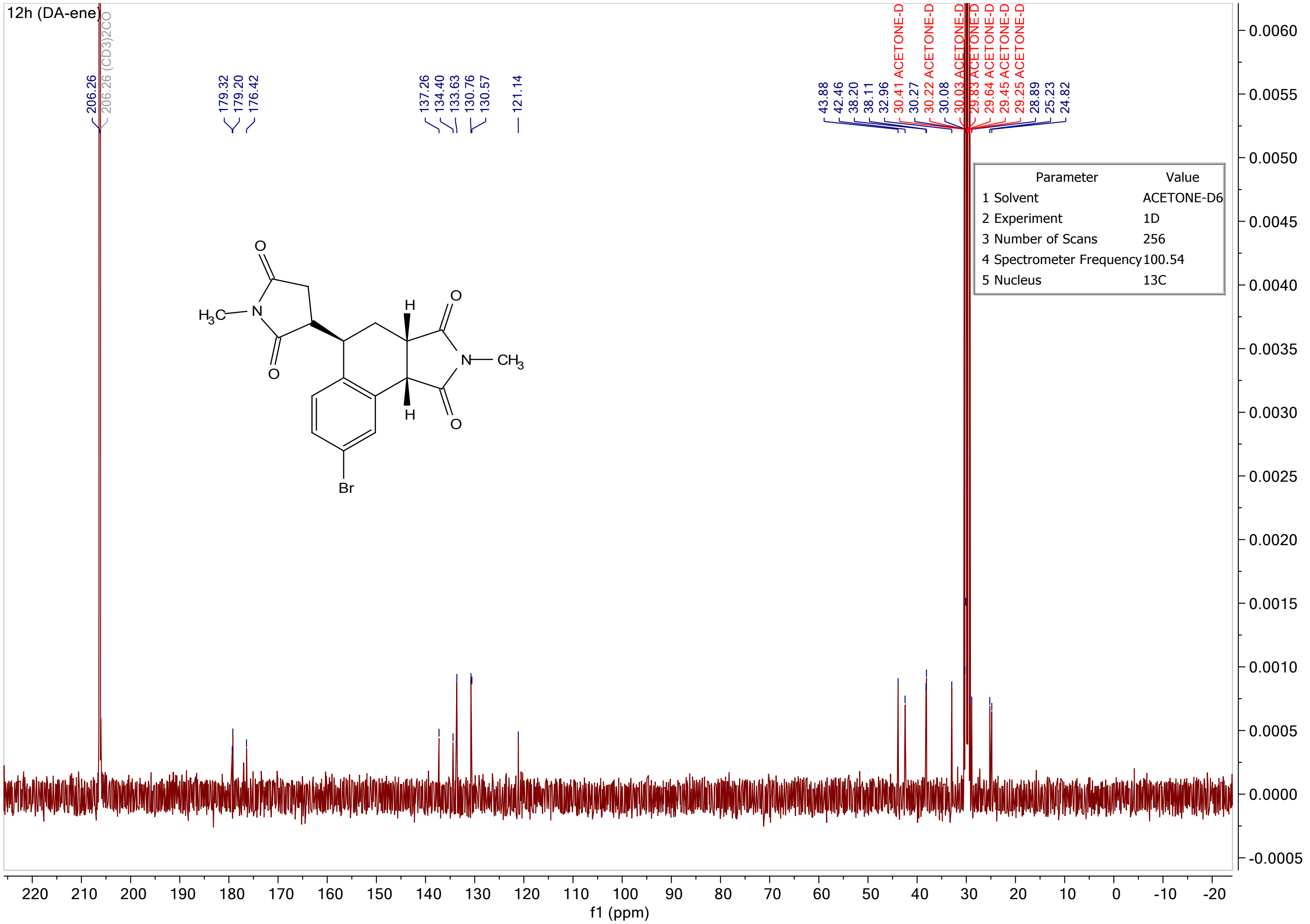

Figure S40. ^{13}C NMR spectrum of DA-ene adduct **12h**.

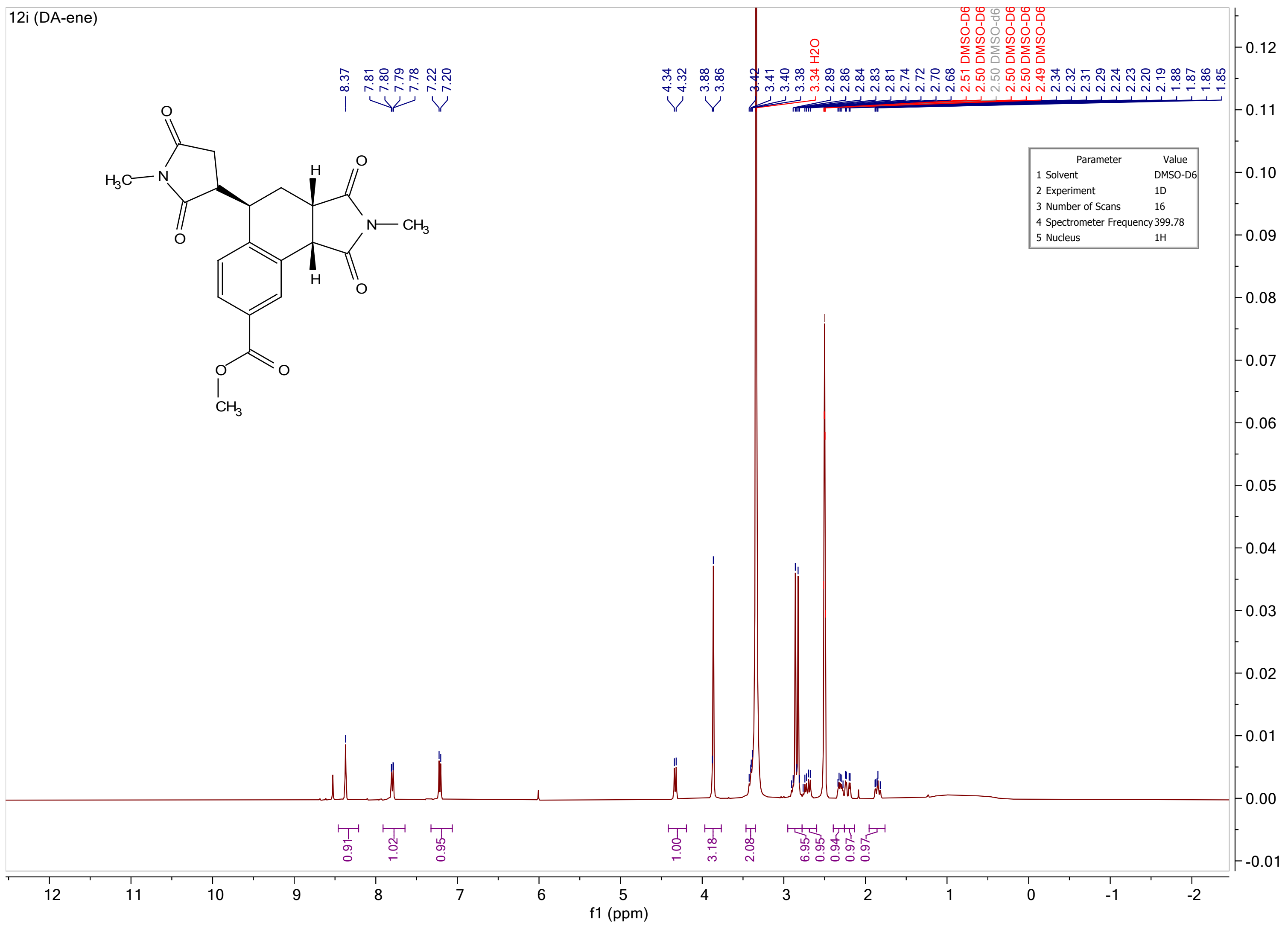

Figure S41. ^{1}H NMR spectrum of DA-ene adduct **12i**.

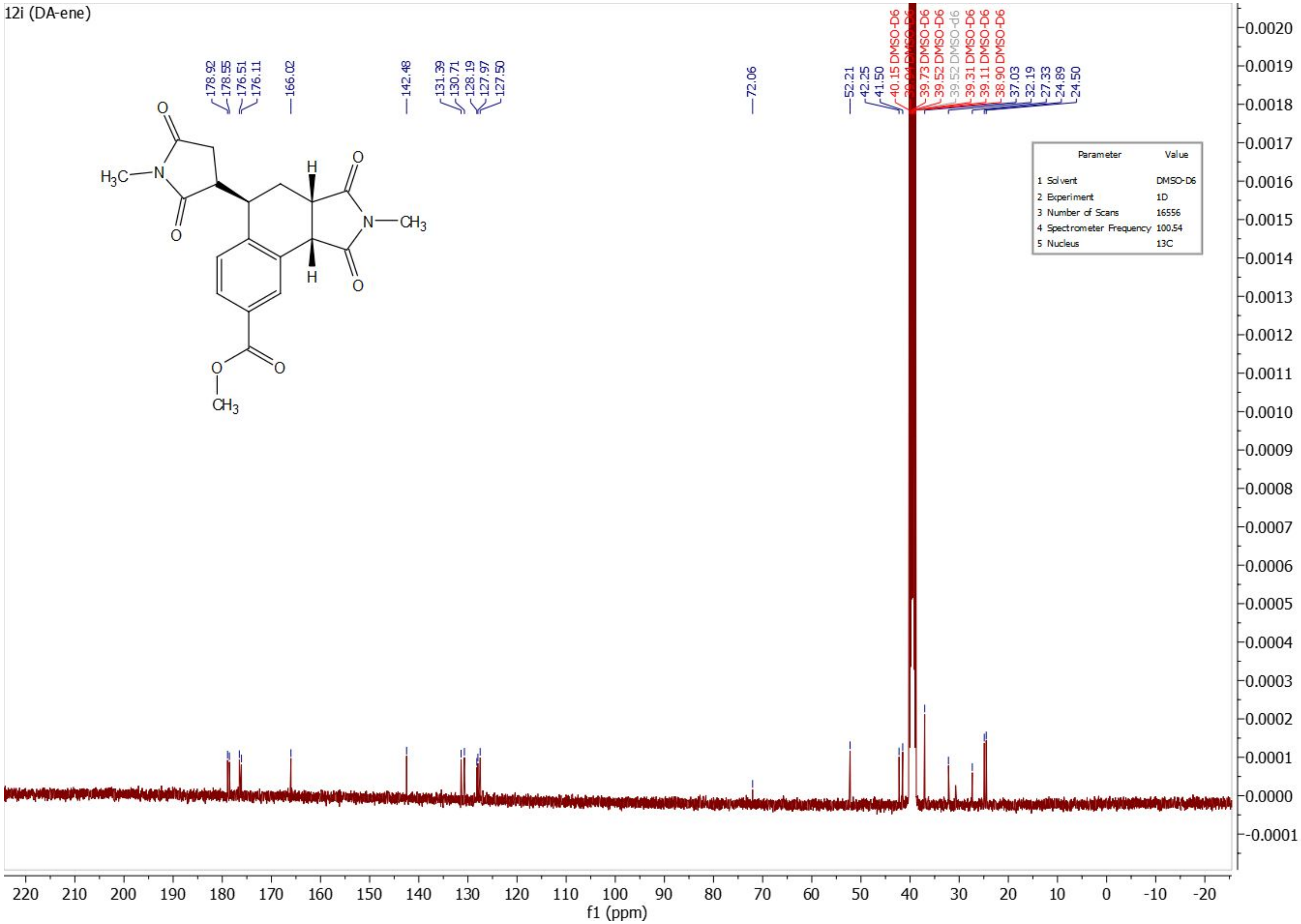

Figure S42. ^{13}C NMR spectrum of DA-ene adduct **12i**.

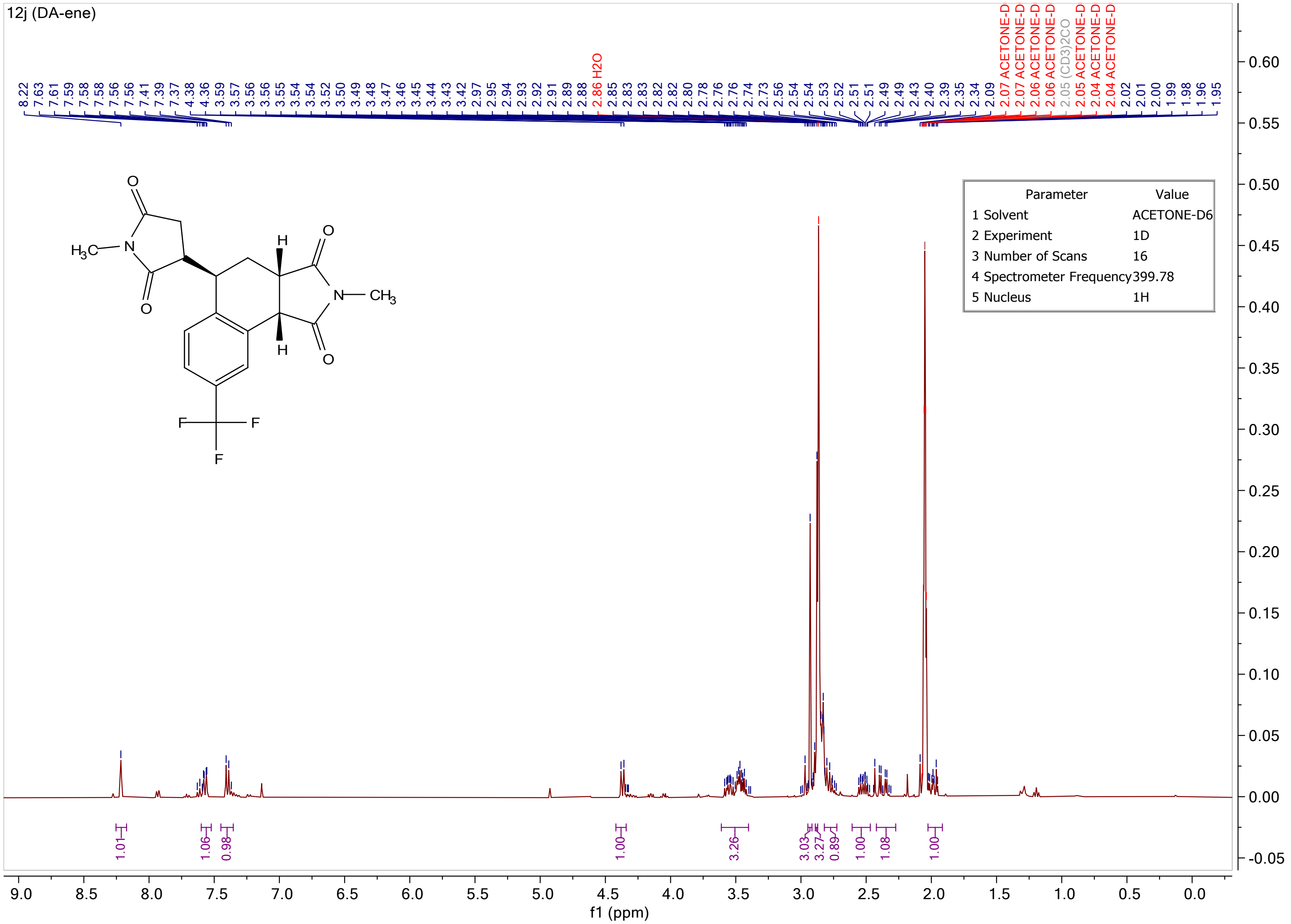

Figure S43. ^{1}H NMR spectrum of DA-ene adduct **12j**.

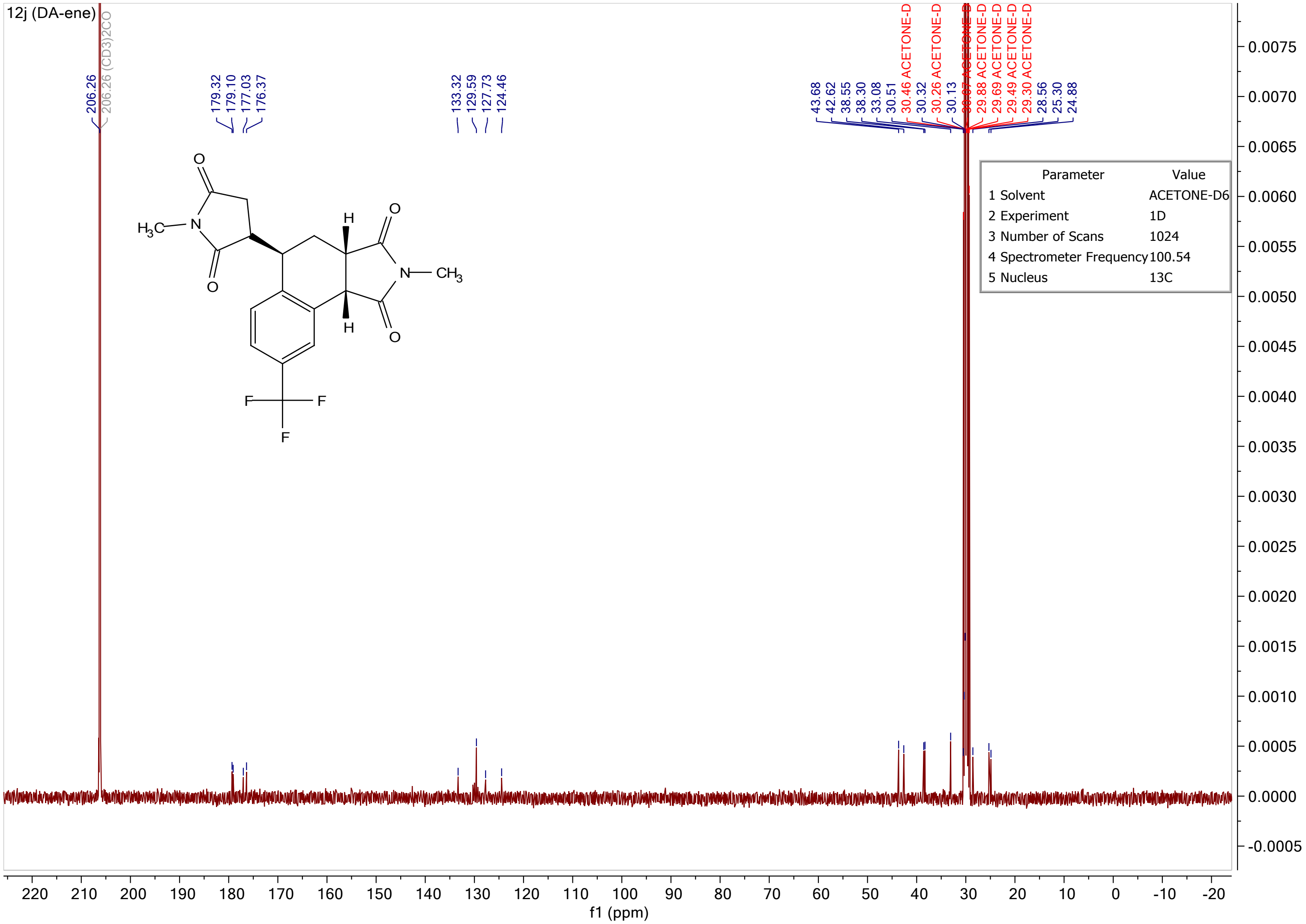

Figure S44. ^{13}C NMR spectrum of DA-ene adduct **12j**.
